# Supplementary material for: Genetic history from the Middle Neolithic to present on the Mediterranean island of Sardinia
Source: Nat Commun. 2020 Feb 24;11:939. doi: 10.1038/s41467-020-14523-6 (PMC7039977; doi:10.1038/s41467-020-14523-6)
Supplement: Supplementary file 1 — Supplementary Information [file 41467_2020_14523_MOESM1_ESM.pdf]

Supplementary Information: Genetic history from the Middle Neolithic to present on the Mediterranean island of Sardinia

Marcus, Posth, Ringbauer et al. (2020)

Supplementary Note 1: Summaries of archaeological sampling

The sub-sections below describe each of the different sites that were sampled for this study (Supp. Fig. 1)

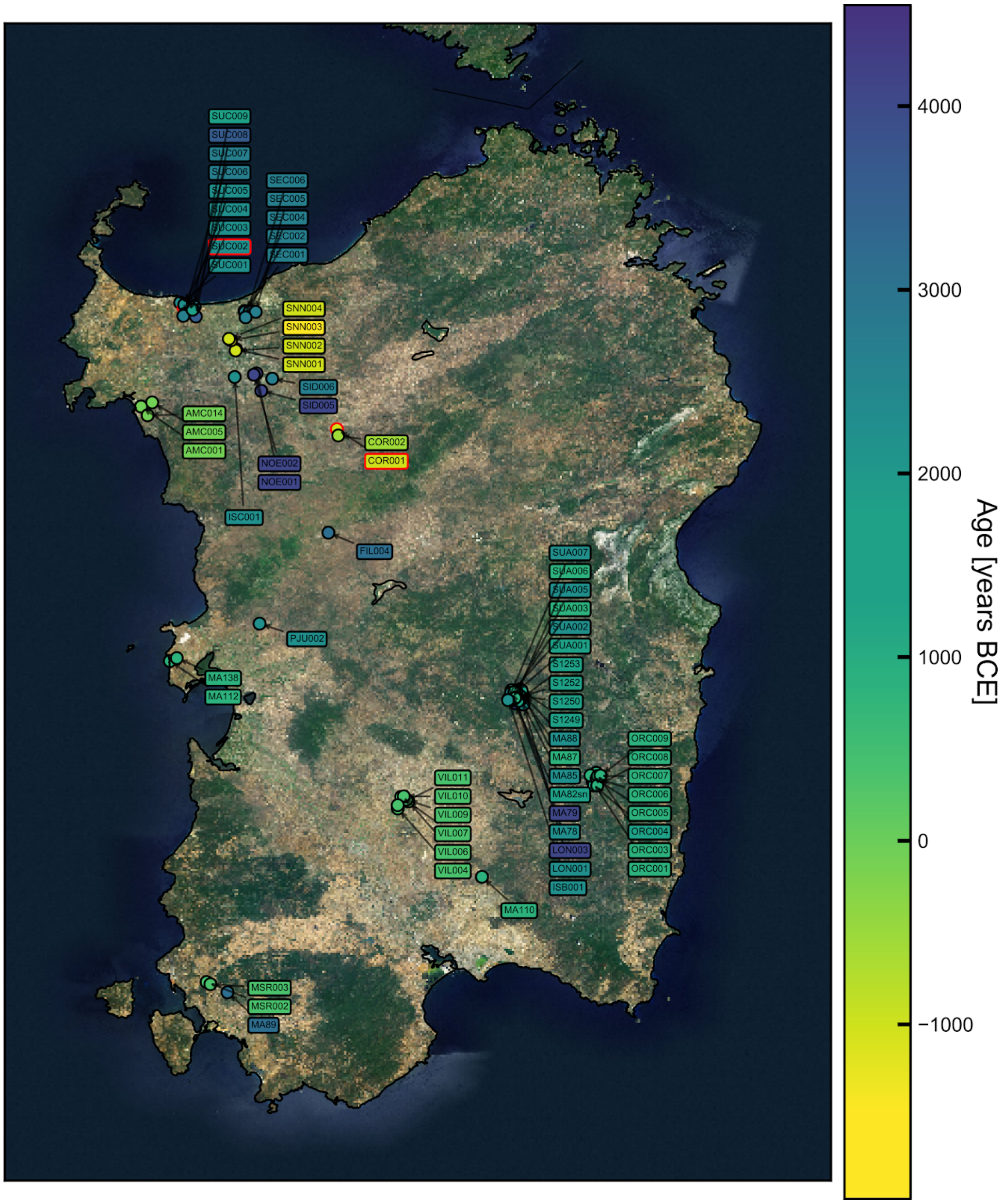

Supplementary Figure 1: Sampling map of ancient Sardinian individuals. We depict a summary of all the individuals used in the study. Individuals are labelled by the identifier and color-coded by age. Samples that were excluded in some analysis because they are first degree relatives of other samples, are color-coded with a red edge. The satellite image is based on data from NASA's Earth Observatory accessed through the basemaps package.

Site Descriptions from the Sampling Effort of Luca Lai

A set of skeletal elements, previously excavated from numerous sites, was organized to form the first major portion of samples in the study with a focus on sampling from the Neolithic to the end of the Bronze Age.

SITE: Is Arutas

The site is a small partially modified natural cave near the seashore and a few miles from the brackish water Cabras Lagoon, in W Sardinia (Cabras municipality, Oristano). Thorough archaeological information regarding the site has never been published; in fact, the context had been looted and mistakenly ascribed to the Late Neolithic-Early Copper Age on the basis of unpublished cultural markers [1].

The remains of 25 individuals were recovered from what was described as both primary and secondary burials. The skeletal material was studied to trace the provenance of the people [1,2] and the analysis suggested a population with diverse morphological features, fairly healthy, and with balanced nutrition. The associated faunal remains, similarly unpublished and apparently lost, included several specimens of *Prolagus sardus* and a whale vertebra [3].

These skeletal remains were sampled for stable isotopic analysis in 2003 (Lai 2014). Oxygen isotopes support a scenario of high variable backgrounds, while AMS dating disproved the attribution to the Neolithic, yielding instead a range compatible with the Nuragic Late Bronze Age (AA-64824, 3054 ± 55 BP = 3382-3079 cal BP 2σ). The two samples used for aDNA extraction yielded virtually identical Late-to-Final Bronze Age dates (MAMS-26896, 2941 ± 27 BP = 3180-3000 cal BP 2σ and MAMS-26894, 2952 ± 25 BP = 3210-3010 cal BP 2σ), which largely overlap with the previous one.

SITE: Ingurtosu Mannu

The human remains that were used for aDNA analysis were excavated by the Soprintendenza per le Provincie di Cagliari e Oristano in 1996, inside a chambered tomb of a typically Nuragic type at about 20 miles North of Cagliari (Donori, South Sardinia). No report has ever been published, but osteological analysis has identified at least 37 individuals of all ages [4] and with specific pathologies [5]. Preservation of long bones was so good that stature could be estimated, and stress markers could be determined for many individuals. The resulting evidence paints a picture of a group subjected to intense physical stress, particularly on the lower limbs. Tissue preservation turned out to be also very good: most individuals had collagen yields higher than 10%, with peaks over 20% of the original weight.

One AMS analysis of bone resulted in a date in the Nuragic Final Bronze Age [1205-910 cal BC: [4], no raw date reported]. A further date comes from sample MA110, used for aDNA extraction (MAMS-26893, 2941 ± 24 BP = 3169-3004 cal BP, 2σ). Even

if the latter is slightly earlier, it largely confirms the Late-Final Bronze Age chronology of the site.

SITE: Cannas di Sotto, tomb 12

The site is a vast cemetery of rock-carved tombs, mostly unexcavated, and located on a low limestone plateau on the edge of the city of Carbonia (SW Sardinia). Survey and partial excavation of tomb 12 were carried out in 1983, although only the corridor and one room of the tomb (room A) were excavated. A preliminary report with site plan and select materials were later published [6]. The human remains were not studied, and a selection of cranial fragments from six individuals were sampled for stable isotopic analyses in 2003, which also yielded the first absolute dating [7]; one of these samples has been analyzed for aDNA for this project.

Excavation was resumed in the inner room of the tomb in 2012, (named room B), where large amounts of human remains were found, which have subsequently been studied and published [8]. The human remains were mostly disarticulated in both rooms and mixed with soil, pottery, lithics, and a few female figurines. Ceramic evidence had already been used after the first excavation to demonstrate that the tomb was in use during the Early Copper Age (ca. 5350-4750 cal BP) [6,9], and the renewed investigations suggest that tomb was already in use from the Middle-to-Late Neolithic transitional phase (ca. 6150-5750 cal BP).

One AMS date on a sample from the 1983 excavation had already confirmed the chronology (AA-64825, 4476 ± 43 BP = 5298-4973 cal BP 2σ: Lai 2009: 318), and a new date from the same batch has yielded a very similar date (MAMS-26903, 4551 ± 26 BP = 5318-5057 cal BP 2σ).

SITE: Filigosa, tomb 1

The site comprises of four rock-carved tombs near Macomer (Nuoro) in central-Northwestern Sardinia. They are of a type that was built between the final Middle Neolithic and the Early Copper Age, but that was in some cases reused until Nuragic times tomb 4 [10]. Tomb 1 is made up of seven rooms and an entrance corridor. No evidence was found that would suggest burial before or after the Early Copper Age. This makes the tomb a rare case that survived intact until the 20th century AD. However, it was excavated in 1965 only after being looted. As a result, even if pottery and human remains were found in substantial numbers, there was no stratigraphic coherence. Only the pottery has since been published [11], but the skeletal remains have only been considered in a study by Germanà [3].

Several mandibles were sampled for isotopic analyses in 2011, and an AMS date was obtained with funding from the Sardinian Autonomous Region (CRP1_661). The date supported the attribution suggested by the associated material culture (OxA-25337, 4401 ± 32 BP = 5213-4865 cal BP 2σ). A new radiocarbon date confirms this chronology (MAMS-38276, 4472 ± 25 BP = 5286-4979 cal BP 2σ). All human

remains are currently under investigation by Dr. C. Rodriguez (Barcelona, Universitat Autonoma).

SITE: Padru Jossu

Accidentally discovered during construction for irrigation purposes, the burial was excavated in 1980. It consists of a large pit, approximately rectangular, with a raised area on one side and a few niches. The excavator believes it to be an underground rock-carved tomb of Neolithic tradition (i.e. *domu de janas*) whose friable roof collapsed. Several layers were recorded, with some individuals partially articulated, spanning three different cultural phases in a quite tight sequence: Monte Claro (a very thin basal layer, with a few bone fragments and potsherds), decorated Bell Beaker (A), and undecorated Bell Beaker (B)[12]. It is possible that the pit, possibly with a perishable roofing, was used in the Final Copper-Early Bronze Age, as would suggest burial ritual at sites pertaining to parallel phases in Southern France and mainland Italy. The importance of the site lies in its wide repertoire of artefacts for material culture aspects which were previously documented only by old excavations carried out without modern stratigraphic standards: among them are pottery, metal items, and ornaments made of shell, stone, teeth[13], and ivory which was recently sourced to both Asian and African elephants[14].

Most of the bone remains have not been analysed, except for 19 crania randomly sampled from the three layers for anthropometric purposes: one from the Monte Claro group, five from the Beaker A group, 13 from the Beaker B group[3 and references therein]. Two out of five Beaker A individuals had atrophy or agenesis of the same tooth, and six out of 19 individuals showed *cribra orbitalia*, likely related to nutritional deficiencies during development. Animal offerings were present in both Beaker layers; caprines were dominant in both layers, with pigs and cattle also well represented, with whole or halved lambs common in phase A; such presence of several individuals from different species made this site important for isotopic analyses, largely unpublished, which indicates heavy reliance on animal products rather than on plants, strengthening the interpretation of the economy and the culture as centred on extensive pastoralism.

Chronology, already suggested based on material culture, was confirmed and refined by radiocarbon dating. Five dates were obtained contextually with the isotopic analyses[7], one of which refers to the first phase (Monte Claro), scarcely represented by human remains (AA-72790, 3912 ± 42 BP = 4509-4184 cal BP 2σ). Two new dates from teeth were carried out within the framework of this study (MAMS-26892, 3912 ± 26 BP, and MAMS-39528). If considered all together, the three dates from Beaker A remains – two of which from the same individual – have a cumulative cal BP 2σ range between 4422 and 4105 BP, whereas the three dates from different Beaker B individuals have a cumulative cal BP 2σ range between 4410 and 4010 BP. The three phases display a large overlap with one another, which could be reduced statistically to approximately one century each, or less, of actual duration.

SITE: S'Iscia 'e sas Piras

The site is located near Usini (Sassari) in northwestern Sardinia . It comprises three rock-carved tombs. One, probably tomb 2, was excavated by E. Castaldi in 1966 after reports of looting. It is a typically Nuragic tomb, although it may have been made by adapting an older Neolithic tomb. The remains of at least 14 individuals were collected and later studied by F. Germanà [15], who classified crania to infer ethnicity, and to investigate lifestyle and health. Stress markers on long bones were interpreted as reflections of a very active lifestyle, possibly linked to herding.

The human remains were later sampled for stable isotopic analyses, and one AMS date was obtained with funding from the Sardinian Autonomous Region (CRP1_661). It supports the attribution suggested by the associated material culture, i.e. the Late-Final Bronze Age (OxA-25338, 2918 ± 28 BP = 3159-2971 cal BP 2σ). An additional date confirms that the tomb was used already much earlier between the Final Copper and the Early Bronze Age (MAMS-38277, 3794 ± 25 BP = 4244-4091 cal BP 2σ). Together, these dates thus suggest that the tomb may contain over a millennium of burials.

SITE: S'Orcu 'e Tueri

The site is a natural cave, located in the territory of Perdasdefogu (Nuoro), in the mountains of Eastern central Sardinia. The site was already known locally, when it was formally recorded in 1963, after looting had been reported. A rescue excavation resulted in the recovery of the best-preserved human remains from the floor of the cave, which have since been kept at the University of Cagliari (Dept. of Life and Environmental Sciences[16]). However, looting continued, and more skeletal remains were recovered by the local speleological association Gruppo Grotte Ogliastra, and in 2014 all the disturbed remains on the surface were recovered in a controlled operation coordinated by the Soprintendenza Archeologica Sassari-Nuoro. These remains are kept in the local museum in Perdasdefogu, and have been sampled for the present aDNA investigation.

The cave had been used for deposition of the dead mostly in Nuragic times, as no other cultural markers were found. While the initial dating was based on physical proximity to a Nuragic tower and settlement, radiocarbon dating has now confirmed the Nuragic chronology. The remains recovered earlier were studied by C. Maxia [16], whereas those recovered recently were studied by P. Martella as part of her PhD research. One radiocarbon date obtained in the 1990s [17] supports the attribution to the Nuragic period (2880 ± 60 BP = 3178-2855 cal BP 2σ). Other dates presented in this study have pushed the period of use back in time, even if still within the Nuragic phase (eight dates covering a cal BP 2σ range 3335-2949). One outlier (individual ORC002) records burial in the cave in times when Carthage controlled Sardinia (MAMS-38281, 2255 ± 22 BP = 2343-2161 cal BP 2σ).

SITE: Serra Crabiles, tomb 3

The site is located in Northwestern Sardinia, near the town of Sennori (Sassari). It consists of a necropolis of at least four tombs of the *domu de janas* type, rock-carved rooms dating to between the final Middle Neolithic and the Early Copper Age. One of them, tomb 1, yielded human remains which were attributed to the Late Copper Age (Monte Claro culture) based on the association with pottery sherds, even if there was no reliable stratigraphy, and despite the reported presence of a later Bell Beaker decorated sherd [1]. Tombs 2, 3 and 4 were excavated by the Soprintendenza Archeologica Sassari-Nuoro in 1981 [18] and in 1993-94, when several additional rooms connected with tomb 4 were discovered [19].

The human remains sampled for aDNA extraction come from tomb 3, which yielded potsherds attributed to the Monte Claro phase, but also Bell Bell Beaker cultural markers. Radiocarbon dates obtained from the human remains leave some uncertainty, as their chronology covers the interface between the two phases (five dates with a cumulative cal BP 2σ range between 4422 and 4151). The best fitting overlap is nevertheless with the Bell Beaker dates from Padru Jossu [7], and some unpublished ones from Bingia 'e Monti.

SITE: Corona Moltana/Zarau, anfratto 1

The site is located in Northwestern Sardinia, near the town of Bonnanaro (Sassari). It consists of a vast complex of at least ten rock shelters, small caves (*anfratti*) and artificial rock-cut tombs (*domus de janas*), all within a few hundred meters from each other, at different heights and locations on a slope below a limestone plateau. Only tomb 1 had been found and investigated early in 1889, with grave goods found intact. It was dated to the Early Bronze Age, which led to the definition of the so-called Corona Moltana/ Bonnanaro ceramicic facies; all other tombs were investigated in 2000 by G. Meloni, who also published most of the available information [20].

No associated cultural material was found when the skeletal remains of several individuals were recovered from the two small caves (*anfratti* 1, 2), but the burial ritual was compatible with the Early Bronze Age, as proposed in the early-1900s. As a result, the chronological attribution remained tentative. The investigator had suggested later usage of the caves, based on oral knowledge in the nearby town, which claimed that the cave had been used as a dwelling by a family stricken by some infectious disease, which would have led to their death at the site [20].

Radiocarbon dating of two individuals from Anfratto 1 confirmed a presence in historic times, with dates falling within the Early Middle Ages (MAMS-38278, 1124 ± 23 BP = 1057-984 cal BP 2σ; MAMS-38279, 1182 ± 21 BP = 1172-1066 cal BP 2σ). This placement provides an important intermediate reference point, though limited to a few individuals, for the evolution of the genetics of Sardinian populations between ancient and modern times.

SITE: Su Crucifissu Mannu, tomb 16, tomb 22

The site is located in Northwestern Sardinia, just outside modern Porto Torres . It is one of the many reused burial areas surrounding a unique ceremonial site dating to the Late Neolithic-Early Copper Age. It comprises rock-carved tombs composed of several interconnected rooms [21]. The necropolis was excavated in a number of campaigns between 1958 and the early 1970s. The best-documented tomb is t.16, which yielded at least 13 individuals. Most of them were attributed to the Early Bronze Age 1 based on associated material remains and a clear stratigraphy, with the possibility that some individuals from rooms D and especially E could belong to an earlier Monte Claro or Bell Beaker phase (Late-Final Copper Age) (Ferrarese Ceruti 1976: 191). Most other tombs, including t.22, where skeletal materials were recovered, were assigned to the EBA1 based on association with cultural markers. The skeletal remains have been partially studied by F. Germanà (1995: 129 and references therein), but only a small fraction of the data was published, and a complete analysis to modern standards is still missing. Animal bone remains were also recovered but only a short preliminary report exists on a small assemblage from t. 16 [22].

Nine radiocarbon dates largely confirm the overall attribution of materials from t.16, but open the way for new interpretations of t.22: of the seven dates for t. 16, five were fully compatible with an EBA attribution (cumulative cal BP 2σ range between 4243 and 3894), while the other two (MAMS-38299, 3909 ± 19 BP = cal 4420-4260 BP 2σ, and MAMS-38300, 3880 ± 22 BP = 4411-4245 cal BP 2σ) appear early enough for the Bell Beaker phase, which culturally can be considered ancestral to Sardinian EBA.

The two samples from t.22 yielded one date that belongs to the Bronze Age, mostly within the initial MBA (MAMS-38302, 3421 ± 20 BP = 3808-3612 cal BP 2σ). The other one yielded an unexpectedly early date that places it fully within the Late Neolithic (MAMS-38301, 5042 ± 21 BP = 5894-5732 cal BP 2σ), showing that the commingled bone assemblage is the result of multiple phases of burial.

Site and Individual Descriptions from the Seulo Caves Project

Robin Skeates

For an introduction to the Seulo Caves see reference [23]**.**

SITE: Riparo sotto roccia Su Asedazzu

Cannisoni, Seulo, then Cagliari prov., now South Sardinia prov.

Lat: 39°51′22.10″ N Long: 9°14′56.68″ E

Excavation: 2014 (dir. Robin Skeates)

A small cave and rockshelter, used in successive phases of the Bronze Age as a human burial place and historically as a herder's shelter. (All body parts are represented, suggesting the primary burial of whole bodies and the later accumulation and dispersal of defleshed bones.)

**aDNA sample ID (small find #)**	**Archaeological context**	**Human bone type**	**C14 lab. code**	**C14 determination**	**Calibrated date-range BC (CALIB 7.10)**	**Period/ culture (after Tykot 1994)**	**Reference**
MA87 (134)	Surface find	Juvenile maxilla	SUERC-38110	2865±35 BP	1110–981 cal BC (68.3 %) 1187–923 cal BC (95.4 %)	Final Bronze Age (Nuragic III)	23,24 (Table S7)
SUA003 (307)	Context 1, Grid Square 9	Petrous portion of skull	MAMS-28656	2953±27 BP	1211- 1125 cal BC (68.3 %)<br><br>1257-1056 cal BC (95.4 %)	Late Bronze Age (Nuragic II)	This publication
SUA006 (103)	Context 5, Grid Sq. 9	2nd molar, extracted from juvenile mandible	MAMS-39539	2965±21 BP	1219-1128 cal BC (68.3 %)<br><br>1260-1116 cal BC (95.4 %)	Late Bronze Age (Nuragic II)	This publication
SUA001 (301)	Context 1, Grid Square 5	Petrous portion of skull	MAMS-28654	3060±28 BP	1388-1279 cal BC (68.3 %)<br><br>1409-1233 cal BC (95.4 %)	Middle Bronze Age (Nuragic I)	This publication
SUA007 (138)	Context 1, Grid Sq. 1	Upper canine	MAMS-39540	3597±22 BP	2010-1918 cal BC (68.3 %)<br><br>2022-1893 cal BC (95.4 %)	Early Bronze Age (Bonnanaro A)	This publication
MA78 (102)	Context 3, Grid Square 12	1st molar extracted from a child's mandible	MAMS-26901	3658±26 BP	2124–1977 cal BC (68.3%) 2134–1949 cal BC (95.4%)	Early Bronze Age (Bonnanaro A)	24 (Table S7)
SUA002 (303)	Context 2, Grid Square 3	Petrous portion of skull	MAMS-28655	3732±30 BP	2198-2048 cal BC (68.3%)<br><br>2205-2033 cal BC (95.4%)	Early Bronze Age (Bonnanaro A)	This publication

SUA005 (101)	Context 3, Grid Sq. 11	Premolar, extracted from mandible of older adult	MAMS-39538	3790±22 BP	2281-2150 cal BC (68.3%)<br><br>2290-2142 cal BC (95.4%)	Early Bronze Age (Bonnanaro A)	This publication
MA88 (136)	Context 5, Grid Square 9	2nd molar, extracted from the left portion of a female adult mandible	MAMS-26902	3794±34 BP	2286–2150 cal BC (68.3%)<br><br>2344–2062 cal BC (95.4%)	Early Bronze Age (Bonnanaro A)	24 (Table S7)

SITE: Riparo sotto roccia Su Cannisoni 1

Cannisoni, Seulo, then Cagliari prov., now South Sardinia prov.

Lat: 39 º 51′ 38.45″ N Long: 9 º 15′ 9.08″ E

Excavation: 2009 (dir. Robin Skeates)

A large rock-shelter with a Middle Bronze Age secondary burial deposit covered by a cairn, placed below a small natural spring. (One of the human vertebrae has been matched to another from the nearby burial cave of Sa Grutta ′e is Bittuleris, from where it was probably obtained.)

MA82/SC1004 (117)	Context 2, Grid Sq 5	Skull fragment	OxA-22194	3220±28 BP	1508-1449 cal BC (68.3 %)<br><br>1601-1426 cal BC (95.4 %)	Middle Bronze Age (Nuragic I)	23,24 (Table S7)
SCA003 (115)	Context 2, Grid Sq. 6	2nd lower premolar	MAMS-39 530	3281±28 BP	1610-1526 cal BC (68.3 %)<br><br>1622-1501 cal BC (95.4 %)	Middle Bronze Age (Nuragic I)	This publication

SITE: Riparo sotto roccia Su Cannisoni 2

Cannisoni, Seulo, then Cagliari prov., now South Sardinia prov.

Lat: 39 º 51′ 37.2492″ N Long: 9 º 15′ 12.276″ E

Survey: 2009 (dir. Robin Skeates)

A rock-shelter used in the Early Bronze Age as a human burial place.

MA81/SC1003 (116)	Surface find	Proximal left ulna	SUERC-381 11	3555±35 BP	1952-1783 cal BC (68.3 %)<br><br>2015-1771 cal BC (95.4 %)	Early Bronze Age (Nuragic I)	23,24 (Table S7)

SITE: Su Stampu Erdi

Tonnulù, Seulo, then Cagliari prov., now South Sardinia provinc.

Lat: 39 º 52′ N Long: 9 º 12′ E

Survey: 2009 (dir. Robin Skeates)

A cave complex with two entrances, corridors and speleothems, used in the Early and Middle phases of the Bronze Age as a human burial place.

No id # published	Surface find	Human bone	Beta-37705	3190±80 BP	1605-1323 cal BC (68.3 %)<br><br>1643-1262 cal BC (95.4 %)	Middle Bronze Age (Nuragic I)	25
MA85/ SE1011 (132)	Surface find	Tibia	SUERC-381 11	3579±27 BP	1956-1890 cal BC (68.3 %)<br><br>2023-1880 cal BC (95.4 %)	Early Bronze Age (Bonnanaro A)	23,24 (Table S7)

SITE: Sa Forada de Gastea / Grotta Gastea

Monte Gastea, Seulo, then Cagliari prov., now South Sardinia.

Lat: 39 º 51′ N Long: 9 º 12′ E

Survey: 2009 (dir. Robin Skeates)

A small cave used in the Early Bronze Age as a human burial place.

MA86/ MG1012 (133)	Surface find	Adult right fibula	OxA-22650	3647±29 BP	2114-1959 cal BC (68.3 %)<br><br>2134-1937 cal BC (95.4 %)	Early Bronze Age (Bonnanaro A)	23

SITE: Su Grutta ′e is Bittuleris / Sa Omu ‘e is Ossus

Cannisoni, Seulo, then Cagliari prov., now South Sardinia prov.

Lat: 39 º 51′ 38.46″ N Long: 0 º 15′ 9.36″ E

Excavation: 2009 (dir. Robin Skeates)

A small cave used in the Middle Bronze Age as a human burial place. (Osteological study of the human remains indicates successive primary inhumations of adults and children, males and females, and later disturbance and fragmentation of their bones.)

ISB002 (130)	Context 1, Grid Sq. 4	Upper 1st molar	MAMS-39523	3375±28 BP	1691-1629 cal BC (68.3 %)<br><br>1743-1615 cal BC (95.4 %)	Middle Bronze Age (Bonnanaro B)	This publication
(175)	Context 3, Grid Sq 4, Spit 2	Adult longbone	OxA-22193	3398±26 BP	1740-1661 cal BC (68.3 %)<br><br>1749-1629<br><br>cal BC (95.4 %)	Middle Bronze Age (Bonnanaro B)	[23]
ISB001 (1009)	Surface find	Petrous portion of skull	MAMS-28658	3460±29 BP	1873-1698 cal BC (68.3 %)<br><br>1880-1692 cal BC (95.4 %)	Middle Bronze Age (Bonnanaro B)	This publication
S1249 (711)	Context 1, Grid Square 4	2nd upper left premolar	NA	NA	NA	Middle Bronze Age (Bonnanaro B)	This publication (*aDNA by Haak/Reich)
S1250 (723) SuB7	Context 1, Grid Square 4	Lower incisor	NA	NA	NA	Middle Bronze Age (Bonnanaro B)	This publication (*aDNA by Haak/Reich)
S1252 (737)	Context 1, Grid Square 4	Tooth	NA	NA	NA	Middle Bronze Age (Bonnanaro B)	This publication (*aDNA by Haak/Reich)
S1253 (738)	Context 1, Grid Square 4	Tooth	NA	NA	NA	Middle Bronze Age (Bonnanaro B)	This publication (*aDNA by Haak/Reich)

SITE: Grutta I de Longu Fresu

Foresta di Addoli, Seulo, then Cagliari prov., now South Sardinia prov.

Lat: 39 º 51′ 6.25″ N Long: 9 º 16′ 13.32″ E

Excavation: 2009 (dir. Robin Skeates)

A small cave comprising a tunnel with lateral niches and former springs (plus a hole leading down to lower parallel tunnel), used in the Middle Neolithic as a cult cave, with human remains deposited at its innermost end. (Adult and child remains, probably originally deposited as primary burials then later disturbed.) A Middle Bronze Age mortuary phase is also indicated by a more recent radiocarbon determination on human bone.

LF1001	Surface find	Female left temporal with petrous	MAMS-28657	3509±28 BP	1885-1774 cal BC (68.3 %)<br><br>1910-1749 cal BC (95.4 %)	Middle Bronze Age (Bonnanaro B)	This publication (Posth)
MA79/ LF1002	Surface find in hole at back of cave	Juvenile female tibia	OxA-22195	5258±34 BP	4224-3991 cal BC (68.3 %)<br><br>4229-3981 cal BC (95.4 %)	Middle Neolithic (Bonu Ighinu)	[23,24 (Table S7)]
No id # published	Surface find	Adult skull	OxA-X-2236-44	5315±36 BP	4231-4059 cal BC (68.3 %)<br><br>4257-4042 cal BC (95.4 %)	Middle Neolithic (Bonu Ighinu)	[26]
No id # published	Context 2, Grid Sq 3, Spit 1	Adult skull	OxA-22196	5354±34 BP	4315-4074 cal BC (68.3 %)<br><br>4324-4053<br><br>cal BC (95.4 %)	Middle Neolithic (Bonu Ighinu)	[23]
LON004 (112)	Context 5, Grid Sq. 4	Upper canine	MAMS-39525	5375±33 BP	4325-4173 cal BC (68.3 %)<br><br>4331-4067 cal BC (95.4 %)	Middle Neolithic (Bonu Ighinu)	This publication
LON003 (111)	Context 1, Grid Sq. 3	Upper canine	MAMS-39524	5481±32 BP	4356-4272 cal BC (68.3 %)<br><br>4438-4260 cal BC (95.4 %)	Middle Neolithic (Bonu Ighinu)	This publication

Description of Neolithic Sites Noeddale e S'Isterridolzu

Vittorio Mazzarello

SITE: Noeddale

The archaeological site is located on Ossi municipality (Sassari). It consists of a necropolis of six tombs - domus de janas type - limestone-carved in a saddle that slopes towards the valley of Sae (lat: 40°39′59″ N, long: 8°35′34″ E). Mazzarello et al.[27] studied the skeletal remains and the preliminary osteological analysis of chamber B and chamber D enabled the identification of at least 63 individuals of all ages. The site is assigned to the Ozieri culture (Middle-Late Neolithic). Tomb III was excavated by A Moravetti in 1986 and it yielded several bone remains. Two individuals analysed in this study originated from this tomb: Noe4 (NOE001) radiocarbon dated to 5306 ± 32 BP = 2220-2035 calBC and Noe5 (NOE002) radiocarbon dated to 5352 ± 32 BP = 2304-2036 calBC.

SITE: S'Isterridolzu

The site is also located on the territory of Ossi municipality (Sassari). The necropolis consists of six domus de janas tombs excavated in low limestone outcrops distributed within a vast flat land area of about 10,000 square meters (lat: 40°37'34"N, long: 8°38'8"E). The distance between the hypogea varies from 10 m to about 200 m. Tomb IV, excavated by Ferrarese Cerruti in 1971, has returned bone remains and pottery with one fragment belonging to the Bell Beaker culture and the rest to the Bonnanaro phase [28,29]. Germanà in [30] performed osteological analysis of the cranial elements while other human remains underwent a preliminary analysis by the University of Sassari. From Tomb IV originated three samples included in this work: SI7 (SID005) radiocarbon dated to 5316 ± 31 BP = 2223-2028 calBC, SI8 (SID006) radiocarbon dated to 4116 ± 28 BP = 2847-2560 calBC and SI5 (SID003), on which radiocarbon dating was not successful.

Description of Post-Nuragic Sites

SITE: Villamar
Elisa Pompianu, Clizia Murgia, Noreen Tuross, Peter Van Dommelen

Villamar is a modern town of medieval origins situated in the gently rolling fertile hills of the Marmilla district in southern central Sardinia. While there is some evidence for prehistoric and later settlement in the area of the post-medieval town center, the only archaeological remains that have been systematically recovered and properly documented are Punic in date, and come from a nearby ancient cemetery. Following preliminary work in the 1990s[31], new excavations began in 2013[32], and have since resulted in the investigation of 24 tombs.

The cemetery comprises a wide variety of tombs that range from simple trenches in the sandstone and below marble rock to complex chamber tombs made up of one or rarely two rooms and a shaft entryway. Most of these tombs, including many of the trenches, hold multiple burials, but it is the larger rock-cut chamber and shaft graves that have consistently contained multiple burials. Overall, the tombs investigated so far have been dated between the mid-4th and the end of the 3rd / early 2nd century BCE on the basis of the pottery present in the graves. It is however frequently impossible to make reliable associations between bodies and grave goods because of the reuse of the tombs. The size of the cemetery is unknown, as it appears to continue in an archaeological area of ~2,300 mq, and even under houses of the current town[32].

In all, twelve human skeletal elements were screened for aDNA and six yielded usable aDNA. All sampled bones come from the same tomb (#T.16) with a minimum number of 28 individuals, including two cremated skeletons. The latter were the last deposited in the chamber and the ceramic container used to contain the burnt remains suggests it was the last time the tomb was accessed (late 3nd - early 2nd century BCE). It is therefore very likely that the human remains inside the burial

chamber were disarticulated in antiquity because of the repeated use of the grave. As a result, the reconstruction of complete skeletons is limited. Among the burials, we report the unusual presence of 18 sub-adults, of whom eight died at fetal age (some potentially at birth), and eight died at early infant age (Infant I)[33]. Initial radiocarbon dating of the skeletal material yielded ages ranging from 800 to 400 BCE. Because this range in radiocarbon ages is somewhat greater and earlier than anticipated based on pottery associations and on the tomb typology, additional testing is currently underway.

Site: Monte Sirai
Michele Guirguis, Rosana Pla Orquin, Noreen Tuross, Peter Van Dommelen

This site is located in the far south-west of Sardinia on a flat-topped basalt plateau (190 meters above sea level) about half a dozen km from the actual coast. It has commanding views over the town and lagoon of Sant'Antioco, where *Sulky,* the earliest Phoenician settlement of Sardinia was established in the early 8th century BCE [34]; it also controls access to the rich mineral depositions in the inland area of the Sulcis-Iglesiente district[35–37]. The site is comprised of three large sectors: the main sector of the settlement, the "acropolis", on the southern portion of the hill; the sacred place, or *tofet*, in the northern portion of the site, and the large cemetery located in the valley separating the acropolis from the *tofet*. The first excavations of the site were carried out in the 1960s, and again in the 1980s, though systematic excavations, involving a large area, began in 2005 and are still on-going. Archaeological evidence indicates that Monte Sirai was founded by Phoenicians and the local Nuragic people at the end of the 8th-early 7th century BC, and was completely abandoned during the 1st century BC[37,38].

Recent excavations in the cemeteries beyond the settlement area on the plateau, carried out since the 1980s under the direction of Piero Bartoloni, have demonstrated that the cemetery was used between the end of the 7th century until the middle of the 4th century BC with a large variability in mortuary practices[39–41]. It includes graves of single and multiple individuals, primary cremation burials, *enchytrismoi* (transport amphorae as containers of child burials) and "semi-combustions"[42–44].

The samples that have been analysed for this study all come from the late Phoenician to early Punic cemetery [45], where three distinct individuals have been sampled for aDNA. Only two, however, have provided usable results. Multiple radiocarbon dates from skeletal material range from 800-550 BCE but the flat section of the calibration curve for the latter centuries (the so-called Hallstatt plateau) precludes more precise dating by means of radiocarbon decay. The site has been previously investigated through aDNA analysis of the mtDNA from ten individuals revealing both a certain level of maternal continuity with earlier Sardinian populations and the arrival on the island of non-local mtDNA haplogroups [46].

SITE: Duomo of San Nicola, Sassari (external necropolis)
Daniela Rovina, Raffaella Bianucci, Rita Maria Serra, Pasquale Bandiera

Between 2002 and 2006, two excavation campaigns carried out on the square surrounding the Duomo of San Nicola in Sassari lead to the identification of a vast cemeterial area, historically and archeologically dated between the 13th and the 18th centuries[47].

In 1277 AD, Bishop Dorgotorio subdivided the city in different parishes; the area surrounding San Nicola's Church was the only urban cemetery of that time.

The 2002 excavation focused on a 70 square meters area located on the external southern perimeter of the square facing the church. Twelve juxtaposed stone graves (2.20 m length, 8.5 m width and 1 m deep, W/E oriented) containing one to two individuals in primary deposition were uncovered in some cases on top of secondary burials. These stone graves covered by stone slab correspond to the oldest nucleus of the external cemetery (13th to 14th century used until the 15th century).

The northern side of a quadrangular stone basin (measuring ca. 5 m per side) was also identified in the external southern perimeter. Archeologically dated to the Contemporary Period, it was possibly used as a fire-water storage basin during WWII. The stone basin partially or completely destroyed the graves dating to earlier periods.

A second cemeterial area, archaeologically dated between the 16th and the 18th centuries, was identified in 2006 on the western side of the square between the Duomo facade and the *Marianum.* This second area, which was overlying the late medieval cemetery, contained a series of superficial ground graves with ellipsoidal form that were carefully W/E oriented. The graves were covered with rocks and sediment and did not show evidence of any archaeological marker. The skeletal remains, representing both primary and secondary burials, were laid on a supine position with the arms crossed on the chest or on the abdomen. All graves faced the church.

Forty-two individuals were exhumed in total with the average age at death of 38 years. Sub-adults and adolescents represented 21% of the skeletons (average age at death 12 and a half years). Among the adults, 38% were male (mortality peak 31-45 years) and 24% were female (mortality peak 15-30 years).

All samples genetically analysed derive from the 2002 exaction archeologically dated to the 14th to 15th century. SNN004 was radiocarbon dated to the 13th century and it probably represents an earlier secondary burial, however it should be subsequent to 1277, which is the year when the cemetery was founded. The radiocarbon date from SNN002 instead spans between the 15th and the 17th century but the retrieved grave clothes were more common during the 14th-15th century while extremely rare afterwards.

SITE: Monte Carru (Alghero)

Alessandra La Fragola, Daniela Rovina, Raffaella Bianucci, Rita Maria Serra, Pasquale Bandiera

Monte Carru is a mountain located two kilometres northeast of Alghero (Sassari) and it extends from the seashore up to 90 masl facing a large fertile plain. Thanks to its peculiar position - which allowed both territory control and environmental exploitation - this area was densely inhabited since the Nuragic Period as demonstrated by the presence of two *nuraghi*, located one km apart on the top of Monte Carru[48–51].

Rescue excavations carried out in 2007 brought to light an important cemeterial area associated with a Roman Period urban centre. Archaeological evidence corroborate the hypothesis that the site corresponds to the lost city of *Carbia*, which is mentioned in ancient texts but never identified. The urban centre was located in the southwestern plain that surrounds Monte Carru whereas the funerary area was placed at the bottom of the mountain on its southwestern slope. The large extension of the necropolis implies that the urban centre played a relevant role in the Roman society of the time.

The necropolis is archaeologically dated to the Roman Imperial Period (1st-3rd century AD) beside some pottery findings dated to the 1st century BC but that are not associated to the human burials. Different types of 1st-3rd century AD burials have been identified such as incinerated, ground burials and, more frequently, rock burials.

In total 350 burials were uncovered at this site and of those 181 were incinerated and located in the eastern plain or along Monte Carru's inclined northern rocky slope. At the moment, only 60 individuals from 53 burials underwent anthropological investigations. Of those, 9% are subadults and 91% adults divided in 47% males (age of death 25-55 years), 36% females (age of death 25-45 years) and 8% of undetermined sex with a low frequency of pathologies.

In the lowest western area 104 graves were identified. Here, the softer ground facilitated pits to be dug. Few tombs were found empty whereas others had been plundered in antiquity.

The archaeological record indicates that the necropolis was mainly occupied during the 1st century AD when the deceased were generally incinerated. The practice of inhumation started to develop as a rule from the 2nd century AD onward. In some cases the corpses were laid directly in the pit whereas, in others, they were wrapped in shrouds or bandages. Different typologies of graves ("*alla cappuccina*" burials, jar burials, coffered burials, ground burials, rock burials) have been identified.

The burials were predominantly single graves with the only one burial that contained the skeletal remains of three individuals. In the southern and western sides of the necropolis superimposing layers of burials were also identified.

The bodies were mainly buried in a supine position with the arms along the sides, crossed on the chest or on the pelvis. In some cases, the corpses had been deposed on one side or with one or both lower limbs folded. The orientations of the skeletons were prevalently in an E/W direction but they were often adapted to the morphology of the ground. The funerary goods associated to the 2nd to 3rd century AD burials were generally poorer than that ones dated to the 1st century AD where often no grave goods were found.

Supplementary Note 2: Validating quality and contamination of aDNA

Joseph H. Marcus, Kushal Dey, Hussein Al-asadi, Harald Ringbauer, Cosimo Posth

Postmortem Damage Filtering

Individuals with high levels of mtDNA or X chromosome based contamination estimates were removed in our main analysis. However, population genetic analyses could still be affected by more subtle modern contamination. To assess this possibility, we filter out reads that do not show a signature of post-mortem damage (pmd), as reads that carry a damage signature are less likely to be introduced by modern contamination[52]. We used `pmdtools` (`https://github.com/pontussk/PMDtools`) to compute a likelihood-based damage score for each read and subsequently removed reads which showed little evidence of being damaged. We then generated pseudo-haploid genotype calls on these "pmd-filtered" individuals and projected them onto the PCs computed in the modern west Eurasian and north African individuals from the Human Origins dataset, as described in the Materials and Methods. We corrected for the regression towards the mean effect in high-dimensional PCA using a simple jackknife estimator (see Supp. Info. 7).

We found that all the samples we analyzed in the main results and that have enough covered SNPs after pmd filtering show little difference between the pmd-filtered and corresponding unfiltered PC scores (Fig. 2). This observation supports our sample filtering criteria, and as such our population genetic analyses are unlikely to be strongly affected by contamination. A few individuals had too few covered SNPs after pmd filtering to make accurate predictions of their ancestry, which possibly explains larger observed differences between the pmd-filtered and the original projection.

aRchaic

We estimate DNA damage profiles using `aRchaic`[53]. In the `aRchaic` model, mismatches are counted across all of an individual's sequence reads with corresponding measured features, including mismatch type, mismatch position on the read, flanking reference nucleotides, and strand orientation. Each mismatch is modeled as originating from a mixture of K profiles defined by these features and adaptively learned during inference via an EM algorithm. Maximum-likelihood estimates of mixture proportions for each individual are then displayed on a stacked bar chart where each row is a different sample and each colored bar represents the proportion of the ith individual's mismatches coming from the kth mismatch profile 3. We applied `aRchaic` to the ancient Sardinia data for $K = 2$, $K = 3$, and $K = 4$. In (Fig. 3, Fig. 4, Fig. 5), we observe the typical pattern representative of ancient DNA, an enrichment of cytosine to thymine mismatches occurring preferentially at the ends of the read[54,55]. These observations helps to authenticate our data as being truly ancient. Ancient and modern individuals show distinctive mismatch profiles and as we increase $K = 2$ to $K = 4$ finer resolution substructure is revealed between subgroups of individuals. Specifically, samples treated with a protocol,

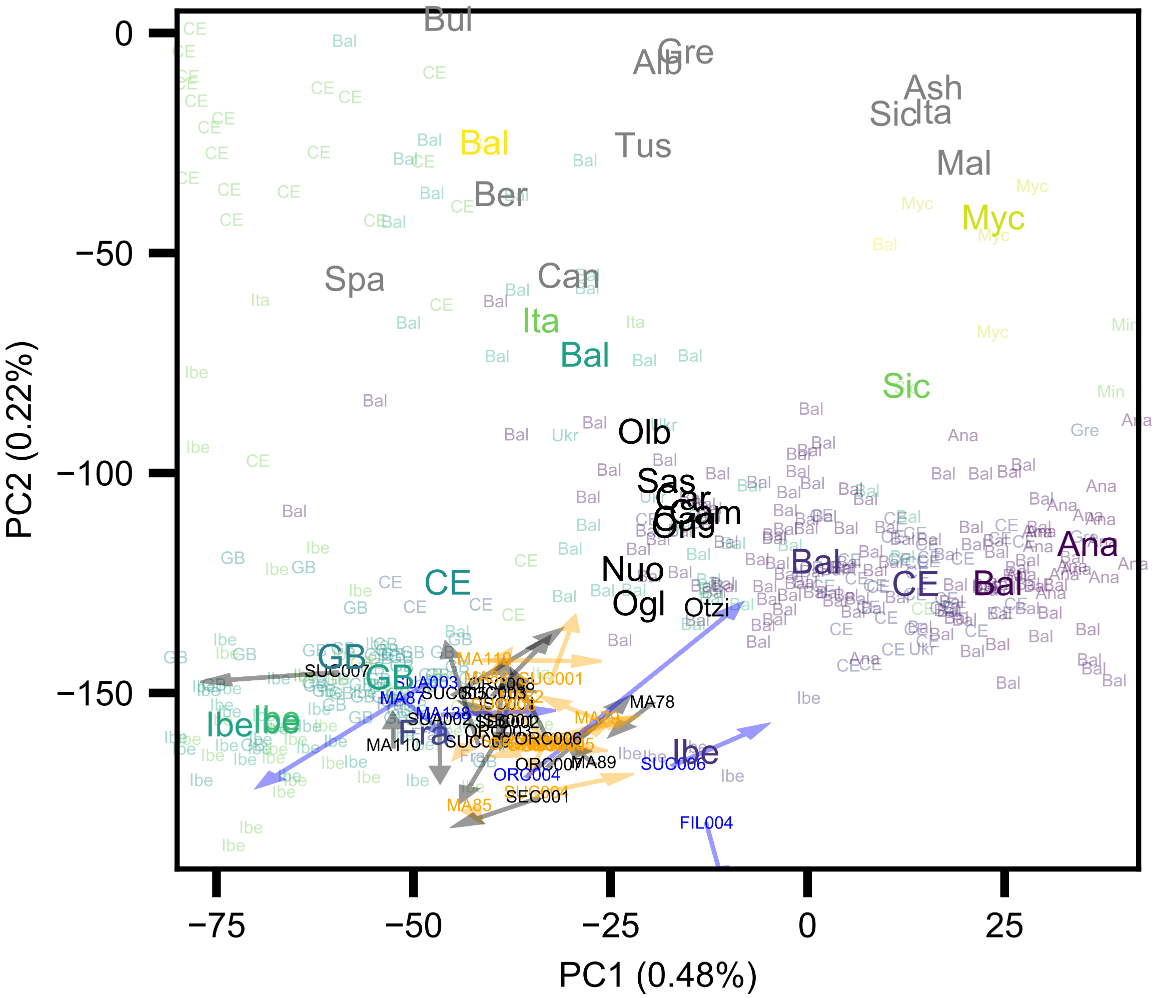

Supplementary Figure 2: Impact of PMD filtering on PCA projections of ancient individuals. The figure shows a visualization of PC1 and PC2 computed on modern individuals and projecting ancient individuals from our study, alongside ancients from previously published literature. Each arrow represents an ancient Sardinian individual, where the head of the arrow is the "pmd-filtered" projection and the tail is the "non-pmd-filtered" projection. We color each arrow given the following criteria: black for male individuals that had low X-based contamination estimates (<=0.05); orange for male individuals with high X-based contamination estimates (>0.05) or females; and blue for any remaining individuals with less than 35 thousand covered SNPs after PMD filtering.

UDGhalf treatment, to partially remove some of this damage signature from each sample, cluster distinctly from both untreated ancient and modern individuals. The modern individuals we included are a sub-sample of 43 low-coverage whole genome sequences from the 1000 Genomes Project Phase3 (PUR, FIN, CHS, GBR, CDX, MXL) and 7 deeply sequenced individuals from the Simons Genome Diversity project. The modern individu-

als show membership in two damage profile clusters. In previous analyses[53], such results have been found to arise from differing sample preparation protocols, and other plausible factors (for example differences in initial DNA quality). Importantly, none of the ancient samples show membership in the damage profile clusters found in the modern samples. This result is concordant with the low contamination estimates obtained for these samples (note: the samples analyzed with `aRchaic` here all passed our initial contamination rate filters based on mtDNA and nuclear contamination estimates, see Materials and Methods).

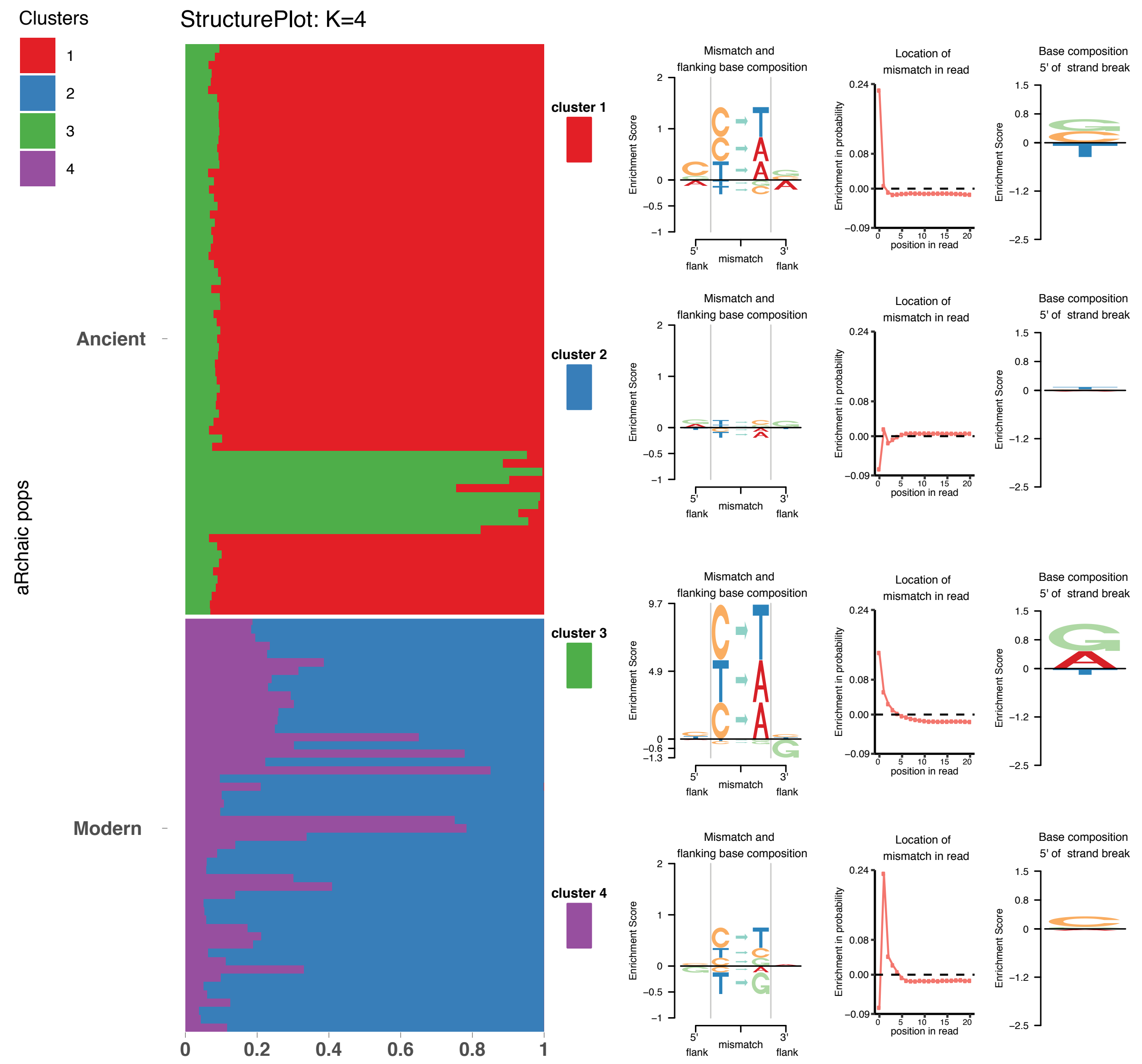

Supplementary Figure 3: Results of the package `aRchaic` for clustering mismatch profiles in sequence read libraries for (K=4). On the left we plot a stacked bar-chart where each row represents a `bam` file and the colored portions of each bar represent the mixture proportion for a given cluster. As we can see each `bam` file's mixture proportions must be non-negative and sum to one. On the right we display representations of the inferred latent variables that define each cluster. The left most plot displays the enrichment or depletion of different mismatch types, the middle plot displays the enrichment probability of observing a mismatch at a particular position along the read, and finally the right hand plot displays an enrichment score for the mismatch type observed at a strand break on the 5′ end of the fragment. All together these plots visualize both how the `bam` files are loaded on to each cluster as well as what defines each cluster.

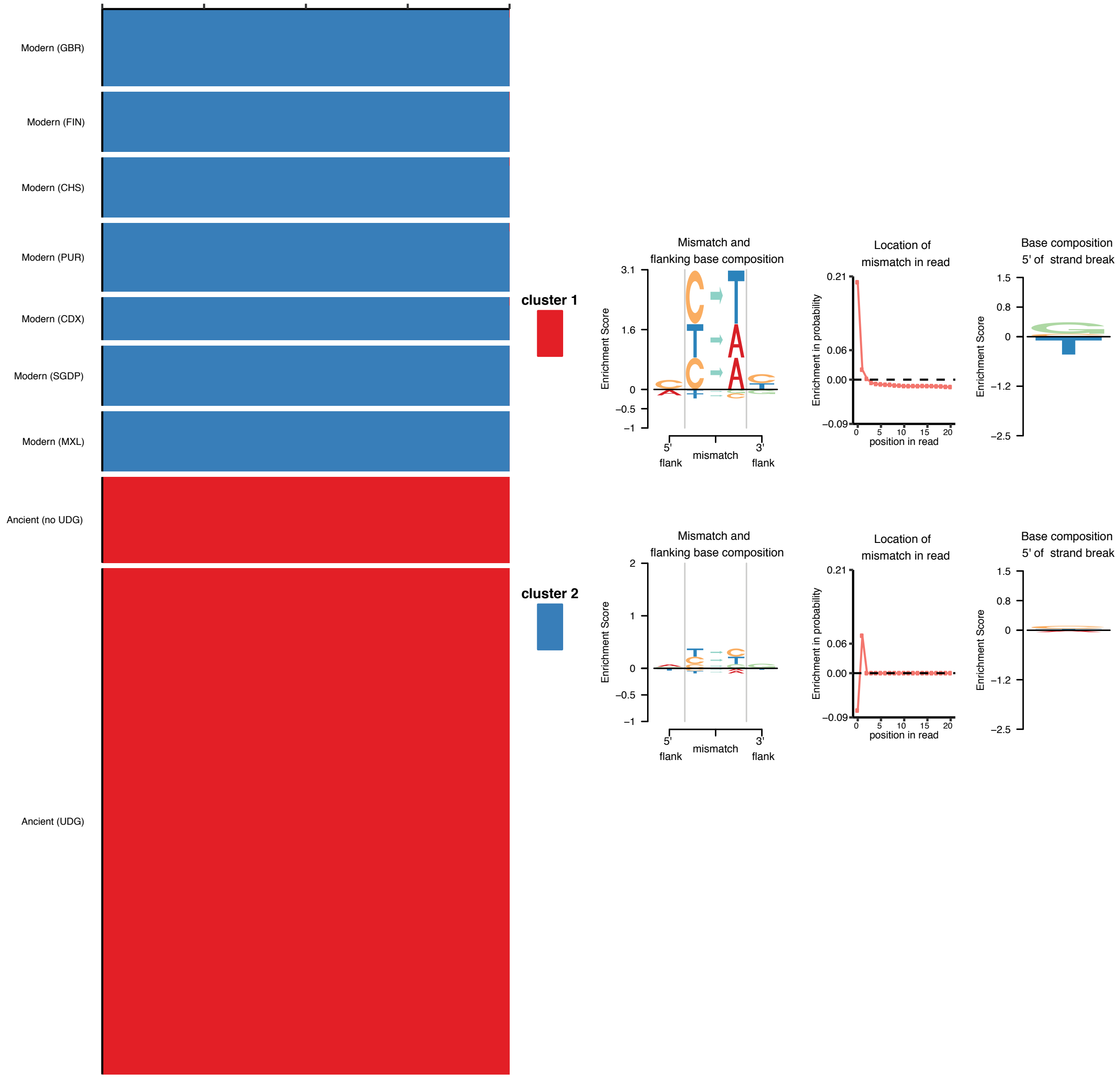

Supplementary Figure 4

Results of the package `aRchaic` (K=2) for clustering mismatch profiles in sequence read libraries. See (Fig. 3) for a detailed description of the panels.

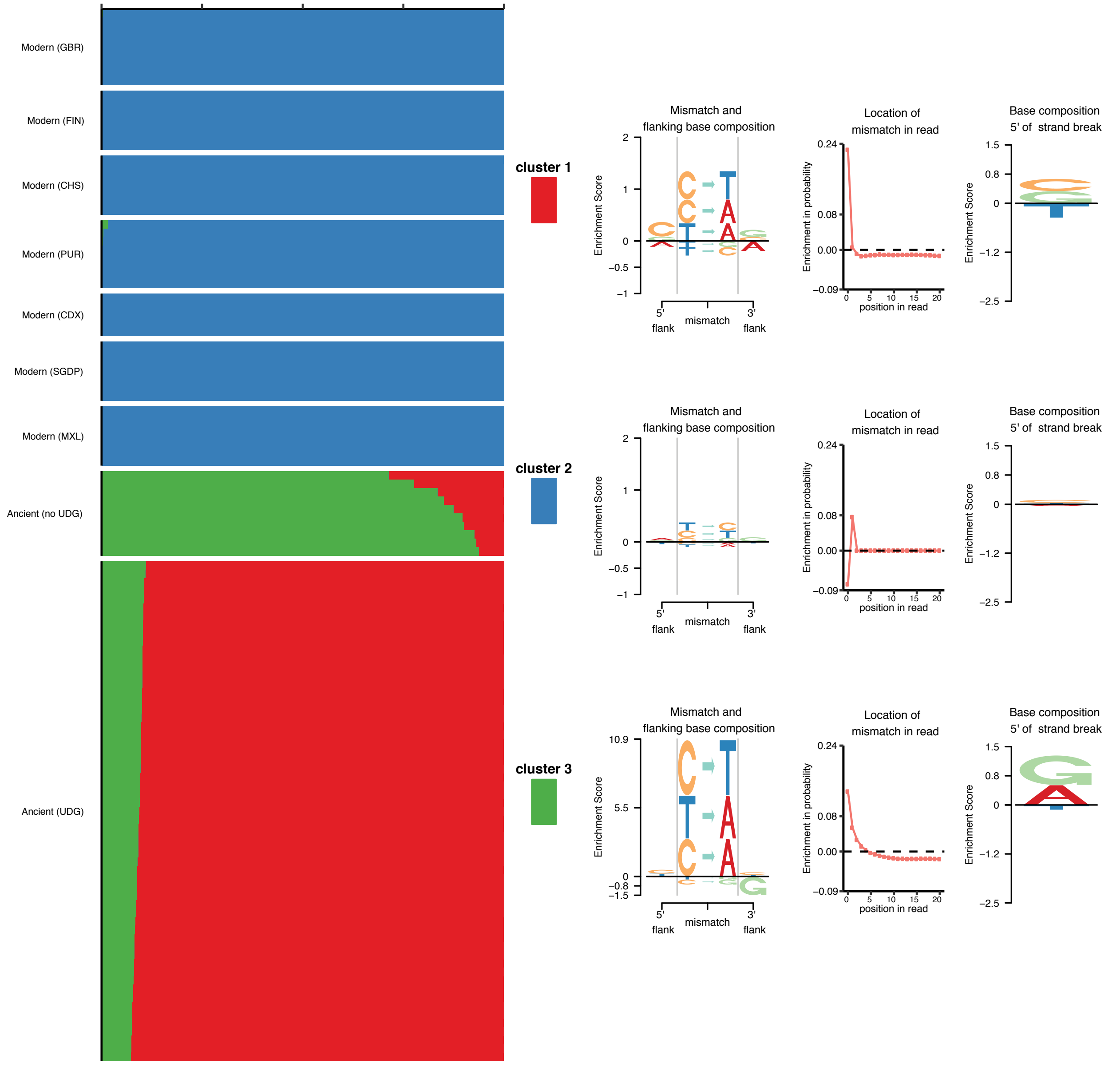

Supplementary Figure 5
Results of the package `aRchaic` (K=3) for clustering mismatch profiles in sequence read libraries. See (Fig. 3) for a detailed description of the panels.

Supplementary Note 3: Analysis of ancient Y haplogroups

Harald Ringbauer

We were able to assign Y haplogroup designations for 39 ancient Sardinian males (Sup. Data 1A and B, Materials and Methods).

Comparison to present-day Sardinia Y haplogroup variation

Supp. Fig. 6 presents an overview of the results. Some caution is required in interpretation as we observed substantial clustering of haplogroups within sample site (Sup. Data 1B) suggesting some sites may not be random samples of the population. That said, overall there is a notable shift from pre-Iron Age to present-day Sardinia. The "Sardinian" haplogroup I2-M26, carried by about 35% present-day Sardinian males and rare elsewhere[56], is found in only one ancient Sardinian sample from the early Middle Bronze Age and one individual from a Medieval site. The R1b-M269 haplogroup, dominant in present-day continental Iberia after having arrived there with the Bronze Age Indo-European expansion[57] and at 15% frequency in modern Sardinians[56], is absent throughout our transect from the Middle Neolithic to Nuragic in Sardinia, but later appears in three post-Nuragic ancient individuals. Similarly, the haplogroup E-M215, a major present-day Sardinian haplogroup (10%) that is prevalent in present-day Northern Africa, was not identified among any of the pre-Iron Age ancient individuals we sampled. However, we also detected signals of partial continuity from pre-Iron Age Sardinia. The Y haplogroups G2-L91 and R1b-V88, which were previously identified to have major Sardinia-specific sub-clades based on present-day Sardinian variation[56], were all identified in at least one ancient Sardinian male (Fig. 6).

R1b haplogroups in ancient Sardinians

The 1240k read capture data allowed us to call several R1b subclades in ancient individuals (see Supp. Fig. 7 for overview of available markers). While R1b-M269 was absent from our sample of ancient Sardinians until the Nuragic period, we detected R1b-V88 equivalent markers in 11 out of 30 ancient Sardinian males from the Middle Neolithic to the Nuragic with Y haplogroup calls. Two ancient individuals carried derived markers of the clade R, but we could not identify more refined subclades due to their very low coverage (Supp. Data 1B). The ancient geographic distribution of R1b-V88 haplogroups is particularly concentrated in the Seulo caves sites and the South of the island (Supp. Data 1B).

At present, R1b-V88 is prevalent in central Africans, at low frequency in present-day Sardinians, and extremely rare in the rest of Europe[58]. By inspecting our reference panel of western Eurasian ancient individuals, we identified R1b-V88 markers in 10 mainland European ancient samples (Fig. 8), all dating to before the Steppe expansion ($>$ 3k years BCE). Two very basal R1b-V88 (with several markers still in the ancestral state) appear in Serbian HGs as old as 9,000 BCE (Fig. 9), which supports a Mesolithic origin of the R1b-V88 clade in or near this broad region. The haplotype appears to have become associated with the Mediterranean Neolithic expansion - as it is absent in early and middle Neolithic

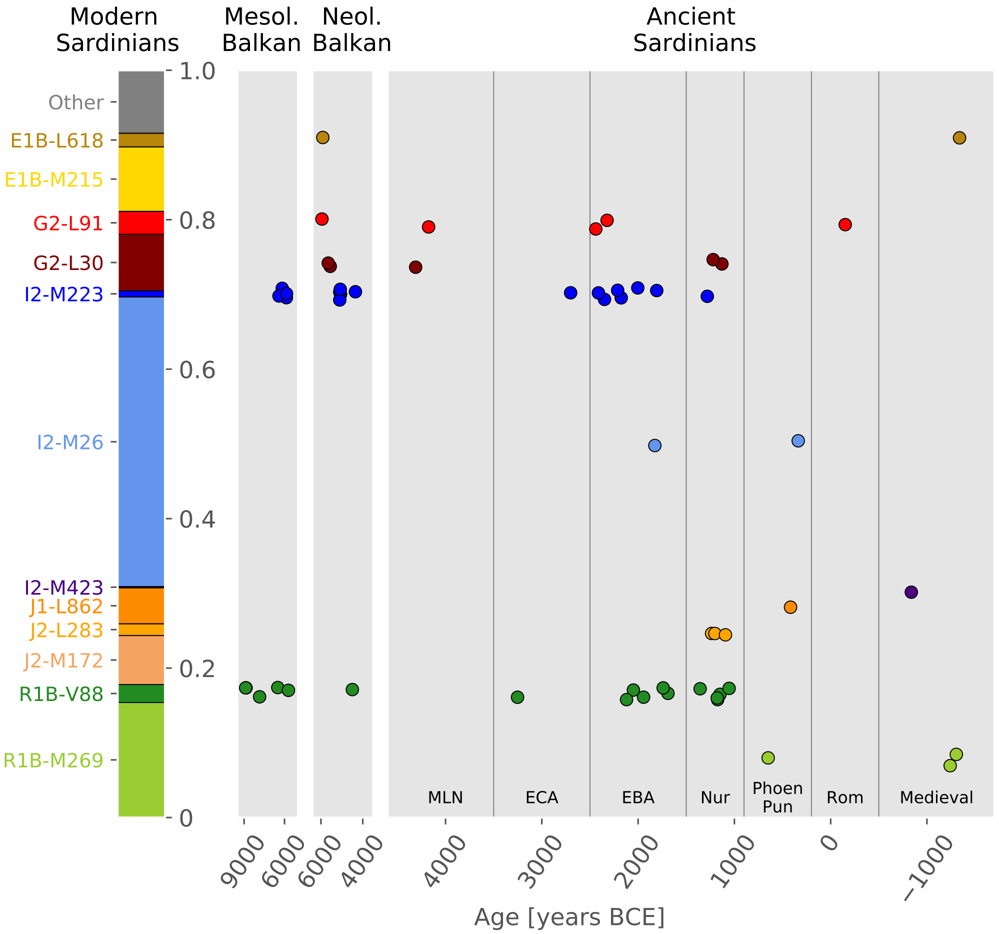

Supplementary Figure 6: A summary of Y chromosome variation in ancient Sardinians. We plot a stacked bar-chart representing all major present-day haplogroups, as well as all haplogroups found in at least one ancient Sardinian. The relative frequencies are based on haplogroup abundances in 1,204 present-day Sardinian males[56]. In the grey panels, each point corresponds to an ancient sample of a particular age (x-axis) and Y haplotype (y-axis, haplogroup denoted by alignment with modern haplogroups on the left, with jitter added to avoid overlap among individuals of the same age / haplotype configuration). The ancient Balkan data originate from the ancient reference panel (we include ancient individuals from Hungary in the broader Balkan definition).

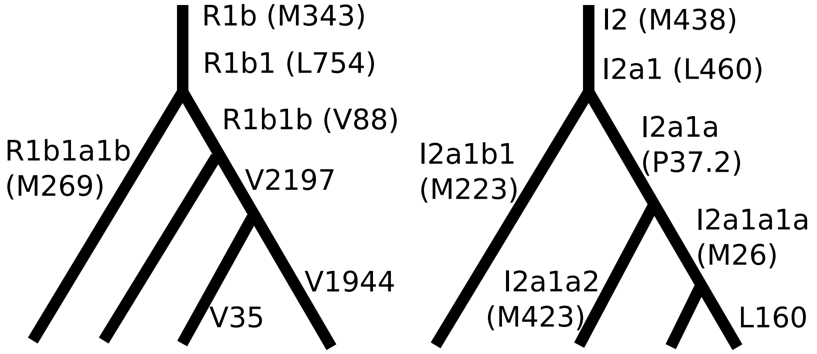

Branch	Equiv. Marker	# of Markers (1240k)	Present-day Distribution
R1b	M343	13	West Eurasia, North Africa
R1b1	L754	15	West Eurasia, North Africa
R1b1a1b	M269	76	Western Europe
R1b1b	V88	37	Sahel Zone, Sardinia
R1b1b2a	V2197	10	Sahel Zone, Sardinia
R1b1b2a1	V35	11	Sardinia
R1b1b2a2a	V1944	10	Sahel Zone
I2	M438	56	West Eurasia
I2a1	L460	3	West Eurasia
I2a1b1	M223	90	Mesolithic, Neolithic Europe
I2a1a2	M423	121	Neolithic Europe
I2a1a1a	M26	5	Sardinia
I2a1a1a1a1	L160	38	Sardinia

Supplementary Figure 7: Topology of relevant R1b and I2 subclades annotated with markers on the 1240k target For each subclade, the table shows one defining marker (ISOGG 2018), the number of equivalent markers that intersect the 1240k capture panel, as well as its broad past or present-day geographic distribution.

central Europe, but found in an individual buried at the Els Trocs site in the Pyrenees (modern Aragon, Spain), dated 5,178-5,066 BCE[59] and in eleven ancient Sardinians of our sample. Interestingly, markers of the R1b-V88 subclade R1b-V2197, which is at present-day found in Sardinians and most African R1b-V88 carriers, are derived only in the Els Trocs individual and two ancient Sardinian individuals (MA89, 3,370-3,110 BCE, MA110 1,220-1,050 BCE) (Fig. 9). MA110 additionally carries derived markers of the R1b-V2197 subclade R1b-V35, which is at present-day almost exclusively found in Sardinians[58].

This configuration suggests that the V88 branch first appeared in eastern Europe, mixed into Early European farmer individuals (after putatively sex-biased admixture[60]), and then spread with EEF to the western Mediterranean. Individuals carrying an apparently basal V88 haplotype in Mesolithic Balkans and across Neolithic Europe provide evidence against a previously suggested central-west African origin of V88[61]. A west Eurasian R1b-V88 origin is further supported by a recent phylogenetic analysis that puts modern Sardinian carrier haplotypes basal to the African R1b-V88 haplotypes[58]. The putative coalescence times between the Sardinian and African branches inferred there fall into the Neolithic Subpluvial ("green Sahara", about 7,000 to 3,000 years BCE). Previous observations of autosomal traces of Holocene admixture with Eurasians for several

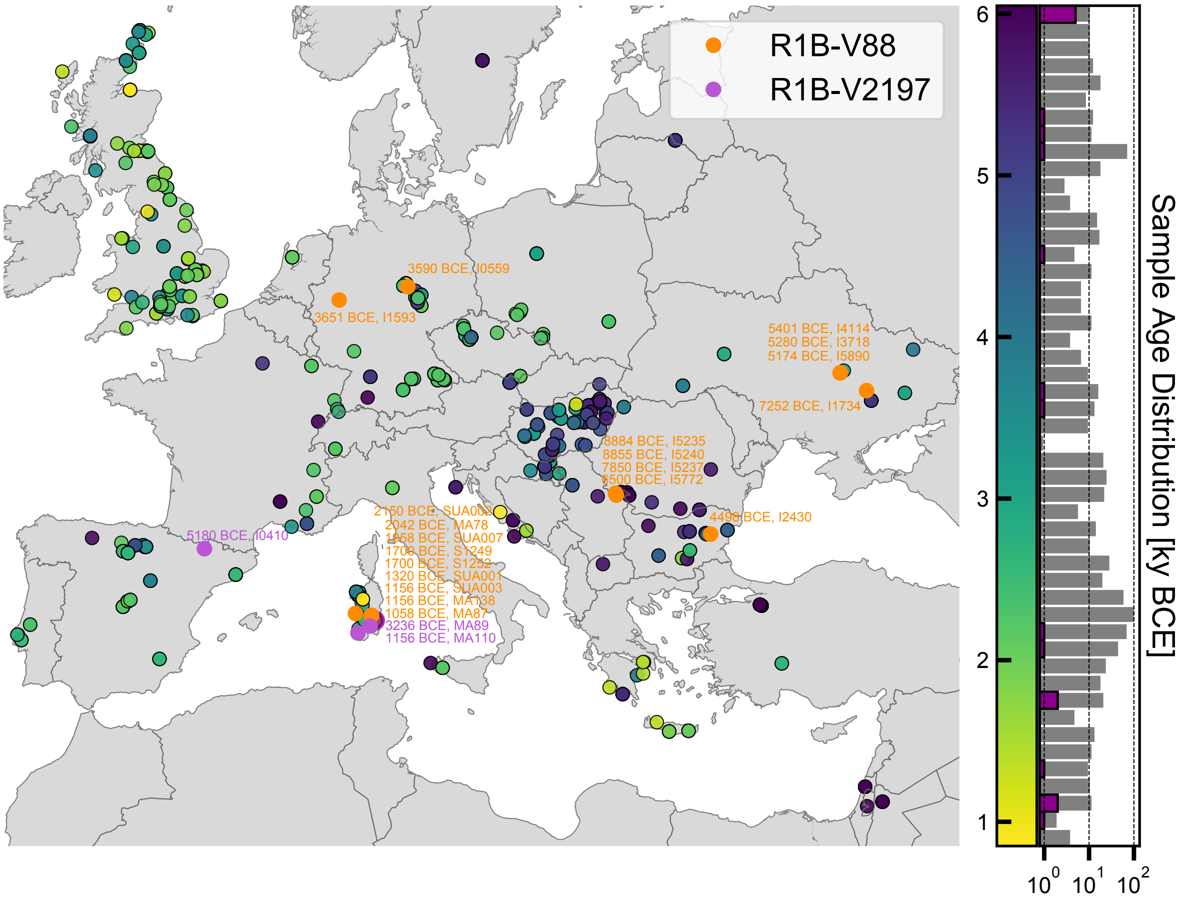

Supplementary Figure 8: Geographic and temporal distribution of R1b-V88 Y-haplotypes in ancient European samples. We plot the geographic position of all ancient samples inferred to carry R1b-V88 equivalent markers. Dates are given as years BCE (means of calibrated 2σ radio-carbon dates). Multiple V88 individuals with similar geographic positions are vertically stacked. We additionally color-code the status of the R1b-V88 subclade R1b-V2197, which is found in most present-day African R1b-V88 carriers.

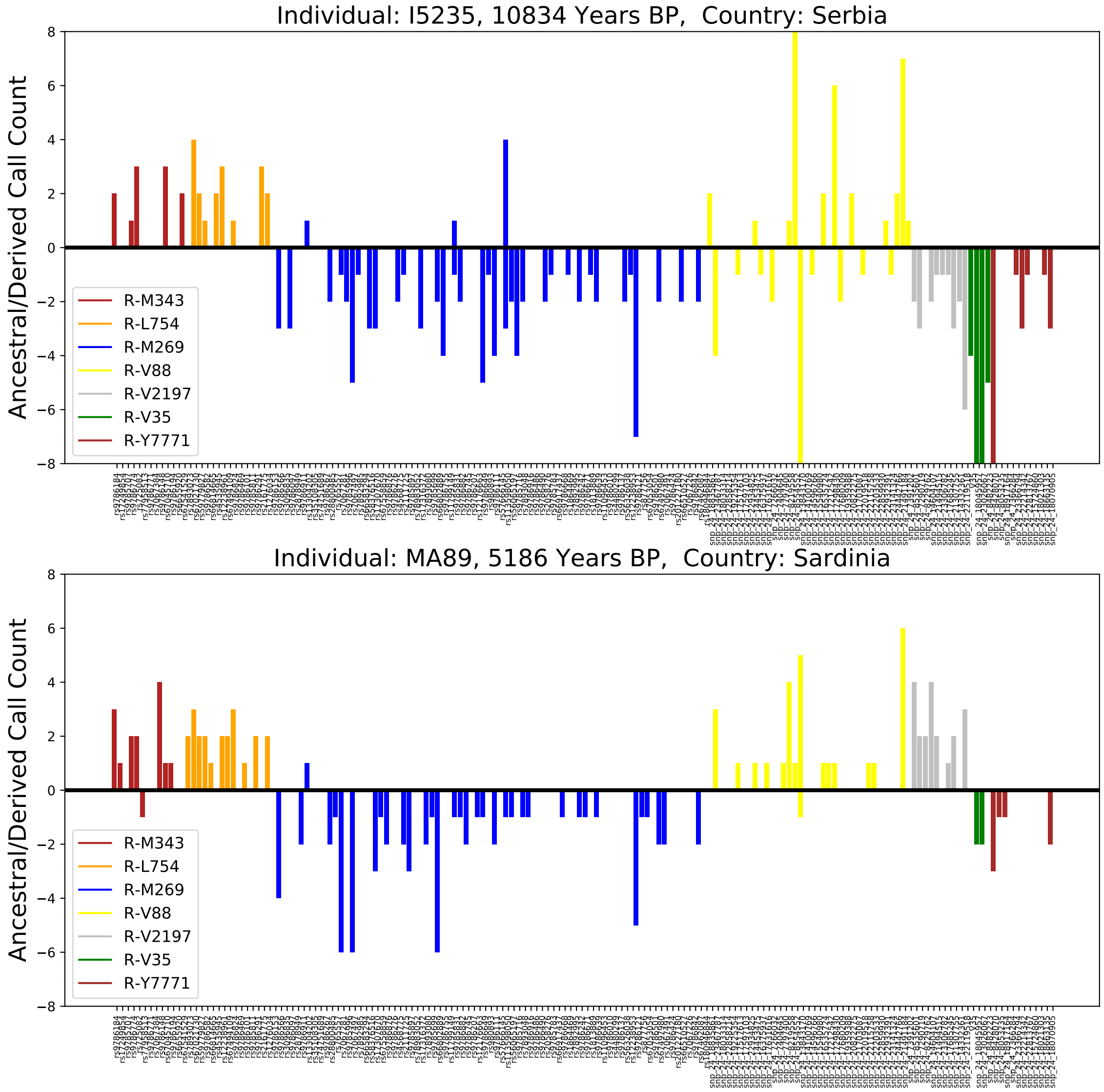

Supplementary Figure 9: Read data summary for R haplogroup mutations of a Copper Age Sardinian (bottom panel) and a HG individual from Serbia (top panel). The figure depicts for each marker along the x-axis, the number of calls for the ancestral (downwards) as well as derived alleles (upwards). Both individuals carry reads for the R1b-M269 markers in the ancestral state and for several derived V88 markers. The almost 11-thousand-year-old sample also has several of the equivalent R1b-V88 markers in the ancestral state, and this particular Sardinian has derived markers for the V88 subclade R1b-2197 (similar to the Neolithic Els Trocs sample from the Pyrenees).

Chadic populations[62] provide further support for a speculative hypothesis that at least some amounts of EEF ancestry crossed the Sahara southwards. Genetic analysis of Neolithic human remains in the Sahara from the Neolithic Subpluvial would provide key insights into the timing and specific route of R1b-V88 into Africa - and whether this haplogroup was associated with a maritime wave of Cardial Neolithic along Western Mediterranean coasts[63] and subsequent movement across the Sahara[58,64].

Overall, our analysis provides evidence that R1b-V88 traces back to eastern European Mesolithic hunter gatherers and later spread with the Neolithic expansion into Iberia and Sardinia. These results emphasize that the geographic history of a Y-chromosome haplotype can be complex, and modern day spatial distributions need not reflect the initial spread.

Supplementary Note 4: Pairwise similarity statistics

Harald Ringbauer, Joseph H. Marcus

Measures of pairwise genetic differentiation.

Supp. Fig. 10 and Supp. Fig. 11 depict the matrix of genetic similarities calculated using f_3-outgroup statistics and F_{ST} as described in the main text (Materials and Methods). Numerical values are reported in Supp. Data 2A and B.

Pairwise Relatedness.

To identify close relatives within our dataset of ancient Sardinians, we directly assessed pairwise relatedness. We first filtered to markers that have calls in at least 20 of the ancient Sardinian individuals, and within those for markers with (pseudo-haploid) minor allele frequency (MAF) > 0.2. For the resulting set of $n = 351,967$ markers, we calculated pairwise correlation of allelic state (relatedness) between individuals i and j:

$$f(i,j) = \frac{(p_i - \bar{p}) \cdot (p_j - \bar{p})}{\bar{p} \cdot (1 - \bar{p})}, \tag{1}$$

averaged over all markers with available (pseudo-haploid) calls for both individuals. Mean allele frequencies ($\bar{p}$) were calculated from the full set of ancient Sardinians. Estimation of the allele frequency from the sample itself as well as population structure can have the effect that this pairwise correlation deviates from 0 even for unrelated pairs of individuals. To increase interpretability, we here centered this statistic by subtracting the mean of all pairwise values.

Using this basic approach enabled us to identify two pairs of first-degree relatives (expected $f(i,j) = 0.25$). The remainder of ancient Sardinian samples likely do not contain any first or second degree relatives (Supp. Fig. 12).

The first pair consists of a female and male sample, `SUC002` and `SUC003`, both sampled from the Su Crucifissu Mannu site. The broadly uniform value of estimated $f(i,j)$ around 0.25 throughout the genome (Supp. Fig. 12) suggests that these two samples are a parent-offspring pair, as full siblings would vary between $f = 0, 0.25$ and 0.5, depending on whether 0, 1 or 2 allele were co-inherited. Both samples had identical mtDNA haplogroup J1c3, providing some evidence that these pair of samples represent a mother and son.

The second pair consists of a female and male sample, `COR001` and `COR002`, both sampled from the Corona Moltana site. The estimated value of $f(i,j)$ centered around 0.25 with a broad range ranging from $0 - 0.5$ throughout the genome (Supp. Fig. 12), suggesting that these two samples are a full-sibling pair. Both samples carry an identical mtDNA haplogroup, K1b1a1.

In addition, we detected three genetic duplicates of three of our individuals (ISB001, LON003, MA82) by identifying pairs which had $f(i,j)$ close to 0.5 (results not shown). Identical uni-parentally inherited haplotypes and sampling location, close proximity on

the two-dimensional PCA, as well as overlapping radiocarbon ages corroborated this result. We therefore combined the three identical pairs into three single samples, and merged their reads for all subsequent analysis and reported data.

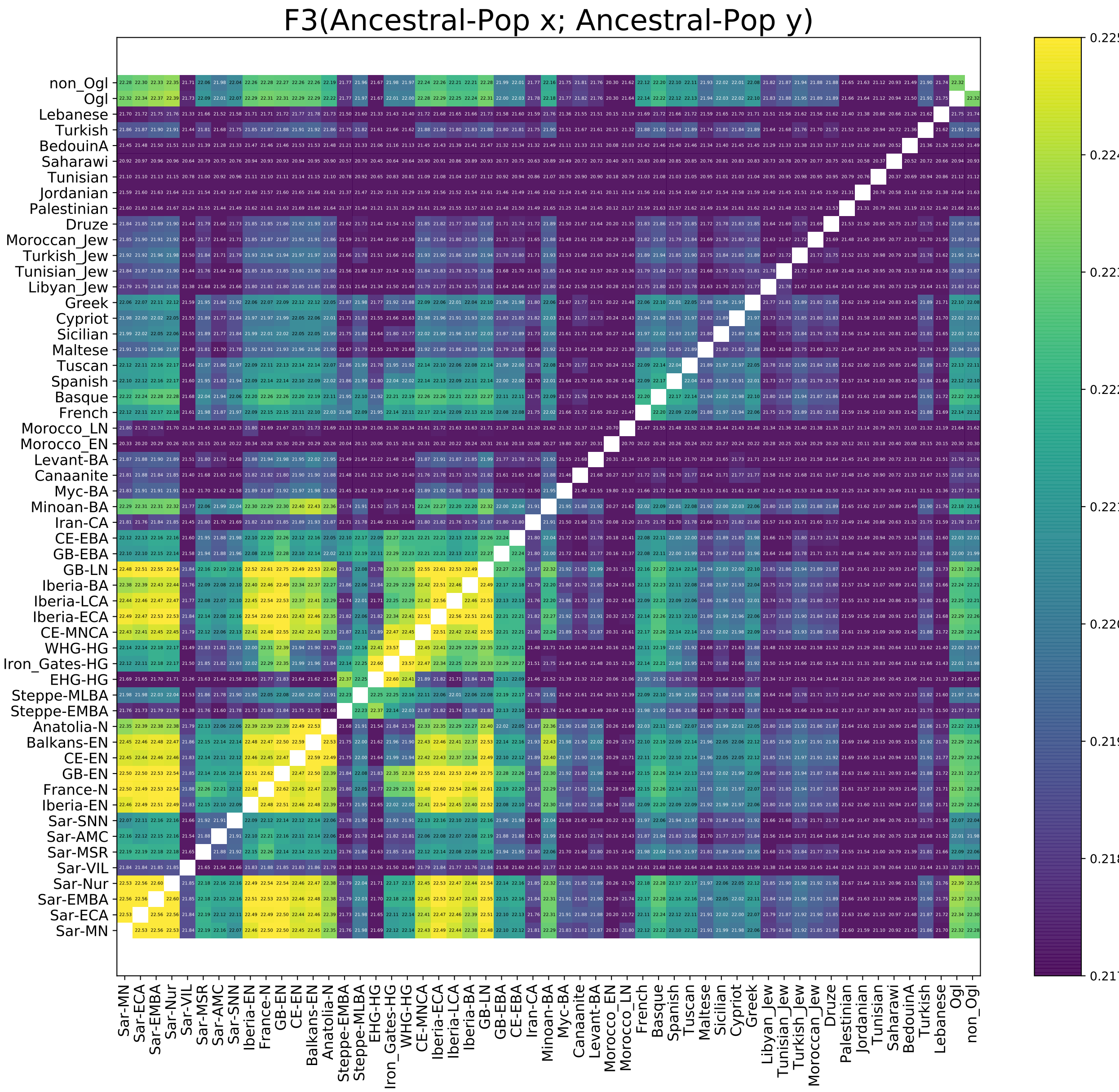

Supplementary Figure 10: Matrix of f3-outgroup "shared genetic drift" metrics of pairwise similarity. Populations are ordered broadly by period and geography (See Sup. Data 1G for legend to abbreviations). For post-Nuragic Sardinians, we group individuals by sample site. The two individuals from Corona-Moltana are not shown, as only two first-degree relatives are available.

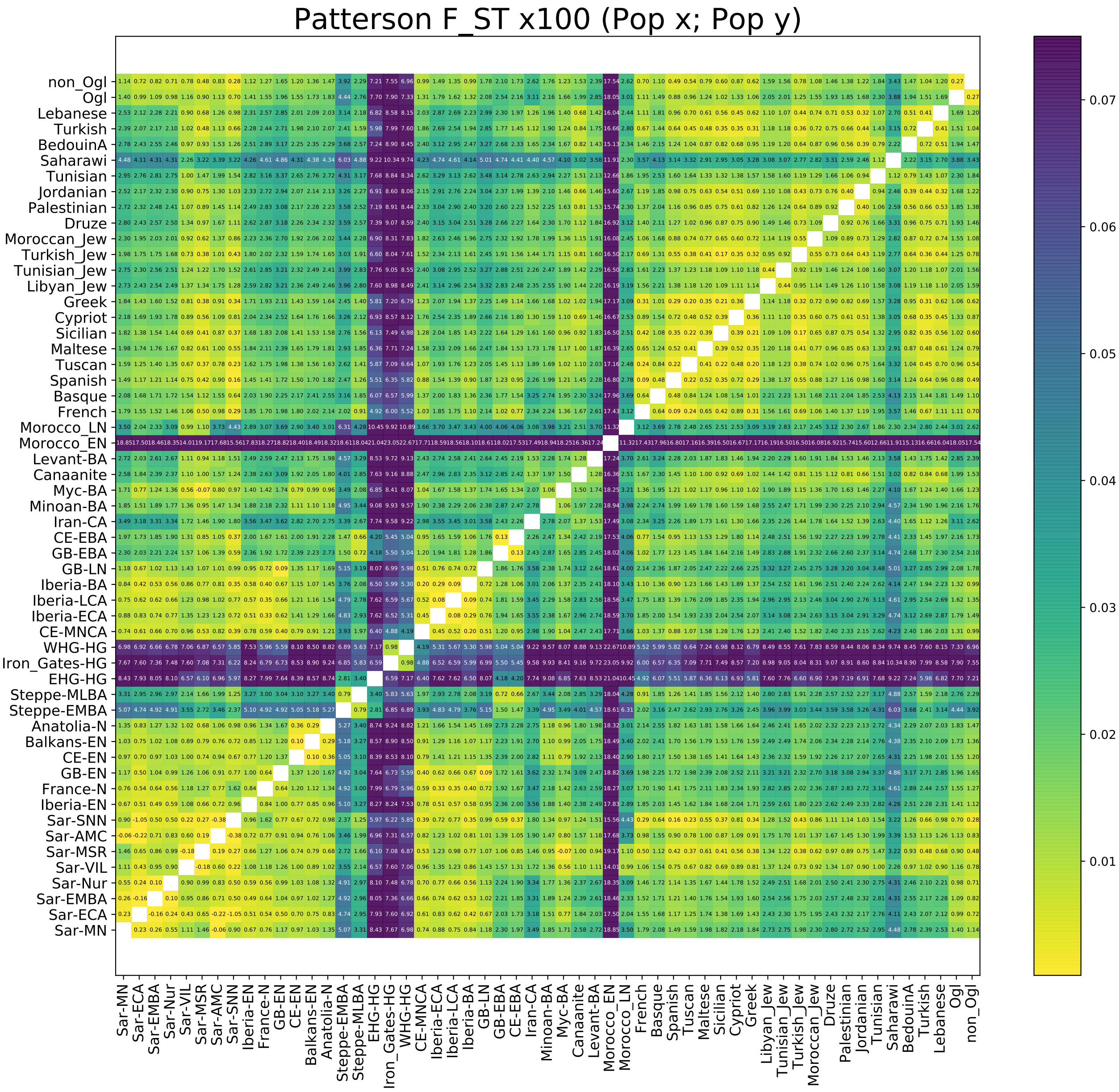

Supplementary Figure 11: Matrix of F_{ST} metrics of pairwise differentiation. Populations are ordered broadly by period and geography (See Sup. Data 1G for legend to abbreviations). For post-Nuragic Sardinians, we group individuals by sample site. These sites have typically low number of individuals and low coverage. This increases estimation variance and explains the sometimes negative values (which are possible for an unbiased F_{ST} estimators such as the one used here). The two individuals from Corona-Moltana are not shown, as only two first-degree relatives are available.

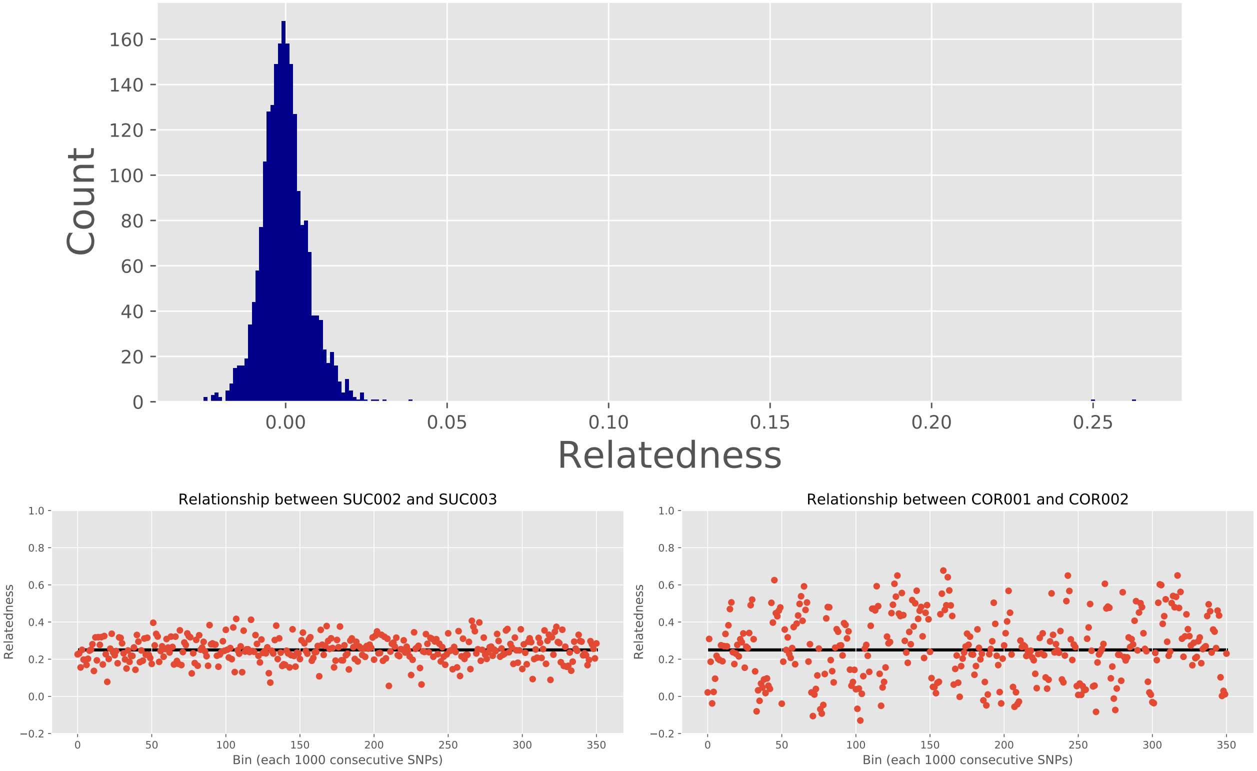

Supplementary Figure 12: Pairwise Relatedness estimates in ancient Sardinians. Upper panel: Histogram of all pairwise relatedness estimates for ancient Sardinians, plotted for all pairs with more than $10,000$ intersecting called SNPs. Only two pairs of samples have significantly elevated relatedness (SUC002/SUC003 and COR001/COR002, counts seen at just larger than 0.25). Lower panel: Estimated $f(i,j)$ for these two putatively related sample pairs, calculated genome-wide using bins of 1000 consecutive SNPs ordered along the reference genome.

Supplementary Note 5: Admixture Analysis with qpAdm.

Harald Ringbauer, Chi-Chun Liu For this analysis we use the software package Admixtools (https://github.com/DReichLab/AdmixTools, Version 5.1) which contains the qpAdm program. This tool utilizes the fact that f-statistics of an admixed population are a linear mixture of f-statistics using putative source population (together called "left" populations)[59,65].

The rank of a matrix of f-statistics with all pairs of a set of outgroup populations ("right" populations) does not increase when adding an admixed population to a left set containing all source populations, providing a means to assess whether the null model of admixture can be rejected. It is important to stress that a p-value from this approach is influenced by sample number and number of SNP markers, and when interpreting results, one must be cautious of the equivalent of Type II errors in the face of limited data.

The results, in particular the p-value that describes the model fit, also depend on the set of populations used as an outgroup. To assess the robustness of our results to the outgroup choice, we utilized two groups of populations as out-groups: First a set of 13 out-group populations from the Human Origins dataset that capture modern human variation ("**M13**": Ami, Biaka, Bougainville, Chukchi, Eskimo, Han, Ju-hoan-North, Karitiana, Mbuti, Papuan, She, Ulchi, Yoruba)[59]. Second, a group of 15 ancient samples ("**A15**": Mota, UstIshim, Kostenki14, GoyetQ116-1, Vestonice16, MA1, ElMiron, Villabruna, WHG, EHG, CHG, Iran-N, Natufian, Levant-N, Anatolia-N) whose value for disentangling divergent strains of ancestry present in Europe has been previously described[66]. If populations from the group "A15" (see table just below) were included in the left populations (WHG and/or Anatolia-N), we removed them from the right set, and used the remaining 14 and/or 13 populations as outgroup (labelled "A14"/"A13").

Both sets of outgroups yield qualitatively similar results for our analysis. For conciseness and clarity, in the following we present the results based on the A15 outgroup, as it supposedly reflects finer aspects of ancient west Eurasian genetic variation.

Sardinia in relation to mainland Hunter-Gatherer, Early European Farmer, and Steppe ancestries

We ran a two-way admixture model between Neolithic Anatolians (Anatolia-N) and Hunter Gatherers (WHG) and a three-way model with a Bronze Age Steppe group (Steppe-EMBA) as a proxy for Steppe ancestry. We tested various Neolithic European mainland populations, as well as ancient individuals of Sardinia grouped into three pre-Nuragic time periods and post-Nuragic archaeological sites (Supp. Tab. 1).

To extend beyond the three-way distal models, we also ran a five-way admixture model between Neolithic Anatolians (Anatolia-N), Hunter Gatherers (WHG), a Bronze Age Steppe group (Steppe-EMBA), adding Neolithic Iranians (Iran-N) and Neolithic North Africans (Morocco-EN) (Fig. 14).

This analysis revealed an almost complete absence of Iran-N and Steppe ancestry in ancient Sardinians from the Neolithic through the Nuragic period, while post-Nuragic Sardinian ancients as well as present-day Sardinians were modelled to partially have such

	Source Populations					Admixture Fractions			Standard Error		
Target	A	B	C	Outgroup	p-Value	A	B	C	A	B	C
Sar-MN	WHG	Anatolia-N	-	A13	0.376	0.177	0.823	-	0.014	0.014	-
Sar-ECA	WHG	Anatolia-N	-	A13	0.268	0.161	0.839	-	0.020	0.020	-
Sar-EMBA	WHG	Anatolia-N	-	A13	0.049	0.161	0.839	-	0.007	0.007	-
Sar-Nur	WHG	Anatolia-N	-	A13	0.134	0.163	0.837	-	0.009	0.009	-
Sar-VIL	WHG	Anatolia-N	-	A13	$1.7 \cdot 10^{-15}$	0.061	0.939	-	0.016	0.016	-
Sar-MSR	WHG	Anatolia-N	-	A13	$8.3 \cdot 10^{-3}$	0.093	0.907	-	0.018	0.018	-
Sar-AMC	WHG	Anatolia-N	-	A13	0.049	0.092	0.908	-	0.020	0.020	-
Sar-SNN	WHG	Anatolia-N	-	A13	$2.0 \cdot 10^{-6}$	0.159	0.841	-	0.018	0.018	-
Sar-COR	WHG	Anatolia-N	-	A13	$2.6 \cdot 10^{-5}$	0.169	0.831	-	0.018	0.018	-
France-N	WHG	Anatolia-N	-	A13	0.144	0.213	0.787	-	0.015	0.015	-
Iberia-EN	WHG	Anatolia-N	-	A13	0.375	0.087	0.913	-	0.011	0.011	-
Iberia-ECA	WHG	Anatolia-N	-	A13	$8.1 \cdot 10^{-6}$	0.274	0.726	-	0.010	0.010	-
Iberia-LCA	WHG	Anatolia-N	-	A13	0.075	0.251	0.749	-	0.009	0.009	-
CE-EN	WHG	Anatolia-N	-	A13	0.801	0.046	0.954	-	0.006	0.006	-
CE-MNCA	WHG	Anatolia-N	-	A13	0.324	0.312	0.688	-	0.010	0.010	-
CE-EBA	WHG	Anatolia-N	-	A13	0	0.299	0.701	-	0.012	0.012	-
CE-LBA	WHG	Anatolia-N	-	A13	0	0.298	0.702	-	0.011	0.011	-
Balkans-EN	WHG	Anatolia-N	-	A13	0.641	0.017	0.983	-	0.008	0.008	-
Balkans-MNCA	WHG	Anatolia-N	-	A13	0.614	0.068	0.932	-	0.005	0.005	-
Balkans-BA	WHG	Anatolia-N	-	A13	0	0.174	0.826	-	0.006	0.006	-
Sar-MN	WHG	Anatolia-N	Steppe-EMBA	A13	0.265	0.177	0.823	-0.000	0.016	0.023	0.026
Sar-ECA	WHG	Anatolia-N	Steppe-EMBA	A13	0.18	0.164	0.836	-0.000	0.023	0.032	0.036
Sar-EMBA	WHG	Anatolia-N	Steppe-EMBA	A13	0.032	0.162	0.838	-0.000	0.009	0.012	0.013
Sar-Nur	WHG	Anatolia-N	Steppe-EMBA	A13	0.089	0.163	0.837	-0.000	0.010	0.014	0.016
Sar-VIL	WHG	Anatolia-N	Steppe-EMBA	A13	$5.3 \cdot 10^{-13}$	0.000	0.866	0.134	0.018	0.026	0.029
Sar-MSR	WHG	Anatolia-N	Steppe-EMBA	A13	0.524	0.053	0.798	0.149	0.021	0.032	0.037
Sar-AMC	WHG	Anatolia-N	Steppe-EMBA	A13	0.202	0.060	0.839	0.101	0.024	0.033	0.040
Sar-SNN	WHG	Anatolia-N	Steppe-EMBA	A13	0.523	0.102	0.694	0.204	0.020	0.029	0.032
Sar-COR	WHG	Anatolia-N	Steppe-EMBA	A13	$1.9 \cdot 10^{-4}$	0.141	0.761	0.098	0.021	0.032	0.037
France-N	WHG	Anatolia-N	Steppe-EMBA	A13	0.093	0.213	0.787	-0.000	0.018	0.023	0.027
Iberia-EN	WHG	Anatolia-N	Steppe-EMBA	A13	0.243	0.087	0.913	-0.000	0.012	0.017	0.019
Iberia-ECA	WHG	Anatolia-N	Steppe-EMBA	A13	$4.7 \cdot 10^{-6}$	0.273	0.727	-0.000	0.011	0.016	0.018
Iberia-LCA	WHG	Anatolia-N	Steppe-EMBA	A13	0.045	0.251	0.749	-0.000	0.012	0.015	0.018
CE-EN	WHG	Anatolia-N	Steppe-EMBA	A13	0.656	0.046	0.954	-0.000	0.007	0.010	0.012
CE-MNCA	WHG	Anatolia-N	Steppe-EMBA	A13	0.28	0.308	0.679	0.013	0.011	0.015	0.018
CE-EBA	WHG	Anatolia-N	Steppe-EMBA	A13	$3.0 \cdot 10^{-4}$	0.126	0.414	0.460	0.006	0.008	0.010
CE-LBA	WHG	Anatolia-N	Steppe-EMBA	A13	0.105	0.128	0.403	0.468	0.008	0.011	0.013
Balkans-EN	WHG	Anatolia-N	Steppe-EMBA	A13	0.511	0.016	0.984	-0.000	0.009	0.013	0.015
Balkans-MNCA	WHG	Anatolia-N	Steppe-EMBA	A13	0.485	0.068	0.932	-0.000	0.006	0.008	0.010
Balkans-BA	WHG	Anatolia-N	Steppe-EMBA	A13	20.117	0.117	0.703	0.180	0.006	0.009	0.011

Supplementary Table 1: Admixture proportions of ancient European populations inferred by qpAdm. The upper block of models are two-way models between western hunter gatherer (WHG) and Anatolia Neolithic samples (Anatolia-N) as sources. The lower block of models are three-way models, adding Steppe-EMBA samples as third source. Steppe-EMBA is abbreviated as Steppe in the table. See Sup. Data 1G for legend to abbreviations.

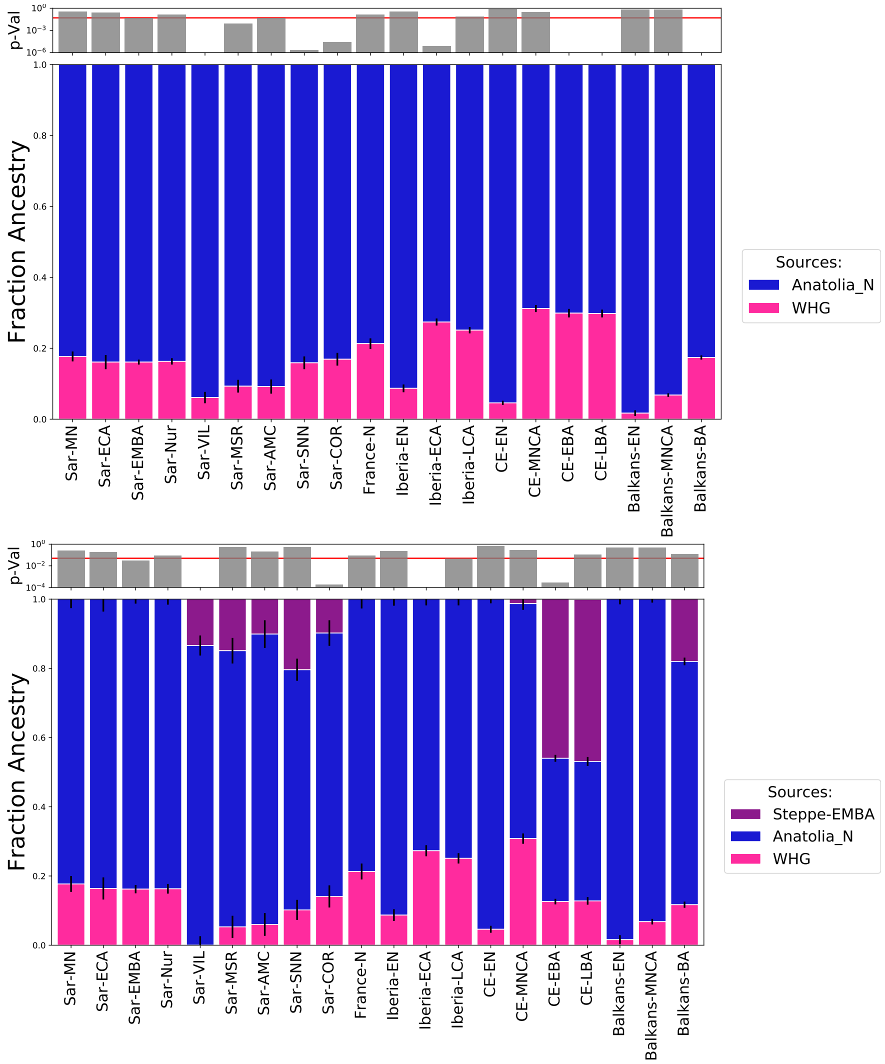

Supplementary Figure 13: Visualization of two-way (top) and three-way admixture (bottom) of ancient European populations. Error bars depict standard errors. Upper panel visualizes p-values whether this model of admixture can be rejected. The red line depicts a standard significance cut off ($p = 0.05$).

ancestries. Corroborating results from the 3-way model, Sardinians up until the Nuragic carry a WHG component of about $15-20\%$, which remains constant from the Middle Neolithic to the Nuragic period.

Significant proportions of the ancient North African ancestry component appear in the ancient Villamar samples (Punic, 1st millennium BCE). In later ancients as well as in present-day Sardinians, this ancestry remains at much lower levels than in Villamar.

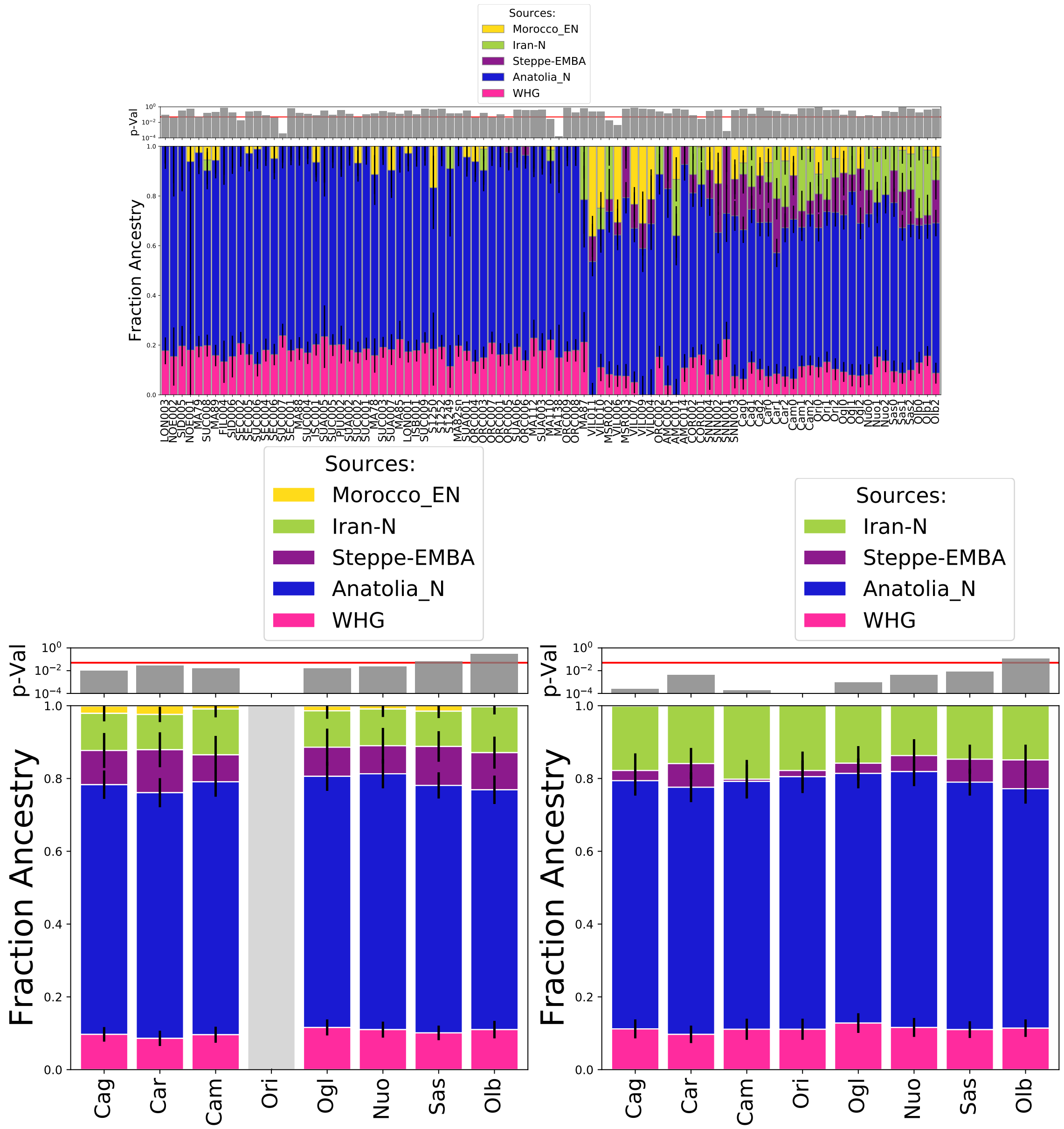

Supplementary Figure 14: Visualization of five-way distal qpAdm models. Error bars depict standard errors. Upper Panel: All ancient Sardinian Individuals (sorted by age, oldest left) and 3 modern individuals from each Sardinian province(right) as target. Lower Panel Left: Present-day Sardinians grouped into provinces as target. Lower Panel Right: Same as left, but with Morocco-EN moved to the outgroup. Upper sub-panels visualize p-values whether this model of admixture can be rejected. The red lines depicts a standard significance cut off ($p = 0.05$) and gray bars depict models with infeasible admixture coefficients outside [0,1]. The bottom right panel shows a decrease in fit using a 4-way distal model with Morocco-EN as the outgroup (Supp. Fig. 11), as expected if present-day Sardinians harboured some fraction of ancestry related to Morocco-EN.

Continuity on Sardinia through the Nuragic period

We tested models of continuity within the ancient Sardinians with qpAdm, using A15 and A15 plus Morocco-EN as outgroups (Supp. Tab. 2). We used the four pre-Nuragic groups described in the main text: Middle Neolithic ('Sar-MN', 4,100-3,500 BCE, $n = 6$), Early Copper Age ('Sar-ECA', 3,500-2,500 BCE, $n = 3$), Early Middle Bronze Age ('Sar-EMBA', 2,500-1,500 BCE, $n = 27$) and Nuragic ('Sar-Nur', 1,500-900 BCE, $n = 16$). Similarly, we tested a model of continuity from these ancient Sardinian groups into modern samples of the provinces Ogliastra ($n = 419$) and Cagliari ($n = 289$).

Target	Source	p-value[1]	p-value[2]	SNPs[1]	SNPs[2]
Sar-ECA	Sar-MN	0.175	0.041	548270	514195
Sar-EMBA	Sar-ECA	0.769	0.217	392596	337400
Sar-Nur	Sar-EMBA	0.765	0.282	449947	390664
Sar-Nur	Sar-MN	0.153	0.795	454877	402050
Cagliari	Sar-MN	$3.2 \cdot 10^{-9}$	$4.0 \cdot 10^{-3}$	112513	62767
Cagliari	Sar-ECA	$2.3 \cdot 10^{-5}$	$1.8 \cdot 10^{-4}$	108685	60087
Cagliari	Sar-EMBA	$8.2 \cdot 10^{-65}$	$2.9 \cdot 10^{-40}$	116028	65796
Cagliari	Sar-Nur	$4.4 \cdot 10^{-45}$	$9.7 \cdot 10^{-24}$	116005	65760
Ogliastra	Sar-MN	$1.3 \cdot 10^{-6}$	0.051	119637	70696
Ogliastra	Sar-ECA	$4.2 \cdot 10^{-4}$	$1.5 \cdot 10^{-3}$	115223	67413
Ogliastra	Sar-EMBA	$2.2 \cdot 10^{-44}$	$2.2 \cdot 10^{-28}$	123952	74468
Ogliastra	Sar-Nur	$6.2 \cdot 10^{-28}$	$4.9 \cdot 10^{-14}$	124032	74578
Sar-MN	France-N	0.106	0.378	548276	510309
Sar-MN	Iberia-EN	$1.5 \cdot 10^{-8}$	$3.2 \cdot 10^{-6}$	539337	496174
Sar-MN	Iberia-ECA	$3.1 \cdot 10^{-17}$	$4.3 \cdot 10^{-11}$	493078	443422
Sar-MN	CE-EN	$1.4 \cdot 10^{-35}$	$2.1 \cdot 10^{-24}$	398397	340991
Sar-MN	CE-MNCA	$1.2 \cdot 10^{-39}$	$2.2 \cdot 10^{-21}$	496745	448014
Sar-MN	Balkans-EN	$1.4 \cdot 10^{-41}$	$3.6 \cdot 10^{-29}$	467465	415058
Sar-MN	Balkans-MNCA	$3.6 \cdot 10^{-22}$	$6.1 \cdot 10^{-14}$	339852	276051
Sar-MN	GB-EN	$4.6 \cdot 10^{-13}$	$6.4 \cdot 10^{-8}$	477526	426711
Sar-MN	GB-LN	$3.0 \cdot 10^{-9}$	$8.6 \cdot 10^{-4}$	397777	338863
Sar-MN	Morocco-LN	$6.0 \cdot 10^{-14}$	$1.2 \cdot 10^{-17}$	173096	150517

Supplementary Table 2: Support for models of continuity between two populations inferred by qpAdm. Upper block: Models for continuity between ancient Sardinian groups. Middle block: Models for continuity between ancient and present-day Sardinian groups. Lower block: Models of continuity between the oldest ancient Sardinian group and Neolithic mainland populations. We tested two outgroups: The standard set A15 ([1]), and also A15 with Morocco-EN included as additional outgroup ([2]) to test for differential ancient North African ancestry. We also show the number of intersecting SNPs used in the qpAdm analysis.

Post-Nuragic admixture of present-day Sardinians

Next, we fitted present-day Sardinian provinces as an admixture between Nuragic-Age Sardinians and potential regional source populations.

Two-way admixture models

Initially, we tested a model of the Cagliari samples as a mix of Nuragic period Sardinian samples and various mainland source populations to narrow down a plausible model for Sardinian admixture. We chose Cagliari, as PCA analysis revealed that it is a relatively homogeneous population in the center of present-day Sardinian variation (Fig. 2, main text) and because we had a high number of samples from this province ($n = 289$). To maximize the power to reject admixture models, we used the full A15 dataset together as an outgroup.

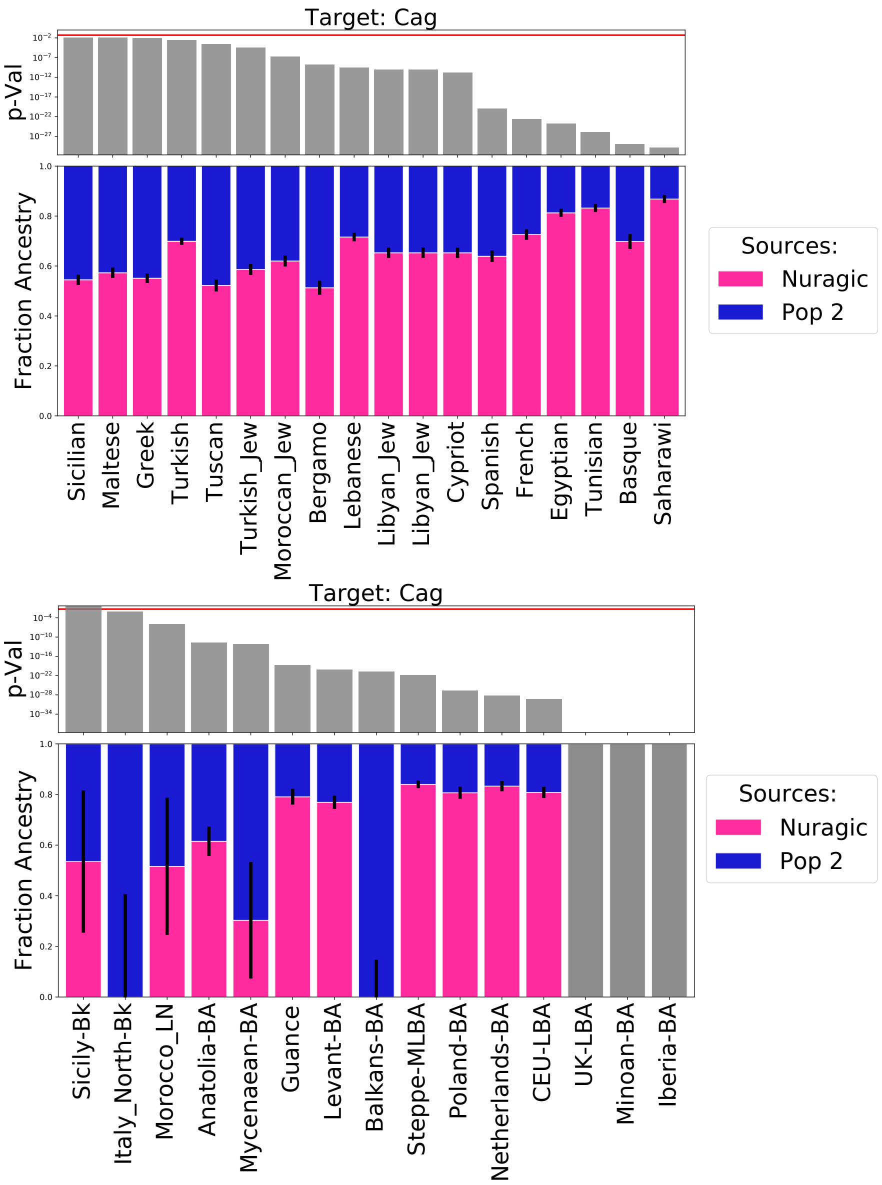

Supplementary Figure 15: Visualization of various two-way admixture models of $n = 289$ modern Cagliari samples. We hold one source fixed to Ancient Sardinian Nuragic samples, and tested several putative source populations (x-axis). Error bars depict standard errors. Gray bars depict infeasible models with inferred admixture fractions outside [0,1]. Top: Present-day source populations. Bottom: Ancient source populations.

	Source Populations					Admixture Fractions			Standard Error		
**Target**	**A**	**B**	**C**	**Outgroup**	**p-value**	**A**	**B**	**C**	**A**	**B**	**C**
Cag	Sar-Nur	Sicilian	-	A15	0.011	0.545	0.455	-	0.021	0.021	-
Cag	Sar-Nur	Maltese	-	A15	0.011	0.573	0.427	-	0.021	0.021	-
Cag	Sar-Nur	Greek	-	A15	$7.8 \cdot 10^{-3}$	0.551	0.449	-	0.019	0.019	-
Cag	Sar-Nur	Turkish	-	A15	$2.4 \cdot 10^{-3}$	0.699	0.301	-	0.014	0.014	-
Cag	Sar-Nur	Tuscan	-	A15	$2.3 \cdot 10^{-4}$	0.522	0.478	-	0.024	0.024	-
Cag	Sar-Nur	Turkish-Jew	-	A15	$3.5 \cdot 10^{-5}$	0.586	0.414	-	0.022	0.022	-
Cag	Sar-Nur	Moroccan-Jew	-	A15	$1.6 \cdot 10^{-7}$	0.620	0.380	-	0.022	0.022	-
Cag	Sar-Nur	Bergamo	-	A15	$1.7 \cdot 10^{-9}$	0.513	0.487	-	0.028	0.028	-
Cag	Sar-Nur	Lebanese	-	A15	$2.6 \cdot 10^{-10}$	0.716	0.284	-	0.017	0.017	-
Cag	Sar-Nur	Libyan-Jew	-	A15	$9.0 \cdot 10^{-11}$	0.653	0.347	-	0.021	0.021	-
Cag	Sar-Nur	Libyan-Jew	-	A15	$9.0 \cdot 10^{-11}$	0.653	0.347	-	0.021	0.021	-
Cag	Sar-Nur	Cypriot	-	A15	$1.5 \cdot 10^{-11}$	0.653	0.347	-	0.021	0.021	-
Cag	Sar-Nur	Spanish	-	A15	$1.1 \cdot 10^{-20}$	0.639	0.361	-	0.023	0.023	-
Cag	Sar-Nur	French	-	A15	$2.6 \cdot 10^{-23}$	0.726	0.274	-	0.021	0.021	-
Cag	Sar-Nur	Egyptian	-	A15	$1.5 \cdot 10^{-24}$	0.813	0.187	-	0.016	0.016	-
Cag	Sar-Nur	Tunisian	-	A15	$1.3 \cdot 10^{-26}$	0.832	0.168	-	0.016	0.016	-
Cag	Sar-Nur	Basque	-	A15	$1.0 \cdot 10^{-29}$	0.698	0.302	-	0.030	0.030	-
Cag	Sar-Nur	Saharawi	-	A15	$1.4 \cdot 10^{-30}$	0.868	0.132	-	0.016	0.016	-
Cag	Sar-Nur	Sicily-Bk	-	A15	0.353	0.417	0.583	-	0.427	0.427	-
Cag	Sar-Nur	Morocco-LN	-	A15	$1.1 \cdot 10^{-5}$	0.410	0.590	-	0.297	0.297	-
Cag	Sar-Nur	Morocco-EN	-	A15	$3.1 \cdot 10^{-10}$	0.913	0.087	-	0.014	0.014	-
Cag	Sar-Nur	Myc-BA	-	A15	$3.0 \cdot 10^{-13}$	0.165	0.835	-	0.249	0.249	-
Cag	Sar-Nur	Anatolia-BA	-	A15	$1.7 \cdot 10^{-15}$	0.552	0.448	-	0.069	0.069	-
Cag	Sar-Nur	Steppe-MLBA	-	A15	$1.5 \cdot 10^{-18}$	0.818	0.182	-	0.014	0.014	-
Cag	Sar-Nur	Canaanite	-	A15	$8.2 \cdot 10^{-21}$	0.721	0.279	-	0.026	0.026	-
Cag	Sar-Nur	Guanche	-	A15	$2.9 \cdot 10^{-24}$	0.771	0.229	-	0.032	0.032	-
Cag	Sar-Nur	Netherlands-BA	-	A15	$2.5 \cdot 10^{-24}$	0.802	0.198	-	0.018	0.018	-
Cag	Sar-Nur	CE-LBA	-	A15	$4.9 \cdot 10^{-26}$	0.775	0.225	-	0.020	0.020	-
Cag	Sar-Nur	CE-EBA	-	A15	$2.6 \cdot 10^{-26}$	0.779	0.221	-	0.019	0.019	-
Cag	Sar-Nur	Levant-BA	-	A15	$9.9 \cdot 10^{-27}$	0.766	0.234	-	0.032	0.032	-
Cag	Sar-Nur	GB-LBA	-	A15	$6.9 \cdot 10^{-27}$	0.796	0.204	-	0.019	0.019	-
Cag	Sar-Nur	Italy-North-Bk	-	A15	$-1.0 \cdot 10^{0}$	0.000	0.000	-	0.000	0.000	-
Cag	Sar-Nur	Balkans-BA	-	A15	$-1.0 \cdot 10^{0}$	0.000	0.000	-	0.000	0.000	-
Cag	Sar-Nur	Minoan-BA	-	A15	$-1.0 \cdot 10^{0}$	0.000	0.000	-	0.000	0.000	-
Cag	Sar-Nur	Iberia-BA	-	A15	$-1.0 \cdot 10^{0}$	0.000	0.000	-	0.000	0.000	-

Supplementary Table 3: Admixture proportions inferred by qpAdm for target Cagliari, with one source set to ancient Nuragic ($n = 15$). Infeasible models (with admixture fractions outside [0,1]) are set to $p = -1.0$. Top: Present-day source populations. Bottom: Ancient source populations

Three-way admixture models

The two-way admixture model suggested Sicily as the best fitting source for admixture into Sardinia after the Nuragic period ($p = 0.031$ for modern Sicilians, and $p = 0.43$ for Beaker period Sicilian samples "Sicily-Bk", Supp. Tab. 3).

Given the history of the region, one possibility is Sicily serves in the two-way model as a proxy for more complex set of ancestries that entered Sardinia after the Nuragic period. To investigate this possibility, we tested potential three-way models that include two present-day populations in addition to the Nuragic Sardinian population as sources (Supp. Tab. 4). We identified several three-way models that fit as well or even better than Sicily as a single additional source. The best fitting models (of a subset of possible trios we tested) are generally a combination of one population from a set of "northern Mediterranean" population (including Lombardy [Bergamo], Greek, Tuscan, French) and another population from a set of broadly "eastern Meditarranean" populations (including Lebanese, Turkish and north African Jewish populations). Sicilian and Maltese samples also produce viable three-way admixture models but the estimates of ancestry for the N. Mediterranean component shrink to small values, bringing the fitted parameters in effect towards the two-way mixture models. We interpret this as reflecting that Maltese and Sicilian are a mixture of N. Mediterranean and E. Mediterranean ancestries such that they can serve as single-source proxies in two-way admixture models for a more complex admixture process.

Using the three-way admixture models, we investigated substructure within Sardinia by fitting all 9 provinces of Sardinia as a three-way admixture between "northern Mediterranean" and "eastern Meditarranean" sources (Supp. Tab. 5 and Supp. Fig. 17). Again, we used A15 as set of right outgroup populations. The results demonstrate geographic substructure within Sardinia with respect to the admixture components. The highest "northern Mediterranean" component is found in the north eastern part of the island (Olbia and to a lesser degree Sassari), while the "eastern Mediterranean" component is highest in the southwestern provinces (Carbonia and Medio Campidano). The more isolated provinces, Nuoro and Ogliastra, are inferred to have among the lowest proportions of post-Nuragic admixture.

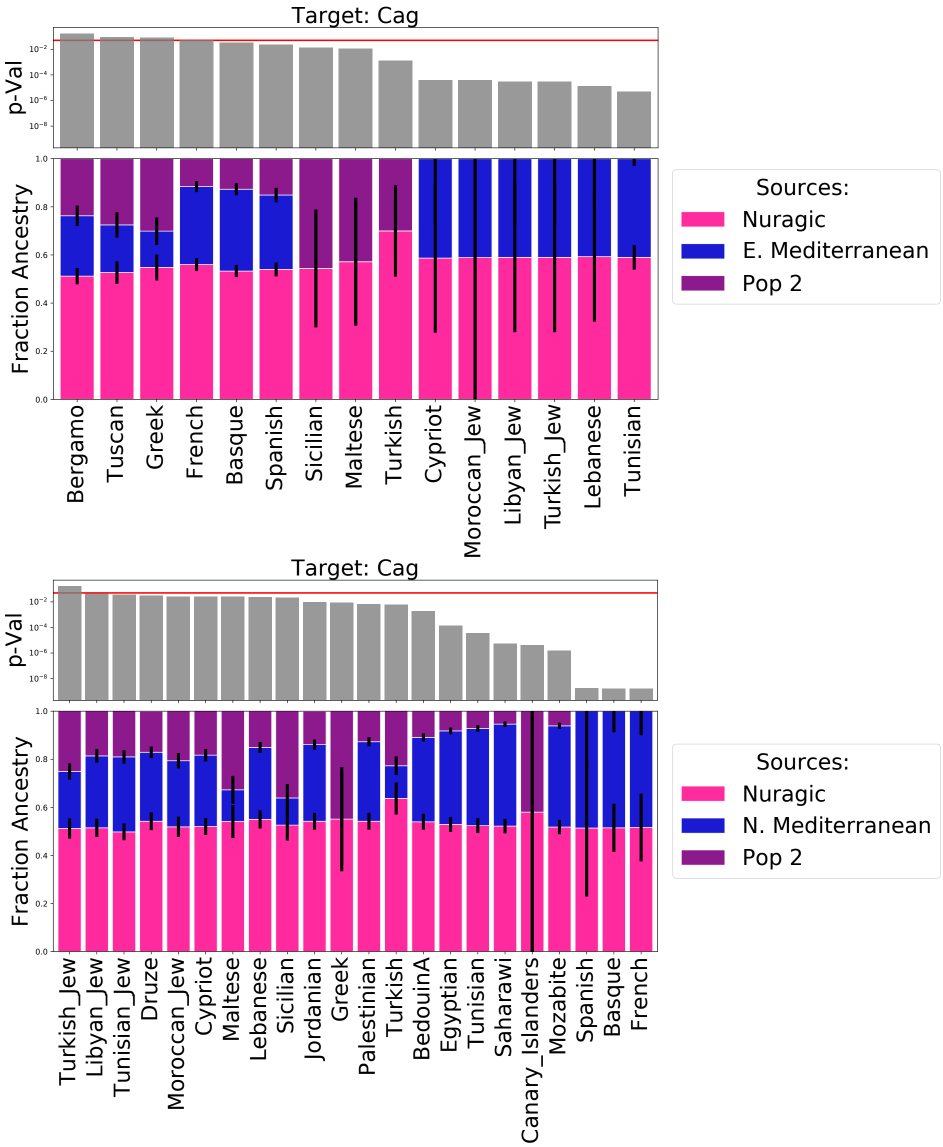

Supplementary Figure 16: Visualization of various three-way admixture models of modern Cagliari individuals. Top: Eastern Mediterranean (proxy: Turkish Jew) as fixed source additional to Sardinian Nuragic. Bottom: Northern Mediterranean (proxy: Lombardy ['Bergamo' in HOA data]) as fixed source additional to Sardinian Nuragic. Error bars depict standard errors. If estimated admixture proportions are outside $[0, 1]$, we depict the two-way model with the highest p-value.

	Source Populations					Admixture Fractions			Standard Error		
Target	A	B	C	Outgroup	p-Value	A	B	C	A	B	C
Cag	Sar-Nur	Lombardy	Turkish-Jew	A15 0.168	0.512	0.237	0.251	0.021	0.042	0.034	
Cag	Sar-Nur	Lombardy	Libyan-Jew	A15	0.046	0.515	0.299	0.186	0.023	0.038	0.028
Cag	Sar-Nur	Lombardy	Tunisian-Jew	A15	0.037	0.498	0.312	0.191	0.022	0.035	0.028
Cag	Sar-Nur	Lombardy	Druze	A15	0.031	0.542	0.287	0.170	0.022	0.037	0.024
Cag	Sar-Nur	Lombardy	Moroccan-Jew	A15	0.026	0.519	0.275	0.206	0.022	0.042	0.032
Cag	Sar-Nur	Lombardy	Cypriot	A15	0.026	0.520	0.297	0.184	0.021	0.035	0.026
Cag	Sar-Nur	Lombardy	Maltese	A15	0.025	0.541	0.132	0.327	0.025	0.069	0.058
Cag	Sar-Nur	Lombardy	Lebanese	A15	0.023	0.550	0.299	0.151	0.024	0.038	0.023
Cag	Sar-Nur	Lombardy	Sicilian	A15	0.021	0.526	0.113	0.361	0.022	0.064	0.058
Cag	Sar-Nur	Lombardy	Jordanian	A15	$10.0 \cdot 10^{-3}$	0.542	0.319	0.138	0.023	0.035	0.021
Cag	Sar-Nur	Lombardy	Greek	A15	$8.7 \cdot 10^{-3}$	0.551	-0.000	0.449	0.037	0.217	0.191
Cag	Sar-Nur	Lombardy	Palestinian	A15	$7.0 \cdot 10^{-3}$	0.542	0.331	0.127	0.024	0.035	0.019
Cag	Sar-Nur	Lombardy	Turkish	A15	$6.3 \cdot 10^{-3}$	0.637	0.136	0.228	0.033	0.067	0.039
Cag	Sar-Nur	Lombardy	BedouinA	A15	$2.0 \cdot 10^{-3}$	0.540	0.351	0.109	0.024	0.033	0.017
Cag	Sar-Nur	Lombardy	Egyptian	A15	$1.4 \cdot 10^{-4}$	0.529	0.389	0.082	0.025	0.031	0.014
Cag	Sar-Nur	Lombardy	Tunisian	A15	$3.7 \cdot 10^{-5}$	0.524	0.404	0.072	0.025	0.031	0.014
Cag	Sar-Nur	Lombardy	Saharawi	A15	$5.4 \cdot 10^{-6}$	0.522	0.424	0.054	0.026	0.030	0.011
Cag	Sar-Nur	Lombardy	Canary-Islanders	A15	$4.3 \cdot 10^{-6}$	0.580	-0.000	0.420	0.408	2.270	1.867
Cag	Sar-Nur	Lombardy	Mozabite	A15	$1.6 \cdot 10^{-6}$	0.518	0.421	0.061	0.026	0.030	0.013
Cag	Sar-Nur	Lombardy	Spanish	A15	$1.8 \cdot 10^{-9}$	0.514	0.486	0.000	0.054	0.285	0.247
Cag	Sar-Nur	Lombardy	Basque	A15	$1.7 \cdot 10^{-9}$	0.515	0.485	0.000	0.034	0.100	0.088
Cag	Sar-Nur	Lombardy	French	A15	$1.6 \cdot 10^{-9}$	0.516	0.484	0.000	0.051	0.141	0.100
Cag	Sar-Nur	Turkish-Jew	Lombardy	A15 0.168	0.512	0.251	0.237	0.021	0.034	0.042	
Cag	Sar-Nur	Turkish-Jew	Tuscan	A15 0.09	0.527	0.198	0.275	0.020	0.047	0.053	
Cag	Sar-Nur	Turkish-Jew	Greek	A15 0.079	0.548	0.151	0.302	0.018	0.054	0.057	
Cag	Sar-Nur	Turkish-Jew	French	A15 0.05	0.560	0.324	0.116	0.019	0.027	0.023	
Cag	Sar-Nur	Turkish-Jew	Basque	A15	0.034	0.533	0.340	0.128	0.021	0.025	0.025
Cag	Sar-Nur	Turkish-Jew	Spanish	A15	0.023	0.540	0.309	0.151	0.020	0.029	0.030
Cag	Sar-Nur	Turkish-Jew	Sicilian	A15	0.013	0.544	-0.000	0.456	0.029	0.226	0.245
Cag	Sar-Nur	Turkish-Jew	Maltese	A15	0.012	0.572	-0.000	0.428	0.026	0.261	0.266
Cag	Sar-Nur	Turkish-Jew	Turkish	A15	$1.2 \cdot 10^{-3}$	0.700	-0.000	0.300	0.058	0.190	0.135
Cag	Sar-Nur	Turkish-Jew	Cypriot	A15	$3.8 \cdot 10^{-5}$	0.587	0.413	0.000	0.047	0.310	0.275
Cag	Sar-Nur	Turkish-Jew	Moroccan-Jew	A15	$3.7 \cdot 10^{-5}$	0.589	0.411	0.000	0.128	1.650	1.544
Cag	Sar-Nur	Turkish-Jew	Libyan-Jew	A15	$2.8 \cdot 10^{-5}$	0.590	0.410	0.000	0.048	0.311	0.278
Cag	Sar-Nur	Turkish-Jew	Libyan-Jew	A15	$2.8 \cdot 10^{-5}$	0.590	0.410	0.000	0.048	0.311	0.278
Cag	Sar-Nur	Turkish-Jew	Lebanese	A15	$1.4 \cdot 10^{-5}$	0.593	0.407	0.000	0.075	0.270	0.202
Cag	Sar-Nur	Turkish-Jew	Tunisian	A15	$4.9 \cdot 10^{-6}$	0.590	0.410	0.000	0.028	0.051	0.030

Supplementary Table 4: Admixture proportions inferred by qpAdm for three-way admixture models. We modeled present-day Sardinian Cagliari with one source fixed to ancient Nuragic Sardinian individuals. Top: northern Mediterranean (proxy: Lombardy ['Bergamo' in HOA data]) as fixed Source 2). Bottom: eastern Mediterranean (proxy: Turkish Jew) as fixed Source 2. Only models with feasible admixture fractions are shown (see also Fig. 16)

	Source Populations					Admixture Fractions			Standard Error		
Target	A	B	C	Outgroup	p-Value	A	B	C	A	B	C
Cag	Sar-Nur	Lombardy	Turkish-Jew	A15 0.168	0.512	0.237	0.251	0.021	0.042	0.034	
Car	Sar-Nur	Lombardy	Turkish-Jew	A15 0.639	0.455	0.248	0.297	0.023	0.047	0.038	
Cam	Sar-Nur	Lombardy	Turkish-Jew	A15 0.172	0.515	0.173	0.312	0.024	0.044	0.036	
Ori	Sar-Nur	Lombardy	Turkish-Jew	A15	$7.4 \cdot 10^{-3}$	0.557	0.173	0.270	0.025	0.050	0.041
Ogl	Sar-Nur	Lombardy	Turkish-Jew	A15 0.202	0.582	0.240	0.178	0.025	0.046	0.039	
Nuo	Sar-Nur	Lombardy	Turkish-Jew	A15 0.26	0.586	0.228	0.186	0.024	0.046	0.039	
Sas	Sar-Nur	Lombardy	Turkish-Jew	A15 0.564	0.477	0.309	0.214	0.021	0.039	0.033	
Olb	Sar-Nur	Lombardy	Turkish-Jew	A15 0.353	0.412	0.423	0.165	0.029	0.053	0.043	

Supplementary Table 5: Admixture proportions inferred by qpAdm for three-way admixture model of present-day Sardinian populations. We fit a three-way model of ancient Sardinian Nuragic ancestry, Eastern Mediterranean ancestry (proxy: Turkish-Jew) and Northern Mediterranean ancestry (proxy: Lombardy)

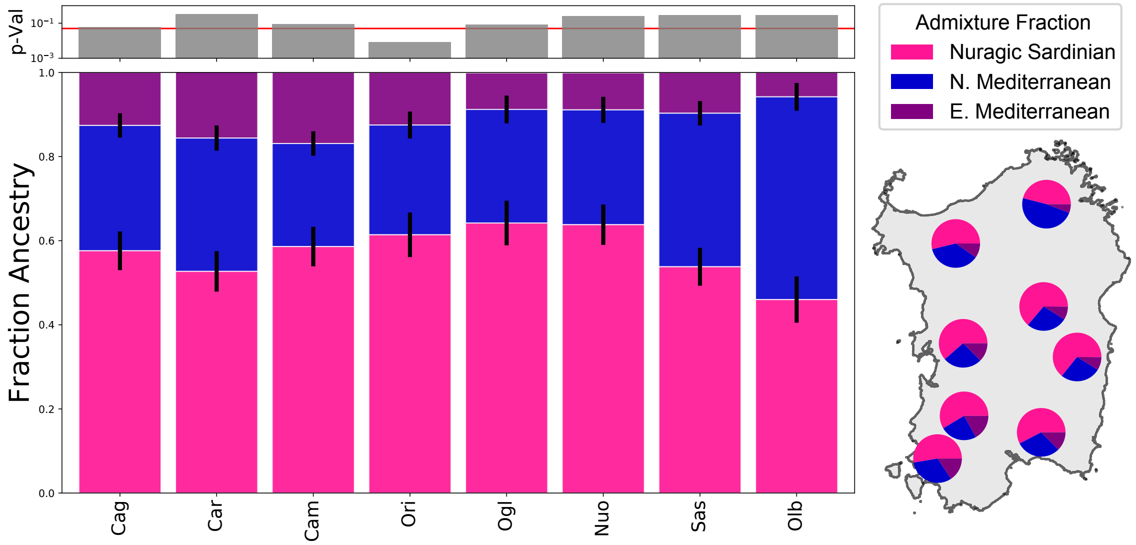

Supplementary Figure 17: Visualization of admixture proportions of a three-way admixture model of modern Sardinian populations. We fit a three-way model of ancient Sardinian Nuragic ancestry, Eastern Mediterranean ancestry (proxy: Lebanese) and Northern Mediterranean ancestry (proxy: Tuscan). Error bars depict standard errors.

Four-way admixture models for present-day Sardinians

To assess the robustness of the three-way model, we tested a four-way admixture model with a proxy for ancient N. African ancestry (pre-colonial Guanche, Canary Islands, $n = 5$)[67] as an additional source to our best fit three-way model with sources Nuragic Sardinia, eastern Mediterranean (proxy: Lebanese) and northern Mediterranean (proxy: Tuscan). This four-way model does not yield an improved model fit and the Guanche ancestry component is inferred to be very low (Supp. Fig. 18). We additionally tested $n = 5$ Neolithic Moroccans[64] as a fourth source (instead of Guanche), which yielded qualitatively very similar admixture fractions (results not shown). We note that the Eastern Mediterranean present day source (proxy: Lebanese) potentially has some North African Ancestry itself, which could absorb some of overall North African ancestry.

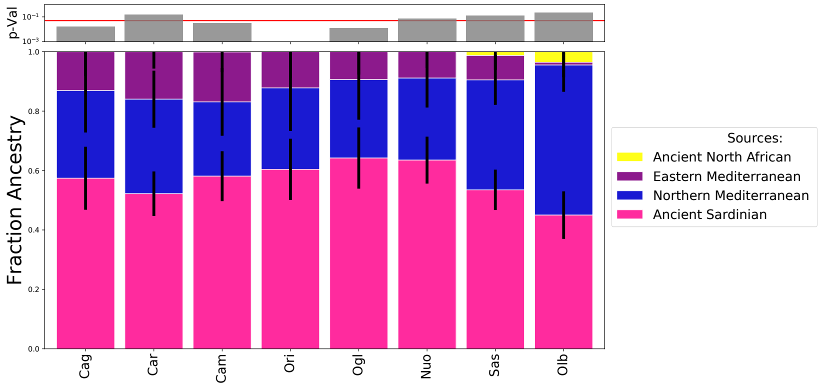

Supplementary Figure 18: Robustness of three-way admixture model to inclusion of a fourth ancient north African source. We fit a four-way model of mixture between ancient Sardinian Nuragic ancestry, Eastern Mediterranean ancestry (proxy: Lebanese), Northern Mediterranean ancestry (proxy: Tuscan), and Ancient North African ancestry (proxy: ancient Guanche, Canary islands). Error bars depict standard errors.

Refinement of admixture models using post-Nuragic ancient samples

To refine the models of 3-way admixture between the Nuragic and present, we incorporated a set of individuals from several post-Nuragic archaeological sites in a secondary phase of sampling and data analysis.

Models of continuity from the Nuragic to present

As above, we first assess one-way models of continuity. The results above suggest such models of continuity between the Nuragic period and present do not fit well, likely because of some form of post-Nuragic admixture. We extend that analysis by assessing one-way models of continuity using the finer resolution provided by the post-Nuragic sites. We find that no one-way model of continuity from the Nuragic period ('Sar-Nur') with any later period work well (Supp. Tab. 6). We also observe that models of one-way continuity from the Villamar samples ('Sar-VIL' in the table) do not work well with later samples. However, later samples work relatively well in models of one-way continuity among each other and also with present-day samples ('Sar-MSR' and 'Sar-COR' p-value of 7.9×10^{-4}, the remainder all $> 10^{-3}$).

Multi-way admixture models of post-Nuragic samples

We next ran `qpAdm` analyses with regional ancient DNA sources and present the results here (see section above for fitting with more distant sources).

Specifically we allowed each target as a mixture of Nuragic period Sardinian ancestry ('Sar-Nur'), North African ancestry (using either 'Morocco-EN', 'Morocco-LN', 'Guanche' as proxies), "Eastern Mediterranean" ancestry (using 'Anatolia-BA', 'Canaanite', 'Iran-CA', 'Levant-BA', 'Minoan-BA', or 'Myc-BA' as proxies), "Northern Mediterranean" ancestry ('Balkans-BA', 'CE-EBA', 'CE-LBA', 'Iberia-BA', or 'Italy-North-Bk' as proxies). To explore models of continuity from the Nuragic period in more detail, we ran analyses with and without the Nuragic period as an outgroup. This is motivated by the suggestion that post-Nuragic Sardinia can be modelled without including Nuragic Sardinia as a source[68]. Also as the PCA projection of these samples (main Fig. 2) reveals substantial heterogeneity among individuals from these post-Nuragic sites, we ran individual-level qpAdm analyses. Throughout this individual qpAdm analysis, we used the `allsnps=YES` option of qpAdm to increase power for individuals with low coverage, with the trade-off of potential challenges to comparison among tests because of varying number and type of SNPs included in each test (in preliminary analyses, we find inferred admixture proportions are relatively stable but p-values often shift to lower values when using `allsnps=YES`, in particular when groups with only HO SNPs are included).

Below we present tables summarizing useful candidate 3-way admixture models that emerge from systematically fitting a large space of possible admixture models (see Supp. Data 3 for the full space of models explored and the results). To present a summary of the results, we rejected all models with p-values less than 10^{-3}, and of the remainder, we report for each individual: 1) the model with Sar-Nur as a source and the highest p-Value ('Max-p' rows in the tables below) 2) The model with the highest Sar-Nur ancestry ('Nur-

Target	Source	p-value[1]	p-value[2]	SNPs[1]	SNPs[2]
Sar-MSR	Sar-Nur	$4.2 \cdot 10^{-10}$	$1.3 \cdot 10^{-8}$	311261	265627
Sar-VIL	Sar-Nur	$5.5 \cdot 10^{-38}$	$2.4 \cdot 10^{-30}$	394972	343884
Sar-ORC002	Sar-Nur	0.226	0.073	424950	373524
Sar-AMC	Sar-Nur	$2.7 \cdot 10^{-11}$	$1.0 \cdot 10^{-9}$	254844	213495
Sar-COR	Sar-Nur	$4.4 \cdot 10^{-4}$	0.024	457014	404713
Sar-SNN	Sar-Nur	$2.0 \cdot 10^{-13}$	$1.2 \cdot 10^{-7}$	302404	256854
Sar-MSR	Sar-VIL	$1.8 \cdot 10^{-6}$	$3.2 \cdot 10^{-7}$	363484	334199
Sar-ORC002	Sar-VIL	$3.6 \cdot 10^{-16}$	$1.5 \cdot 10^{-13}$	490495	460180
Sar-AMC	Sar-VIL	$9.5 \cdot 10^{-4}$	$3.0 \cdot 10^{-6}$	297830	270102
Sar-COR	Sar-VIL	$1.1 \cdot 10^{-13}$	$6.0 \cdot 10^{-12}$	508448	476109
Sar-SNN	Sar-VIL	$5.5 \cdot 10^{-7}$	$3.5 \cdot 10^{-4}$	352048	322138
Sar-ORC002	Sar-MSR	$6.2 \cdot 10^{-5}$	$7.0 \cdot 10^{-3}$	416033	392989
Sar-ORC002	Sar-SNN	$1.4 \cdot 10^{-7}$	$6.6 \cdot 10^{-4}$	400469	375910
Sar-ORC002	Sar-COR	0.022	0.07	580348	554053
Sar-SNN	Sar-COR	$2.3 \cdot 10^{-3}$	0.053	405668	378408
Sar-COR	Sar-MSR	$7.9 \cdot 10^{-4}$	0.012	420850	394801
Sar-AMC	Sar-MSR	0.203	0.149	259383	236390
Sar-SNN	Sar-MSR	0.037	0.416	302518	277650
Sar-COR	Sar-AMC	0.014	0.012	342468	317509
Sar-SNN	Sar-AMC	0.124	0.132	252533	228651
Cagliari	Sar-MSR	0.078	0.132	91175	48680
Cagliari	Sar-VIL	$9.1 \cdot 10^{-12}$	$5.3 \cdot 10^{-10}$	105181	57732
Cagliari	Sar-ORC002	$4.3 \cdot 10^{-3}$	0.053	108792	60273
Cagliari	Sar-AMC	0.012	0.012	80661	42191
Cagliari	Sar-COR	0.16	0.783	112525	62893
Cagliari	Sar-SNN	0.037	0.249	90795	48421
Ogliastra	Sar-MSR	0.044	0.067	95649	53793
Ogliastra	Sar-VIL	$8.6 \cdot 10^{-14}$	$6.8 \cdot 10^{-12}$	111202	64492
Ogliastra	Sar-ORC002	0.023	0.113	115415	67701
Ogliastra	Sar-AMC	$2.2 \cdot 10^{-3}$	$1.4 \cdot 10^{-3}$	84175	46237
Ogliastra	Sar-COR	0.261	0.922	119654	70844
Ogliastra	Sar-SNN	0.016	0.115	95088	53320

Supplementary Table 6: Support for models of continuity between two populations inferred by qpAdm. Upper 2 blocks: Models for continuity between ancient Sardinian groups. Lower 2 blocks: Models for continuity between ancient and present-day Sardinia. We test two outgroups: The standard set A15 ([1]), and also A15 with Morocco-EN added as additional outgroup ([2]) to test for differential ancient North African ancestry. We also show the number of intersecting SNPs used in the qpAdm analysis.

High'), 3) The model with the lowest Sar-Nur ancestry ('Nur-Low'), 4) The model with the highest p-value for Nuragic as outgroup ('Nur-Out').

While we show models with the highest p-values for parts 1 and 4, we caution that ranking models by p-values in this form is highly susceptible to sampling error and pitfalls of multiple testing (recall that under the null, p-values are uniformly distributed between 0 and 1). Moreover, the p-value of a qpAdm analysis reflects the power to reject a model, and this depends on the number of available SNPs included in the qpAdm analysis which can vary substantially between possible source populations and targets. Power is also affected by choice of outgroups. Thus, these results should not be taken as model selection or ranking procedure, but rather as a tool to systematically show one model that "fits" the data among what is often many, and potential admixture fractions under these oversimplifying models.

As shown in Tables 7 and 8, a few consistent results emerge:

- In the models with ‘Sar-Nur’ ancestry allowed, the “Eastern Mediterranean” ancestry and “Northern Mediterranean” ancestries appear in substantial fractions in fitting models in all post-Nuragic samples, except the Villamar samples (‘VIL’ samples), which instead are fit as containing ‘Sar-Nur’, ‘North African’, and ’Eastern Mediterranean’ ancestry.

- The amounts of ‘Sar-Nur’ ancestry can vary widely among fitting models, and for most sites, the individuals can be also fit using ‘Sar-Nur’ as an outgroup.

- An exception is the Corona Moltana samples from the Medieval period. These samples fit the highest amount of ’Sar-Nur’ ancestry, and these models produce no valid models with ‘Sar-Nur’ as an outgroup.

- Another exception is the Medieval period ‘SNN-001’ sample which appears from the PCA (main Fig. 2) to be of a broadly Iberian origin. Based on the PCA, it seems outside the space that is possible to model with the sources available to us. We hypothesize this Medieval period sample is from mainland Iberia.

	Source Populations					Admixture Fractions			Standard Error		
**Target**	**A**	**B**	**C**	**Model**	**p-Value**	**A**	**B**	**C**	**A**	**B**	**C**
VIL004	Sar-Nur	Guanche	Minoan-BA	Max-p	0.964	0.281	0.26	0.459	0.125	0.069	0.121
VIL004	Sar-Nur	Morocco-EN	Iran-CA	Nur-High	0.248	0.667	0.174	0.158	0.063	0.079	0.073
VIL004	Sar-Nur	Morocco-LN	Anatolia-BA	Nur-Low	0.904	0.029	0.855	0.116	0.162	0.289	0.158
VIL004	Guanche	Minoan-BA	Iberia-BA	Nur-Out	0.954	0.253	0.557	0.19	0.074	0.089	0.091
VIL006	Sar-Nur	Morocco-LN	Levant-BA	Max-p	0.171	0.126	0.786	0.088	0.047	0.068	0.045
VIL006	Sar-Nur	Morocco-EN	Levant-BA	Nur-High	$1.0 \cdot 10^{-3}$	0.553	0.228	0.219	0.048	0.031	0.062
VIL006	Sar-Nur	Morocco-LN	Myc-BA	Nur-Low	0.084	0.075	0.732	0.193	0.061	0.138	0.175
VIL006	Morocco-LN	Levant-BA	Italy-North-Bk	Nur-Out	0.131	0.807	0.028	0.164	0.065	0.042	0.055
VIL007	Sar-Nur	Guanche	Canaanite	Max-p	0.878	0.467	0.211	0.322	0.046	0.054	0.075
VIL007	Sar-Nur	Morocco-EN	Iran-CA	Nur-High	0.037	0.653	0.196	0.151	0.031	0.031	0.04
VIL007	Sar-Nur	Morocco-LN	Italy-North-Bk	Nur-Low	0.162	0.038	0.711	0.251	0.124	0.064	0.147
VIL007	Morocco-EN	Minoan-BA	Iberia-BA	Nur-Out	0.699	0.22	0.471	0.309	0.023	0.045	0.046
VIL009	Sar-Nur	Morocco-EN	Anatolia-BA	Max-p	0.699	0.175	0.254	0.571	0.092	0.05	0.11
VIL009	Sar-Nur	Morocco-EN	Iran-CA	Nur-High	0.134	0.481	0.205	0.314	0.05	0.064	0.072
VIL009	Sar-Nur	Guanche	Minoan-BA	Nur-Low	0.183	0.042	0.411	0.547	0.126	0.063	0.121
VIL009	Guanche	Anatolia-BA	Iberia-BA	Nur-Out	0.177	0.287	0.616	0.098	0.071	0.098	0.084
VIL010	Sar-Nur	Guanche	Levant-BA	Max-p	0.339	0.396	0.337	0.266	0.063	0.061	0.087
VIL010	Sar-Nur	Morocco-EN	CE-LBA	Nur-High	$7.0 \cdot 10^{-3}$	0.622	0.361	0.018	0.05	0.036	0.056
VIL010	Sar-Nur	Morocco-LN	CE-EBA	Nur-Low	0.17	0.126	0.787	0.087	0.063	0.061	0.039
VIL010	Morocco-LN	Levant-BA	Iberia-BA	Nur-Out	0.649	0.639	0.167	0.194	0.09	0.065	0.047
VIL011	Sar-Nur	Guanche	Myc-BA	Max-p	0.315	0.123	0.382	0.494	0.072	0.065	0.122
VIL011	Sar-Nur	Guanche	Iran-CA	Nur-High	0.015	0.404	0.567	0.029	0.041	0.06	0.051
VIL011	Sar-Nur	Morocco-LN	Myc-BA	Nur-Low	0.025	0.007	0.681	0.311	0.046	0.154	0.18
VIL011	Guanche	Minoan-BA	Italy-North-Bk	Nur-Out	0.361	0.497	0.245	0.258	0.046	0.085	0.094
MSR002	Sar-Nur	Myc-BA	CE-LBA	Max-p	0.046	0.108	0.817	0.075	0.062	0.065	0.035
MSR002	Sar-Nur	Morocco-LN	Iran-CA	Nur-High	$1.0 \cdot 10^{-3}$	0.542	0.175	0.284	0.142	0.216	0.084
MSR002	Sar-Nur	Minoan-BA	CE-LBA	Nur-Low	$6.6 \cdot 10^{-3}$	0.005	0.63	0.365	0.104	0.078	0.051
MSR002	Morocco-EN	Myc-BA	Iberia-BA	Nur-Out	0.071	0.038	0.839	0.123	0.027	0.049	0.04
MSR003	Sar-Nur	Myc-BA	CE-EBA	Max-p	0.965	0.164	0.652	0.184	0.065	0.076	0.04
MSR003	Sar-Nur	Morocco-EN	Iran-CA	Nur-High	$6.1 \cdot 10^{-3}$	0.729	0.04	0.231	0.031	0.032	0.043
MSR003	Sar-Nur	Iran-CA	Balkans-BA	Nur-Low	0.809	0.0	0.123	0.877	0.165	0.045	0.195
MSR003	Morocco-LN	Anatolia-BA	Italy-North-Bk	Nur-Out	0.753	0.243	0.171	0.586	0.127	0.091	0.088
AMC001	Sar-Nur	Morocco-LN	Iran-CA	Max-p	0.961	0.111	0.692	0.197	0.057	0.099	0.055
AMC001	Sar-Nur	Morocco-EN	Iran-CA	Nur-High	0.087	0.431	0.134	0.435	0.04	0.05	0.058
AMC001	Sar-Nur	Canaanite	Iberia-BA	Nur-Low	0.523	0.002	0.804	0.194	0.203	0.063	0.182
AMC001	Morocco-EN	Anatolia-BA	CE-EBA	Nur-Out	0.939	0.146	0.773	0.081	0.046	0.059	0.054
AMC005	Sar-Nur	Iran-CA	CE-EBA	Max-p	0.278	0.723	0.256	0.021	0.064	0.048	0.073
AMC005	Sar-Nur	Iran-CA	Iberia-BA	Nur-High	0.276	0.731	0.262	0.007	0.172	0.042	0.167
AMC005	Sar-Nur	Levant-BA	Italy-North-Bk	Nur-Low	0.06	0.049	0.276	0.675	2.332	0.27	2.592
AMC005	Morocco-LN	Myc-BA	Iberia-BA	Nur-Out	0.052	0.243	0.587	0.17	0.609	0.59	0.065
AMC014	Sar-Nur	Levant-BA	Italy-North-Bk	Max-p	0.959	0.172	0.183	0.645	0.188	0.063	0.223
AMC014	Sar-Nur	Morocco-EN	Iberia-BA	Nur-High	0.032	0.835	0.148	0.017	0.138	0.027	0.148
AMC014	Sar-Nur	Anatolia-BA	Italy-North-Bk	Nur-Low	0.85	0.06	0.214	0.727	0.183	0.097	0.237
AMC014	Morocco-LN	Minoan-BA	Italy-North-Bk	Nur-Out	0.45	0.293	0.049	0.658	0.15	0.07	0.141
ORC002	Sar-Nur	Guanche	Minoan-BA	Max-p	0.367	0.838	0.058	0.103	0.071	0.03	0.077
ORC002	Sar-Nur	Morocco-EN	CE-LBA	Nur-High	0.135	0.963	0.038	-0.001	0.042	0.022	0.049
ORC002	Sar-Nur	Minoan-BA	Italy-North-Bk	Nur-Low	0.324	0.628	0.107	0.265	0.189	0.079	0.212
ORC002	-	-	-	Nur-Out	-	-	-	-	-	-	-

Supplementary Table 7: Individual qpAdm Models for the Phoenician and Punic sites of Monte Sirai and Villamar ('MSR' and 'VIL' samples), the Imperial Roman era site Alghero Monte Carru ('AMC' samples) and the single post-Nuragic individual from S'Orcu 'e Tueri ('ORC'). We report for each individual: 1) The model with Sar-Nur as a source that had the highest p-Value ('Max-p' rows in the tables below) 2) The model with the highest Sar-Nur ancestry ('Nur-High'), 3) The model with the lowest Sar-Nur ancestry ('Nur-Low'), 4) The model with the highest p-value for Sar-Nur as outgroup ('Nur-Out'). See text for further discussion and caveats.

	Source Populations					Admixture Fractions			Standard Error		
Target	A	B	C	Model	p-Value	A	B	C	A	B	C
COR001	Sar-Nur	Iran-CA	CE-EBA	Max-p	0.766	0.788	0.152	0.06	0.043	0.035	0.049
COR001	Sar-Nur	Morocco-EN	CE-LBA	Nur-High	0.047	0.858	0.064	0.078	0.041	0.023	0.05
COR001	Sar-Nur	Minoan-BA	Italy-North-Bk	Nur-Low	0.068	0.185	0.115	0.699	0.221	0.074	0.215
COR001	-	-	-	Nur-Out	-	-	-	-	-	-	-
COR002	Sar-Nur	Minoan-BA	CE-EBA	Max-p	0.775	0.578	0.2	0.221	0.087	0.069	0.043
COR002	Sar-Nur	Morocco-EN	Levant-BA	Nur-High	0.013	0.878	0.035	0.087	0.045	0.026	0.059
COR002	Sar-Nur	Minoan-BA	Iberia-BA	Nur-Low	0.283	0.044	0.364	0.592	0.218	0.091	0.146
COR002	-	-	-	Nur-Out	-	-	-	-	-	-	-
SNN001[1]	Basque	-	-	Max-p	0.016	-	-	-	-	-	-
SNN001[1]	Basque	-	-	Nur-High	0.016	-	-	-	-	-	-
SNN001[1]	Basque	-	-	Nur-Low	0.016	-	-	-	-	-	-
SNN001[1]	Basque	-	-	Nur-Out	0.021	-	-	-	-	-	-
SNN002	Sar-Nur	Morocco-EN	CE-LBA	Max-p	0.227	0.302	0.165	0.533	0.056	0.043	0.074
SNN002	Sar-Nur	Morocco-EN	CE-LBA	Nur-High	0.227	0.302	0.165	0.533	0.056	0.043	0.074
SNN002	Sar-Nur	Morocco-LN	CE-LBA	Nur-Low	0.149	0.106	0.462	0.432	0.07	0.122	0.082
SNN002	Morocco-EN	Iran-CA	Iberia-BA	Nur-Out	0.109	0.189	0.119	0.692	0.046	0.049	0.04
SNN003	Sar-Nur	Morocco-LN	Balkans-BA	Max-p	0.819	0.082	0.489	0.429	0.1	0.079	0.13
SNN003	Sar-Nur	Morocco-EN	Iran-CA	Nur-High	0.073	0.729	0.084	0.187	0.031	0.031	0.039
SNN003	Sar-Nur	Levant-BA	Balkans-BA	Nur-Low	0.418	0.072	0.248	0.68	0.141	0.054	0.16
SNN003	Morocco-LN	Anatolia-BA	Italy-North-Bk	Nur-Out	0.713	0.428	0.043	0.529	0.106	0.075	0.082
SNN004	Sar-Nur	Levant-BA	Balkans-BA	Max-p	0.848	0.095	0.267	0.638	0.177	0.062	0.201
SNN004	Sar-Nur	Morocco-EN	CE-LBA	Nur-High	$2.5 \cdot 10^{-3}$	0.752	0.159	0.089	0.052	0.038	0.06
SNN004	Sar-Nur	Anatolia-BA	Italy-North-Bk	Nur-Low	0.069	0.007	0.3	0.693	0.349	0.105	0.409
SNN004	Morocco-LN	Myc-BA	Iberia-BA	Nur-Out	0.33	0.101	0.722	0.177	0.284	0.274	0.058

Supplementary Table 8: Individual qpAdm Models for the Medieval individuals of Corona Moltana and the San Nicola Necropoli. We report for each individual: 1) The model with Sar-Nur as a source that had the highest p-Value ('Max-p' rows in the tables below) 2) The model with the highest Sar-Nur ancestry ('Nur-High'), 3) The model with the lowest Sar-Nur ancestry ('Nur-Low'), 4) The model with the highest p-value for Sar-Nur as outgroup ('Nur-Out'). See text for further discussion and caveats. [1]: For SNN001 we could not find any viable models with ancient sources. Therefore, we additionally tested modern HO populations as one-way source. Basque provided the best fit (p=0.016), followed by Spanish (p=$4. \cdot 10^{-4}$) and French (p=$6.7 \cdot 10^{-5}$).

	Source Populations					Admixture Fractions			Standard Error		
Target	A	B	C	Model	p-Value	A	B	C	A	B	C
Olb1	Sar-Nur	Canaanite	CE-LBA	Max-p	0.047	0.53	0.317	0.153	0.054	0.047	0.046
Olb1	Sar-Nur	Morocco-EN	Iran-CA	Nur-High	$2.1 \cdot 10^{-3}$	0.735	0.058	0.207	0.031	0.029	0.038
Olb1	Sar-Nur	Morocco-LN	Iberia-BA	Nur-Low	$3.0 \cdot 10^{-3}$	0.076	0.784	0.14	0.244	0.144	0.116
Olb1	-	-	-	Nur-Out	-	-	-	-	-	-	-
Olb2	Sar-Nur	Canaanite	CE-EBA	Max-p	0.046	0.531	0.201	0.268	0.052	0.046	0.046
Olb2	Sar-Nur	Iran-CA	CE-LBA	Nur-High	$3.9 \cdot 10^{-3}$	0.635	0.129	0.236	0.044	0.036	0.051
Olb2	Sar-Nur	Canaanite	Iberia-BA	Nur-Low	$8.3 \cdot 10^{-3}$	0.091	0.309	0.601	0.143	0.043	0.135
Olb2	-	-	-	Nur-Out	-	-	-	-	-	-	-
Sas1	Sar-Nur	Minoan-BA	CE-LBA	Max-p	0.041	0.485	0.317	0.198	0.08	0.064	0.047
Sas1	Sar-Nur	Morocco-EN	Iran-CA	Nur-High	$4.6 \cdot 10^{-3}$	0.826	0.017	0.157	0.03	0.027	0.039
Sas1	Sar-Nur	Minoan-BA	Iberia-BA	Nur-Low	$1.2 \cdot 10^{-3}$	0.155	0.423	0.422	0.289	0.091	0.217
Sas1	-	-	-	Nur-Out	-	-	-	-	-	-	-
Sas2	Sar-Nur	Canaanite	CE-LBA	Max-p	0.32	0.523	0.178	0.299	0.052	0.045	0.048
Sas2	Sar-Nur	Morocco-EN	CE-LBA	Nur-High	0.012	0.639	0.054	0.307	0.044	0.027	0.053
Sas2	Sar-Nur	Morocco-LN	Balkans-BA	Nur-Low	$1.9 \cdot 10^{-3}$	0.072	0.265	0.663	0.138	0.152	0.239
Sas2	-	-	-	Nur-Out	-	-	-	-	-	-	-
Nuo1	Sar-Nur	Canaanite	CE-EBA	Max-p	0.153	0.656	0.185	0.159	0.05	0.05	0.049
Nuo1	Sar-Nur	Morocco-EN	Iran-CA	Nur-High	0.029	0.806	0.023	0.171	0.031	0.029	0.039
Nuo1	Sar-Nur	Guanche	Italy-North-Bk	Nur-Low	$7.4 \cdot 10^{-3}$	0.054	0.045	0.901	0.51	0.084	0.585
Nuo1	Guanche	Minoan-BA	Iberia-BA	Nur-Out	$2.3 \cdot 10^{-3}$	0.039	0.298	0.663	0.035	0.057	0.054
Nuo2	Sar-Nur	Canaanite	CE-LBA	Max-p	0.102	0.66	0.207	0.132	0.052	0.046	0.049
Nuo2	Sar-Nur	Morocco-EN	Iran-CA	Nur-High	$8.4 \cdot 10^{-3}$	0.815	0.033	0.151	0.032	0.03	0.044
Nuo2	Sar-Nur	Anatolia-BA	Italy-North-Bk	Nur-Low	$3.9 \cdot 10^{-3}$	0.018	0.091	0.89	12.133	3.151	15.274
Nuo2	-	-	-	Nur-Out	-	-	-	-	-	-	-
Ogl1	Sar-Nur	Canaanite	CE-LBA	Max-p	0.019	0.588	0.216	0.196	0.05	0.045	0.05
Ogl1	Sar-Nur	Iran-CA	CE-LBA	Nur-High	$2.2 \cdot 10^{-3}$	0.686	0.147	0.167	0.045	0.035	0.055
Ogl1	Sar-Nur	Canaanite	Balkans-BA	Nur-Low	$1.3 \cdot 10^{-3}$	0.345	0.205	0.45	0.163	0.055	0.195
Ogl1	-	-	-	Nur-Out	-	-	-	-	-	-	-
Ogl2	Sar-Nur	Guanche	CE-LBA	Max-p	0.076	0.717	0.165	0.118	0.047	0.035	0.05
Ogl2	Sar-Nur	Morocco-EN	Anatolia-BA	Nur-High	$1.9 \cdot 10^{-3}$	0.85	0.122	0.028	0.063	0.025	0.072
Ogl2	Sar-Nur	Morocco-LN	Balkans-BA	Nur-Low	$2.2 \cdot 10^{-3}$	0.462	0.449	0.09	0.112	0.104	0.137
Ogl2	-	-	-	Nur-Out	-	-	-	-	-	-	-
Ori1	Sar-Nur	Canaanite	CE-LBA	Max-p	0.171	0.612	0.21	0.178	0.053	0.047	0.051
Ori1	Sar-Nur	Morocco-EN	Iran-CA	Nur-High	0.015	0.789	0.044	0.167	0.031	0.028	0.04
Ori1	Sar-Nur	Myc-BA	Iberia-BA	Nur-Low	$3.4 \cdot 10^{-3}$	0.065	0.528	0.407	0.184	0.104	0.124
Ori1	Guanche	Minoan-BA	Iberia-BA	Nur-Out	$2.1 \cdot 10^{-3}$	0.046	0.328	0.626	0.034	0.055	0.051
Ori2	Sar-Nur	Guanche	Minoan-BA	Max-p	0.239	0.699	0.107	0.194	0.064	0.036	0.069
Ori2	Sar-Nur	Morocco-EN	CE-LBA	Nur-High	$2.2 \cdot 10^{-3}$	0.908	0.069	0.023	0.045	0.028	0.052
Ori2	Sar-Nur	Minoan-BA	Iberia-BA	Nur-Low	0.174	0.262	0.381	0.357	0.224	0.085	0.159
Ori2	Guanche	Minoan-BA	Iberia-BA	Nur-Out	0.04	0.02	0.428	0.552	0.038	0.052	0.046
Cam1	Sar-Nur	Canaanite	CE-EBA	Max-p	0.039	0.524	0.272	0.203	0.052	0.046	0.047
Cam1	Sar-Nur	Iran-CA	CE-LBA	Nur-High	$1.7 \cdot 10^{-3}$	0.656	0.198	0.146	0.044	0.038	0.052
Cam1	Sar-Nur	Morocco-LN	Balkans-BA	Nur-Low	$4.1 \cdot 10^{-3}$	0.102	0.51	0.387	0.101	0.112	0.149
Cam1	-	-	-	Nur-Out	-	-	-	-	-	-	-
Cam2	Sar-Nur	Canaanite	CE-LBA	Max-p	0.374	0.575	0.307	0.118	0.053	0.045	0.044
Cam2	Sar-Nur	Iran-CA	Balkans-BA	Nur-High	$7.6 \cdot 10^{-3}$	0.777	0.222	0.001	0.169	0.043	0.196
Cam2	Sar-Nur	Morocco-LN	Italy-North-Bk	Nur-Low	$5.8 \cdot 10^{-3}$	0.039	0.361	0.6	0.339	0.174	0.485
Cam2	-	-	-	Nur-Out	-	-	-	-	-	-	-
Cag1	Sar-Nur	Guanche	Myc-BA	Max-p	$5.0 \cdot 10^{-3}$	0.443	0.171	0.386	0.125	0.042	0.15
Cag1	Sar-Nur	Guanche	Iran-CA	Nur-High	$4.4 \cdot 10^{-3}$	0.713	0.174	0.112	0.035	0.047	0.045
Cag1	Sar-Nur	Morocco-LN	Balkans-BA	Nur-Low	$1.8 \cdot 10^{-3}$	0.256	0.65	0.094	0.111	0.086	0.113
Cag1	-	-	-	Nur-Out	-	-	-	-	-	-	-
Cag2	Sar-Nur	Canaanite	CE-LBA	Max-p	0.02	0.602	0.242	0.156	0.05	0.044	0.045
Cag2	Sar-Nur	Canaanite	CE-EBA	Nur-High	0.019	0.604	0.237	0.159	0.05	0.045	0.046
Cag2	Sar-Nur	Myc-BA	CE-LBA	Nur-Low	$9.1 \cdot 10^{-3}$	0.341	0.502	0.157	0.086	0.092	0.04
Cag2	-	-	-	Nur-Out	-	-	-	-	-	-	-
Car1	Sar-Nur	Canaanite	CE-LBA	Max-p	0.083	0.517	0.346	0.137	0.05	0.048	0.046
Car1	Sar-Nur	Morocco-EN	Iran-CA	Nur-High	$6.1 \cdot 10^{-3}$	0.719	0.053	0.228	0.029	0.029	0.035
Car1	Sar-Nur	Morocco-LN	Myc-BA	Nur-Low	$1.2 \cdot 10^{-3}$	0.228	0.359	0.413	0.067	0.219	0.236
Car1	-	-	-	Nur-Out	-	-	-	-	-	-	-
Car2	Sar-Nur	Canaanite	CE-EBA	Max-p	$5.8 \cdot 10^{-3}$	0.592	0.255	0.153	0.053	0.047	0.051
Car2	Sar-Nur	Canaanite	CE-EBA	Nur-High	$5.8 \cdot 10^{-3}$	0.592	0.255	0.153	0.053	0.047	0.051
Car2	Sar-Nur	Myc-BA	CE-EBA	Nur-Low	$1.1 \cdot 10^{-3}$	0.301	0.566	0.133	0.107	0.122	0.045
Car2	-	-	-	Nur-Out	-	-	-	-	-	-	-

Supplementary Table 9: Individual qpAdm Models for 2 randomly selected present-day individuals per province. See Supp. Table 8 for explanation of table headers.

Supplementary Note 6: Population Structure Models

Joseph H. Marcus and Tyler A. Joseph

Here we describe extended results applying variants of the Pritchard, Stephens, and Donnelly model[69] to the dataset of ancient individuals and modern individuals from west Eurasia and north Africa.

ADMIXTURE

We applied `ADMIXTURE` to a joint dataset of ancient and modern samples, as described in the Materials and Methods[70]. In (Fig. 19, Fig. 20) we display a gallery plot, i.e. the typical stacked bar plot for $K = 2$ through 8. For each K, we run 5 replicates of `ADMIXTURE` and plot the the runs reaching the highest log likelihood.

DyStruct

We compared the results from `ADMIXTURE` to a time-aware population structure model: `DyStruct`[71]. `DyStruct` implements a novel variational inference algorithm based on the Pritchard, Stephens, Donnelly model[69] that incorporates fluctuations in allele frequencies due to differences in sample times. Specifically, `DyStruct` defines a normal approximation to genetic drift that serves as a prior for allele frequency estimates for different time points. At each time point the model is equivalent to the PSD model, but allele frequency estimates between time points are regularized by the prior to ensure allele frequencies estimated from samples nearby in time are closer than allele frequencies from samples further apart. This corrects for genetic drift in populations between samples, potentially leading to different conclusions than `ADMIXTURE`.

We applied `DyStruct` to an un-normalized genotype matrix of ancient and modern samples. Sample times were converted to generation times assuming a 25 year generation time, and provided as input to `DyStruct`. We used default prior settings and set the effective population size hyper-parameter to 15000. (Fig. 21, Fig. 22) displays the fitted admixture coefficients for $K = 2$ to $K = 8$. Qualitatively, `DyStruct` appears to place emphasis on explaining modern populations as mixtures of ancient populations by assigning singular clusters to ancient samples, and describing modern samples as mixtures of these ancient clusters. Hence, ancient samples in `DyStruct` appear as more "extreme" versions of their cluster assignments in `ADMIXTURE`. Consequently, estimates of the genetic contribution from ancient samples into modern populations are different between both models. For instance, modern Sardinian individuals in `DyStruct` appear to inherit a larger fraction of early European farmer ancestry, Steppe/EHG ancestry instead of WHG ancestry, and a smaller portion of shared ancestry from Neolithic Iran and Neolithic Levant.

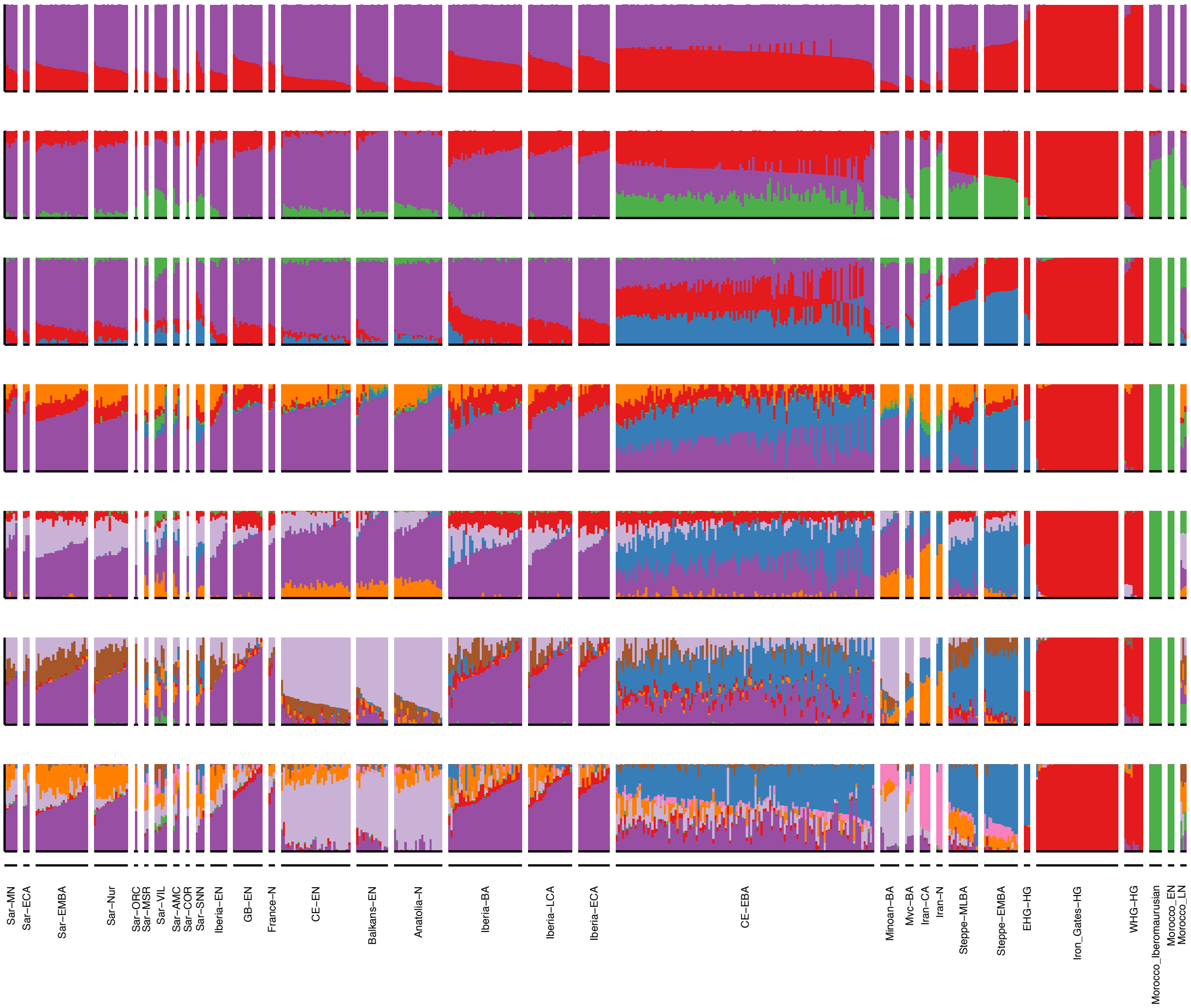

Supplementary Figure 19: Visualization of admixture coefficients estimated by ADMIXTURE for a subset of ancient individuals. Each row corresponds to the highest likelihood run of five replicates of ADMIXTURE on both ancient and modern individuals from $K = 2$ to $K = 8$. Here we display admixture coefficients for just a subset ancient individuals.

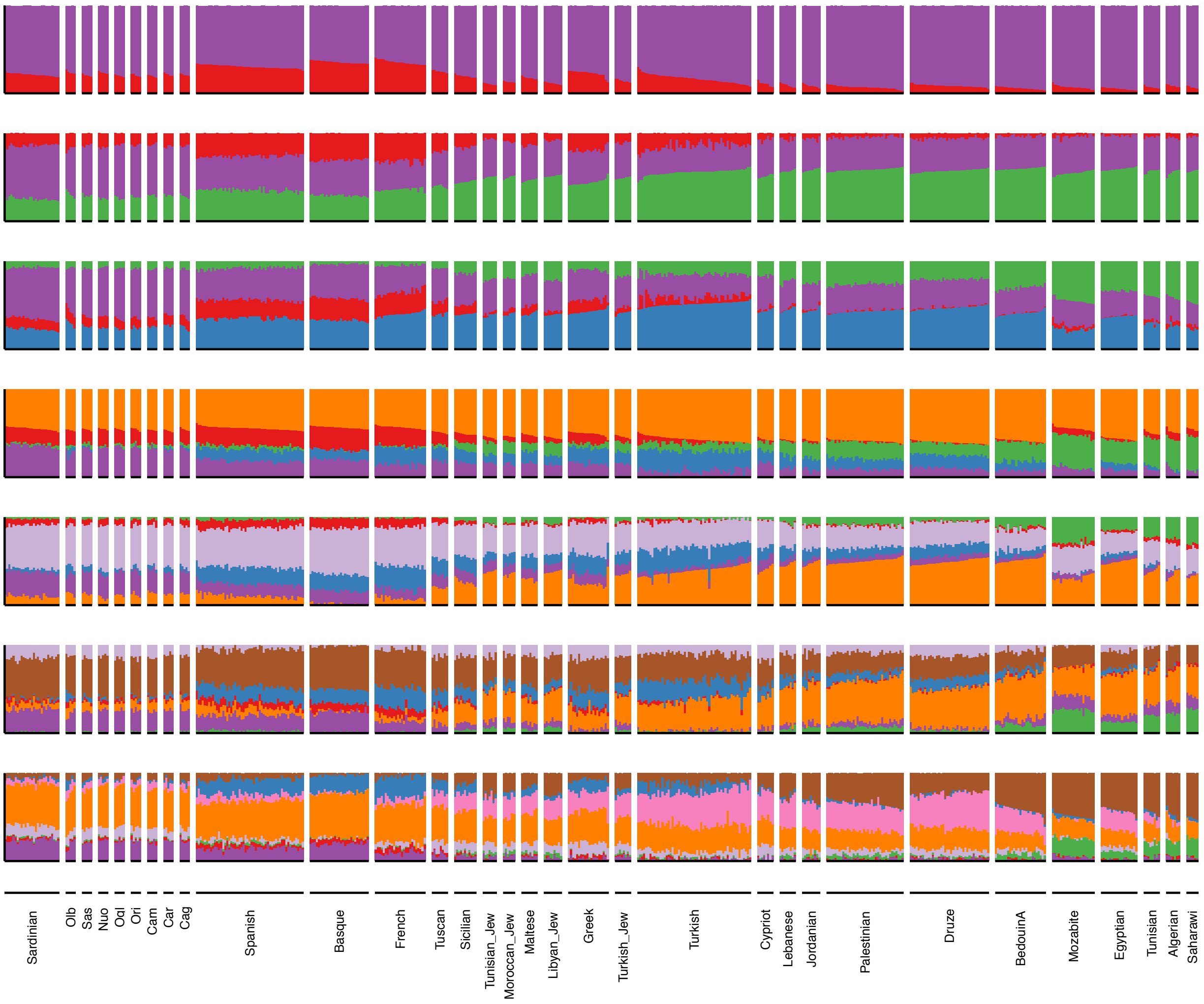

Supplementary Figure 20: Visualization of admixture coefficients estimated by ADMIXTURE for a subset of modern individuals. Each row corresponds to the highest likelihood run of five replicates of ADMIXTURE on both ancient and modern individuals from $K = 2$ to $K = 8$. Here we display admixture coefficients for just a subset modern individuals.

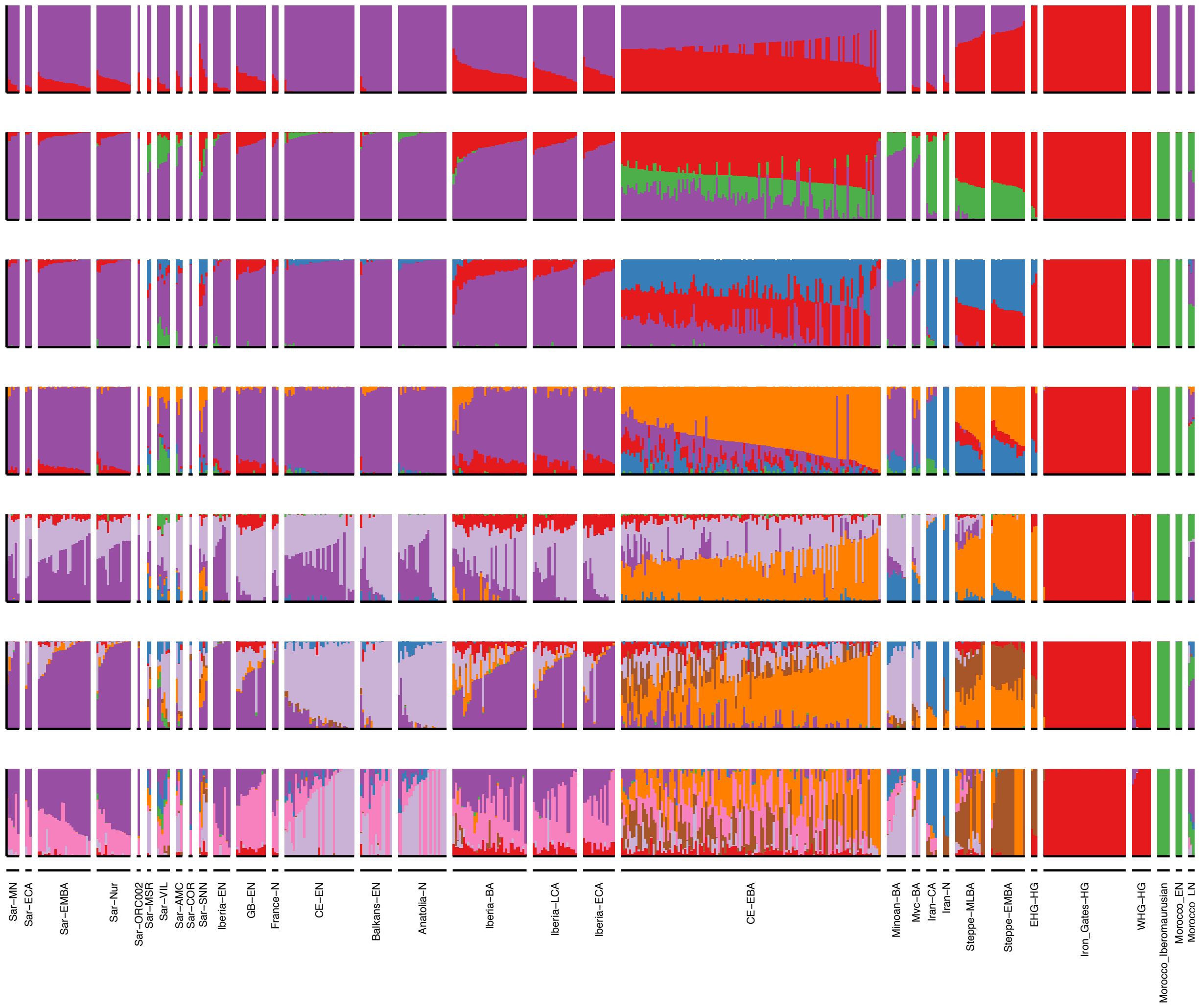

Supplementary Figure 21: Visualization of admixture coefficients estimated by DyStruct on a subset of ancient individuals. Each row corresponds to a run of DyStruct on both ancient and modern individuals from $K = 2$ to $K = 8$. Here we display admixture coefficients for a subset ancient individuals.

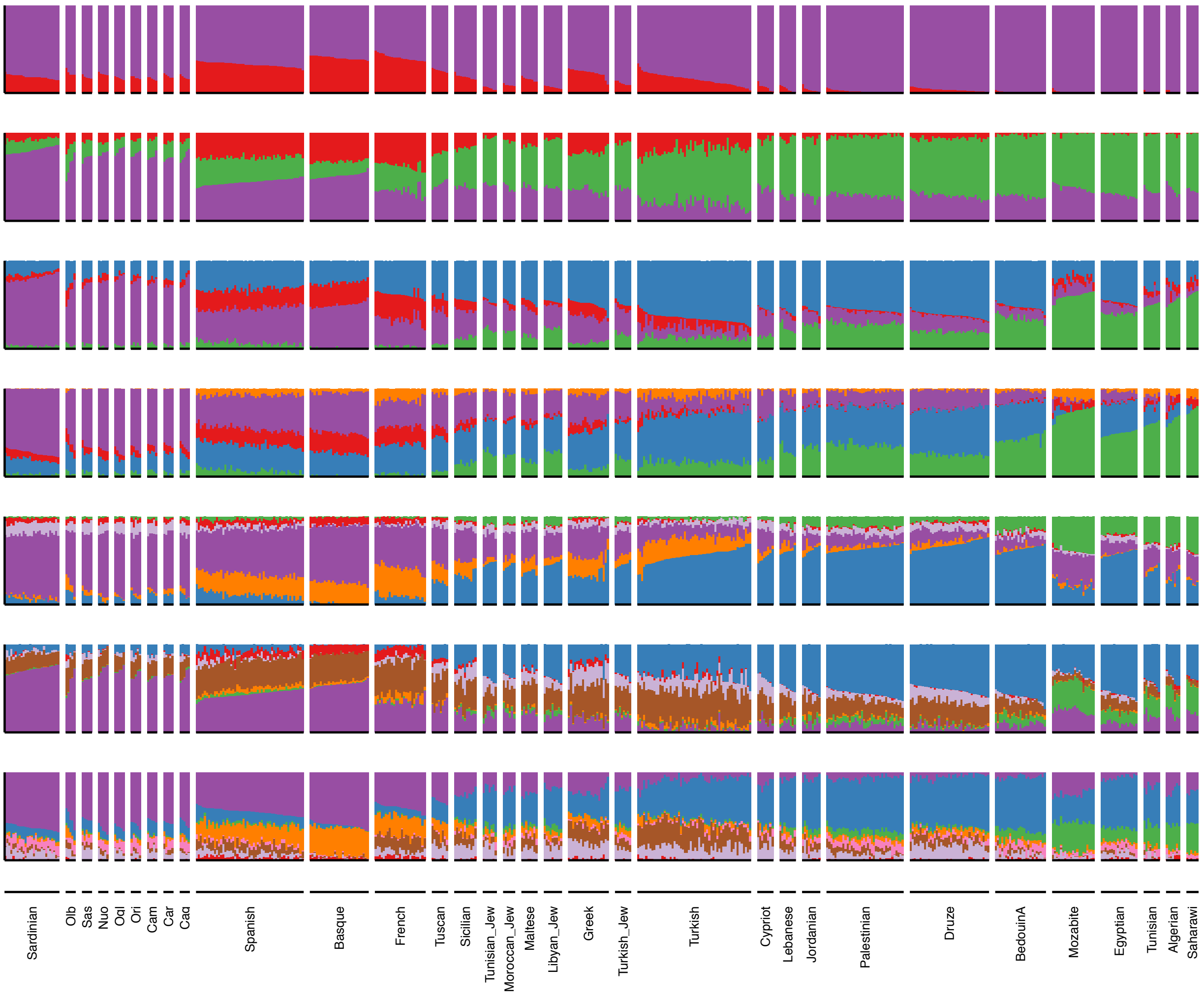

Supplementary Figure 22: Visualization of admixture coefficients estimated by DyStruct on a subset of modern individuals. Each row corresponds to a run of DyStruct on both ancient and modern individuals from $K = 2$ to $K = 8$. Here we display admixture coefficients for a subset of modern individuals.

Supplementary Note 7: Shrinkage correction in PC score prediction

Joseph Marcus

In Fig. 2 of the main text, we perform principal components analysis (PCA) on contemporary west Eurasian and north African individuals and project each ancient individual onto modern PCs, one at a time, by solving a simple least squares problem. It is known that the estimated principal scores are biased and exhibit a regression towards the mean effect (shrinkage towards 0 if the data is mean centered) for high dimensional data i.e. when the number of features (SNPs) is much greater than the number of samples (individuals)[72–74]. To correct for this shrinkage effect when predicting PC scores for out of sample individuals, we implemented a shrinkage correction factor through a jackknife resampling approach proposed previously[72] (for computational experiments see `https://github.com/jhmarcus/pcshrink/blob/master/notebook/patterson-example.ipynb`).

The procedure was performed through the following steps: (1) We compute a rank-K truncated SVD on the full dataset to obtain a first set of uncorrected PC scores. (2) We remove each individual from the dataset and compute a rank-K truncated SVD on the remaining individuals (3) We project the held-out individual on to the PCA computed from the dataset of step (2). Using the eigenvectors computed for each individual, we then constructed a jackknife estimator of the bias. We then applied this correction factor to the ancient individuals' PC scores to create our final visualization. For comparison we also applied two correction procedures, "shrinkmode" and "autoshrink", implemented in `smartpca`[75]. In Supp. Fig. 23, we see no major qualitative differences between the corrected ancient PC scores for the three correction approaches.

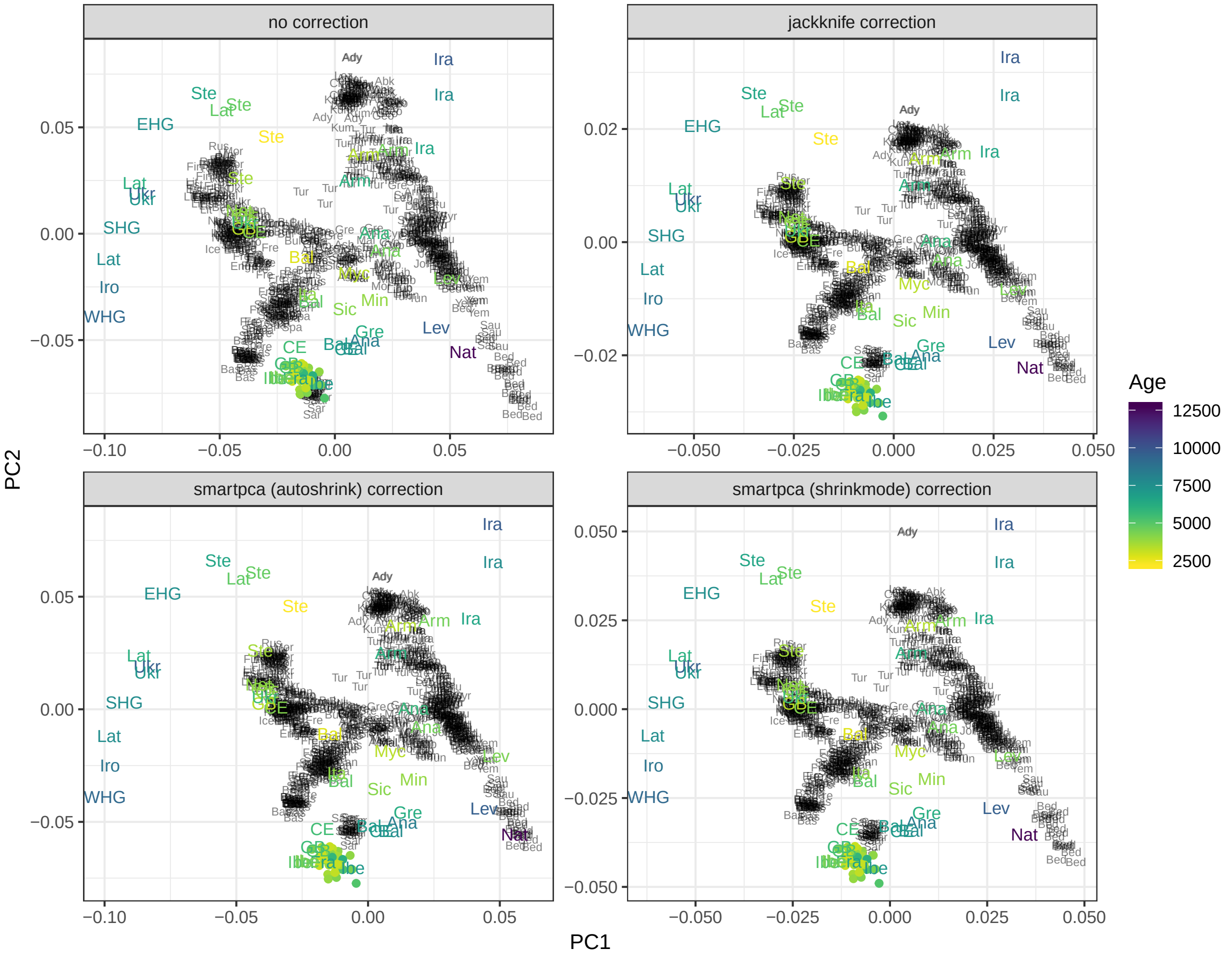

Supplementary Figure 23: Visualization of the effect of different shrinkage correction approaches on the top two PCs for projected ancient individuals. All panels show the results an initial PCA on modern Western Eurasian individuals, each of whom are represented as a black three-letter short hand for their assigned population label. We project each ancient individual onto these modern PCs and then represent the median projected PC value of each ancient group as a three-letter short hand colored by the group's median age. Each panel shows a different different correction approach (in the top left showing no correction). We do not observe substantial differences between the three correction approaches especially in the region around the ancient Sardinian individuals from this study.

Supplementary Note 8: Assessing Geographic Substructure in Pre-Nuragic Ancient Sardinia

Joseph Marcus and John Novembre

In our initial analysis we saw no strong signal of sub-population structure in ancient Sardinian individuals until the post-Nurgaic period when we observed heterogeneous shifts in ancestry depending on the sampled archaeological site (main Fig. 2). Here we investigate in more detail if we can detect a signal of geographic substructure before the Nurgaic period.

One approach to investigate signals of fine-scale geographic structure would be to take the genotypes of pre-Nuragic ancient Sardinian individuals and directly visualize their covariance structure, in a low dimensional space, such as typically done with Principal Components Analysis (PCA). Unfortunately, our ancient capture data has high levels of missingness. For instance, the median proportion of missing sites across pairs individuals is 0.862 with the 5th and 95th percentiles being 0.422 and 0.989. These high levels of missingness are problematic for two reasons: 1) There are not many widely used methods that account for missing data, while estimating population structure. 2) Interpreting the resulting structure could be difficult as pairs of individuals have unequal levels of overlapping data, creating a heteroskedastic noise model. As a compromise, we use the projections of ancient Sardinian individuals onto PCs trained on modern Sardinian individuals and tried to see if these PCs were associated with any covariates related to geography or sampling location. As mentioned previously, using the projections onto modern Principal Components is a powerful approach because there is very little missingness in the moderns genotypes and they are not affected by sequencing error modes unique to ancient DNA. This means projecting the ancient genotypes onto modern PCs helps to, in some sense, regularize the estimates of population structure for the ancients. As a drawback, we note that this approach could potentially miss population structure present in the ancients but absent in the moderns.

In linear models individually regressing latitude and longitude against the top 9 PC projections we observe that only the projection onto PC6 of the within-Sardinia variation is significantly associated with longitude (Figure 28, Figure 29). We also subdivided each ancient individual into broad geographic regions based on the locations of archaeological sites with more than 3 sampled individuals (CentralEast1, CentralEast2, NorthWest1, NorthWest2, SouthWest, and the remainder were put into a group labelled '< 3'). We computed 1 way anovas of each PC projection vs these regional labels and found that only the projection onto PC6 of the within-Sardinia variation was significantly associated with these course geographic labels (Figure 30). Finally, we regressed the PC projections against the radio-carbon date age estimate of each individual and found again that only PC6 was significantly associated with age. Because age is confounded with longitude and the course geographic region it is difficult to determine which covariate is driving the association with PC6, although we note the association with age is much more significant than the other covariates. Furthermore, correcting for multiple testing, the associations with PC6 would likely not survive. Figures 32 and 33 contain plots of each of the PCs for reference.

In summary, at least from the projections on to PCs derived from modern data, we could not detect any strong and significant signals of geographic structure. Higher coverage sequencing data and perhaps haplotype based approaches to reveal signals of geographic structure for individuals with such low levels of differentiation.

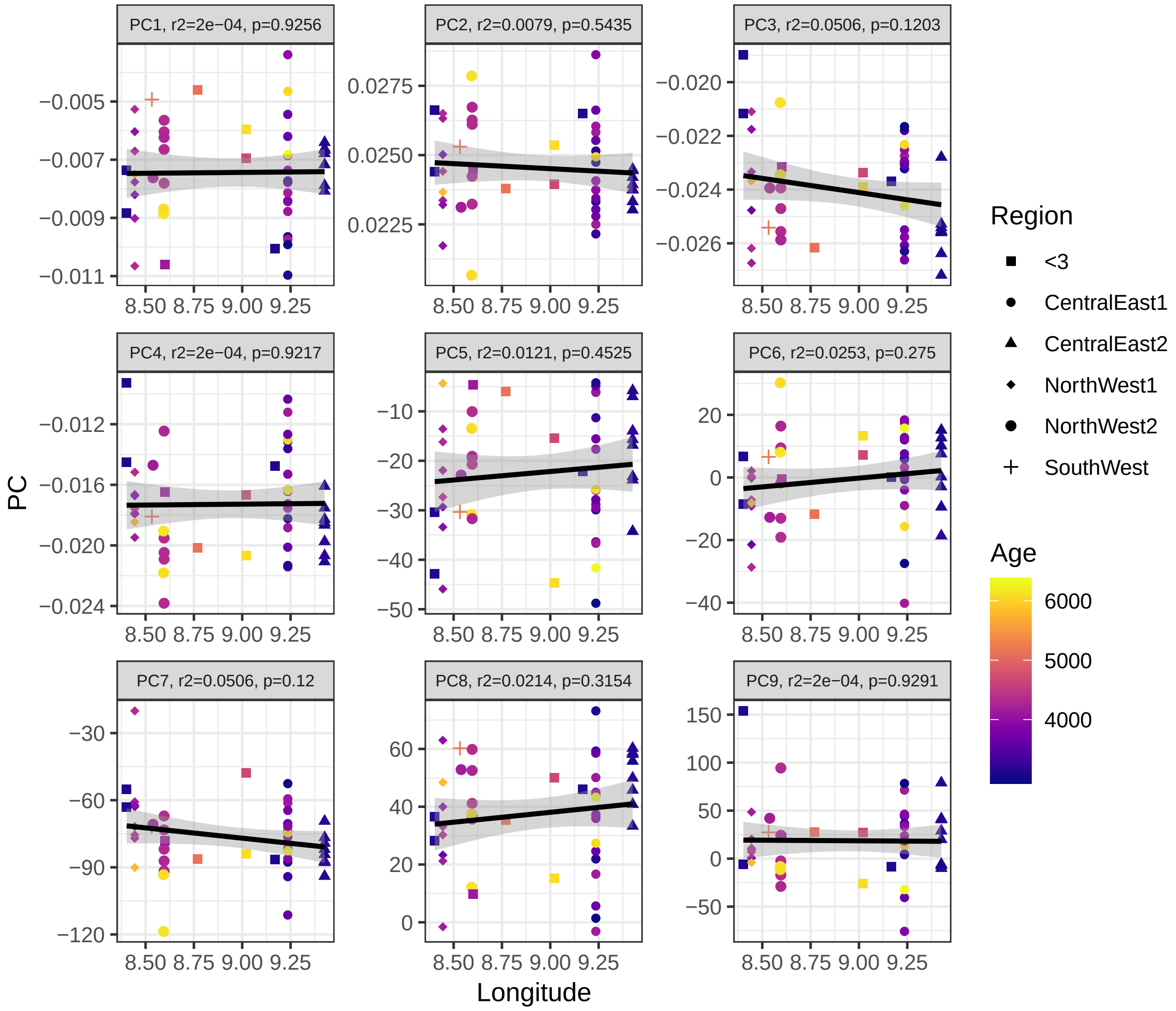

Supplementary Figure 24: Main Text Figure 2B Principal Component Projections vs. Longitude: Here each sub-panel displays a visualization of a given PC vs Longitude, with its corresponding fitted linear regression. We show a grid of the top 9 PCs. The text in each sub-panel displays the sample correlation and significance of association between each PC and longitude. PC6 is the only PC that has a significant association.

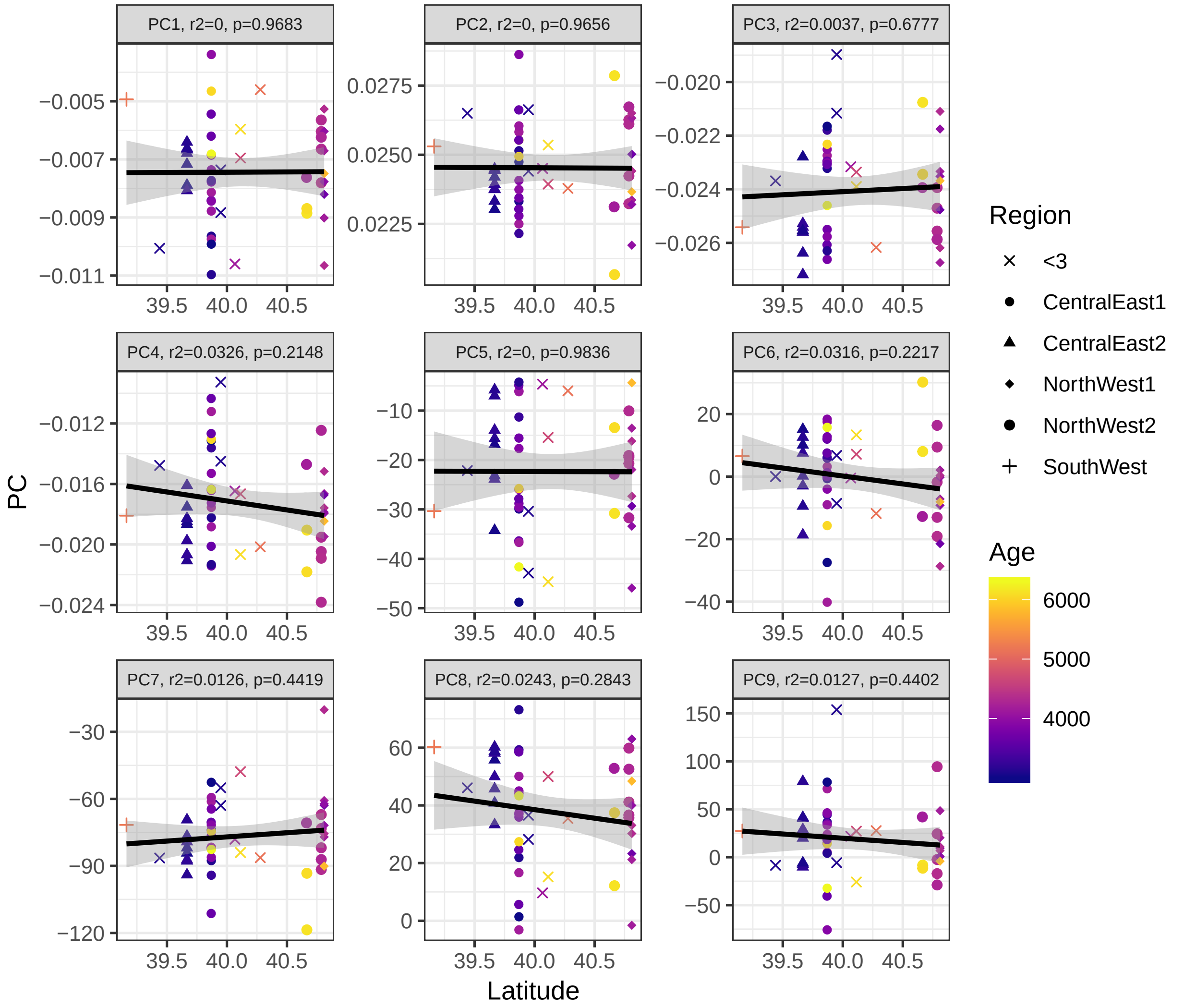

Supplementary Figure 25: Main Text Figure 2B Principal Component Projections vs. Latitude: Here each sub-panel displays a visualization of a given PC vs Latitude, with its corresponding fitted linear regression. We show a grid of the top 9 PCs. The text in each sub-panel displays the sample correlation and significance of association between each PC and Latitude.

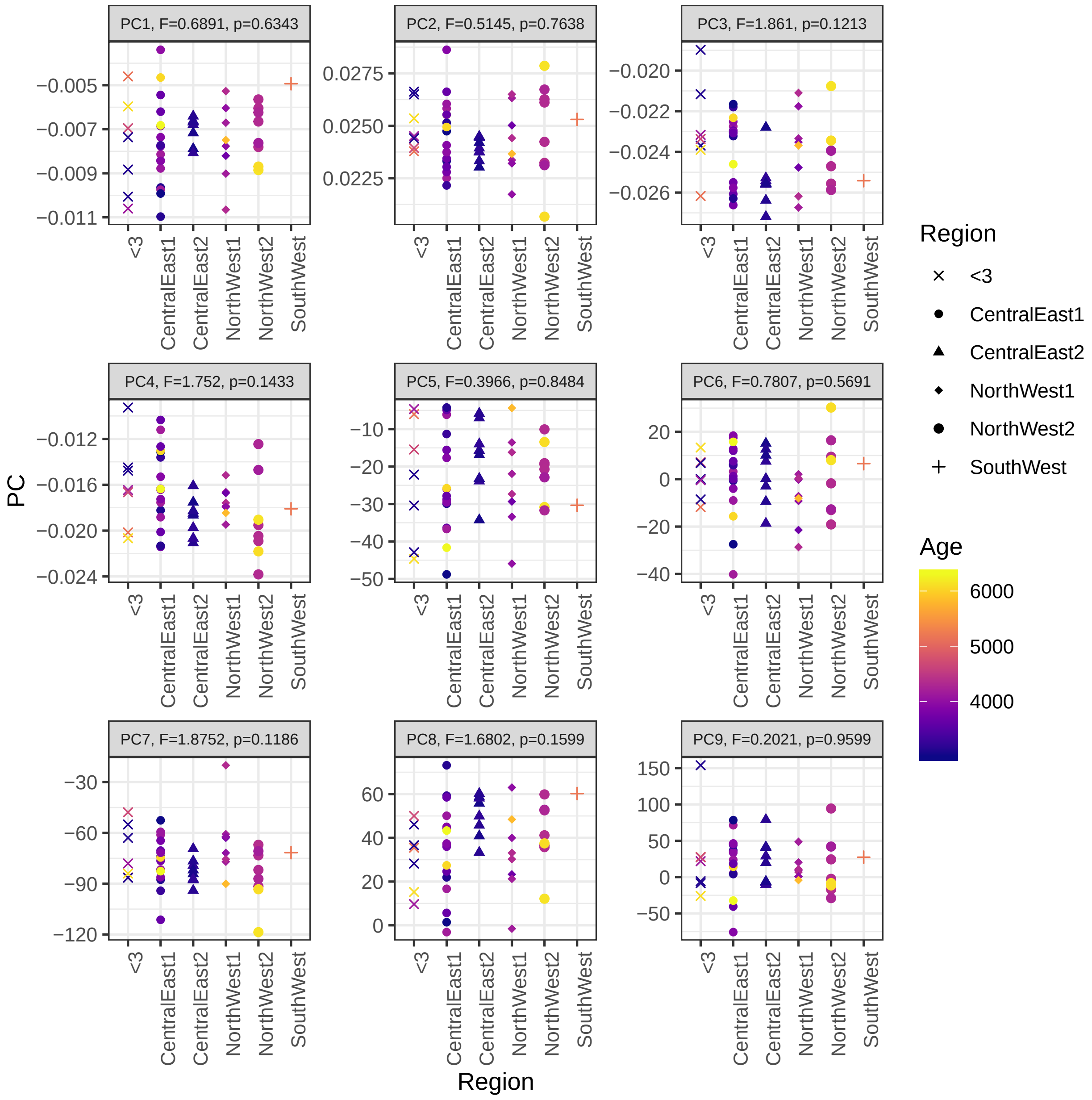

Supplementary Figure 26: Main Text Figure 2B Principal Component Projections vs. Region: Here each sub-panel displays a visualization of a given PC vs a course geographic region label. We assigned individuals to the ”<3” label if less than three individuals were sampled at the same geographic position. We show a grid of the top 9 PCs. The text in each sub-panel displays the F statistic and p-value output by computing a one-way anova.

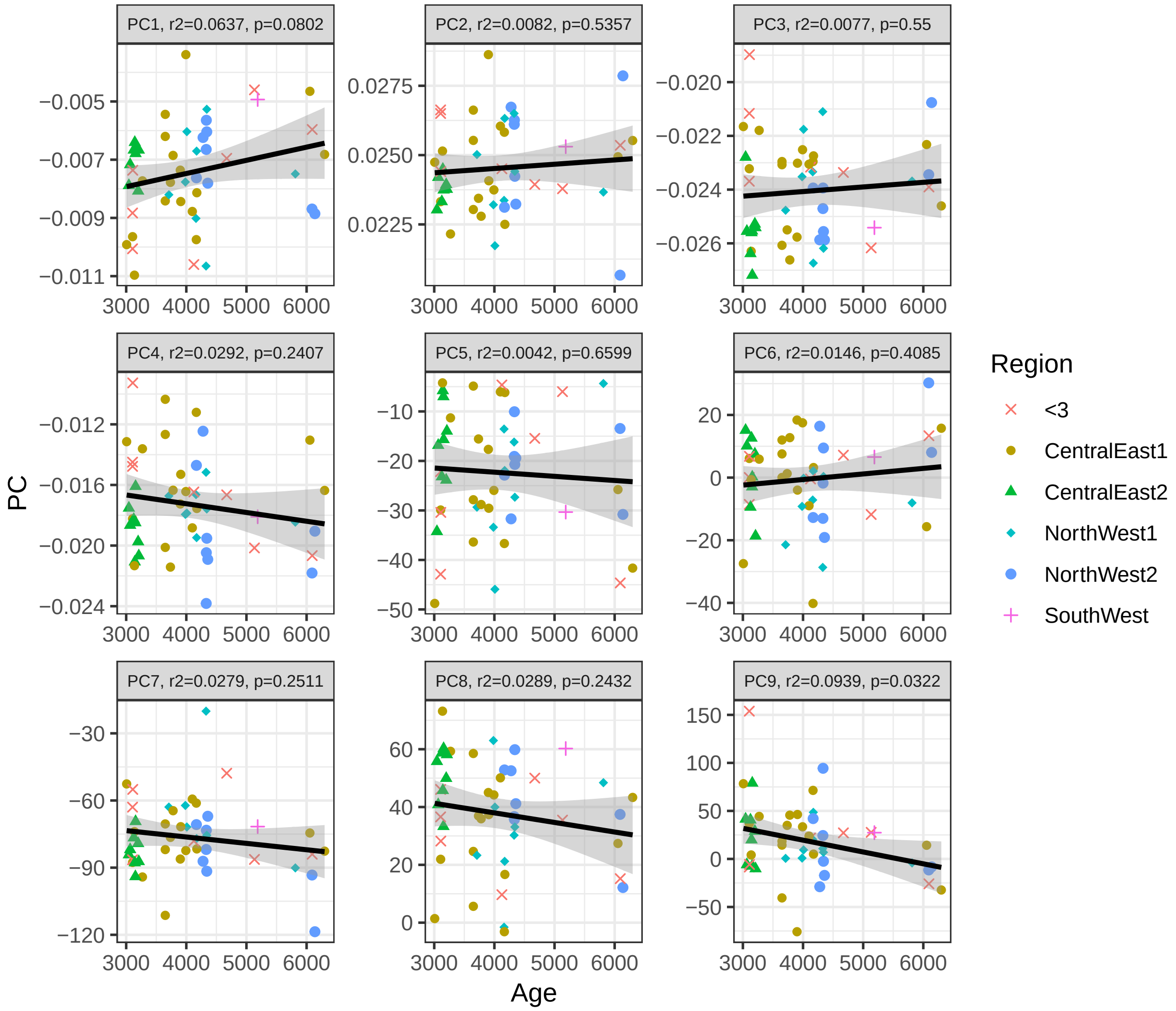

Supplementary Figure 27: Main Text Figure 2B Principal Component Projections vs. Age: Here each sub-panel displays a visualization of a given PC vs Age, with its corresponding fitted linear regression. We show a grid of the top 9 PCs. The text in each sub-panel displays the sample correlation and significance of association between each PC and age.

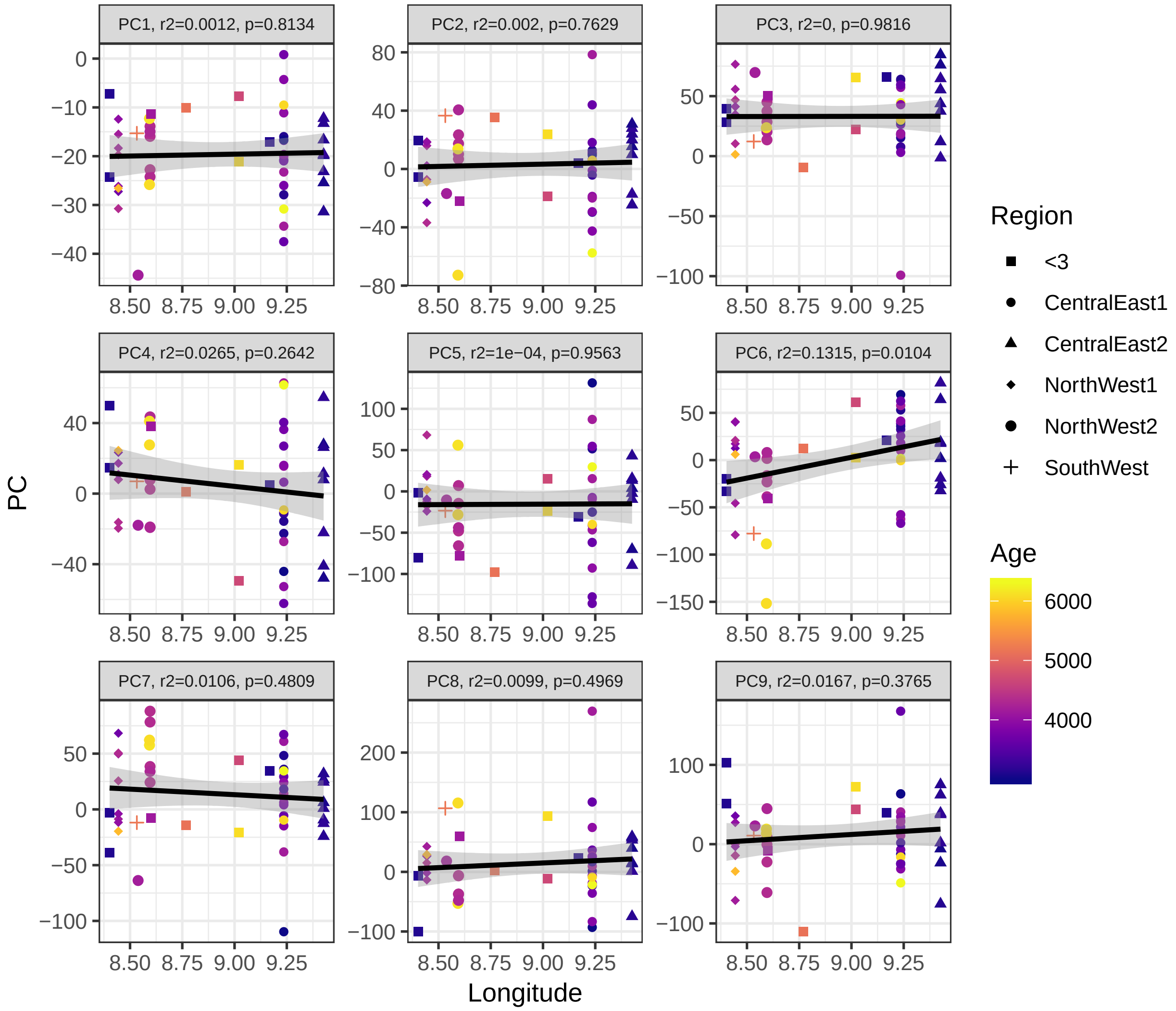

Supplementary Figure 28: Sardinia Principal Component Projections vs. Longitude: Here each sub-panel displays a visualization of a given PC vs Longitude, with its corresponding fitted linear regression. We show a grid of the top 9 PCs. The text in each sub-panel displays the sample correlation and significance of association between each PC and longitude. PC6 is the only PC that has a significant association.

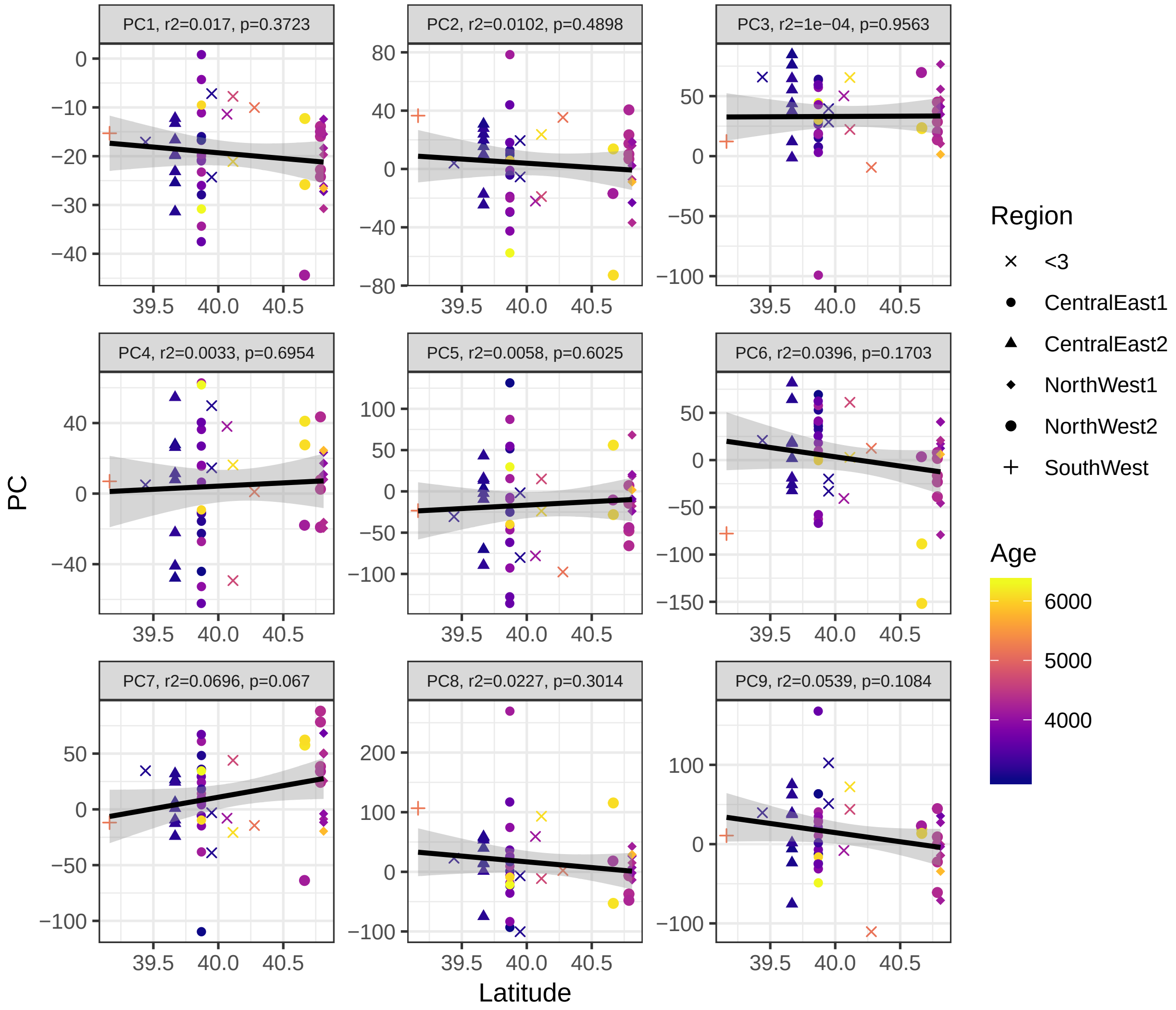

Supplementary Figure 29: Sardinia Principal Component Projections vs. Latitude: Here each sub-panel displays a visualization of a given PC vs Latitude, with its corresponding fitted linear regression. We show a grid of the top 9 PCs. The text in each sub-panel displays the sample correlation and significance of association between each PC and Latitude. There are no PCs significantly associated with latitude.

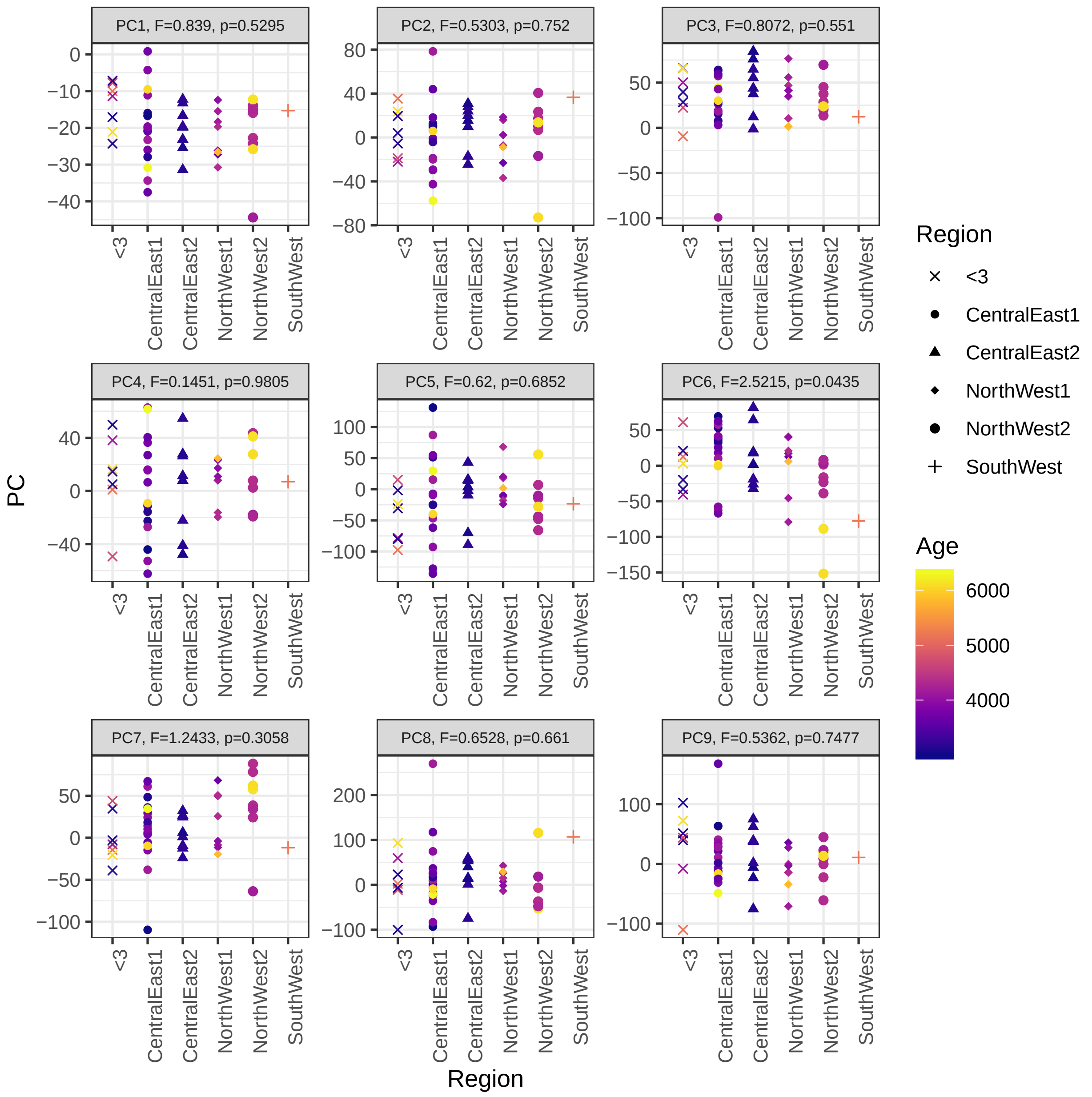

Supplementary Figure 30: Sardinia Principal Component Projections vs. Region: Here each sub-panel displays a visualization of a given PC vs a course geographic region label. We assigned individuals to the ”<3” label if less than three individuals were sampled at the same geographic position. We show a grid of the top 9 PCs. The text in each sub-panel displays the F statistic and p-value output by computing a one-way anova. PC6 is the only PC significantly associated with these course geographic labels.

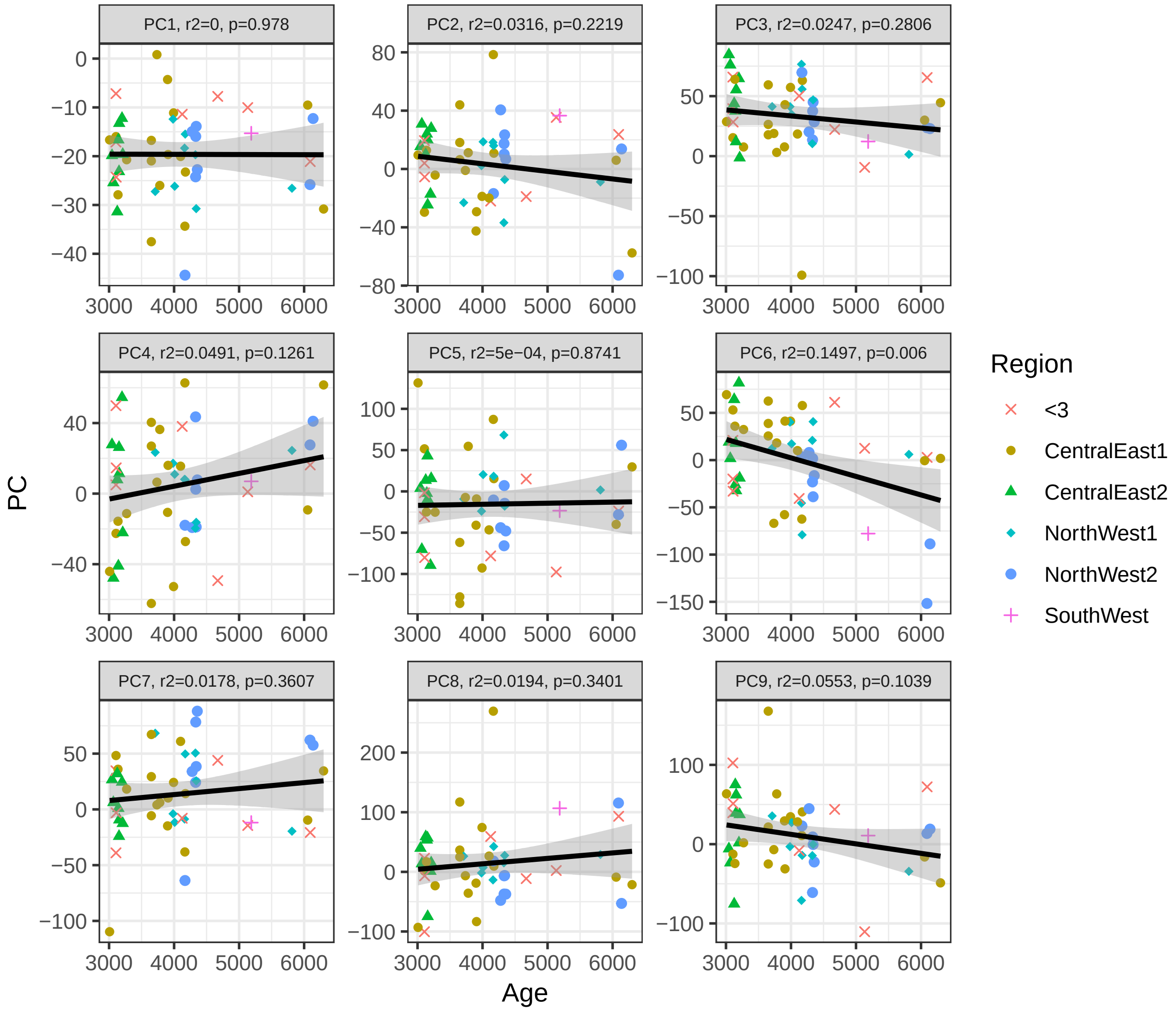

Supplementary Figure 31: Sardinia Principal Component Projections vs. Age: Here each sub-panel displays a visualization of a given PC vs Age, with its corresponding fitted linear regression. We show a grid of the top 9 PCs. The text in each sub-panel displays the sample correlation and significance of association between each PC and age. PC6 is the only PC significantly associated with age.

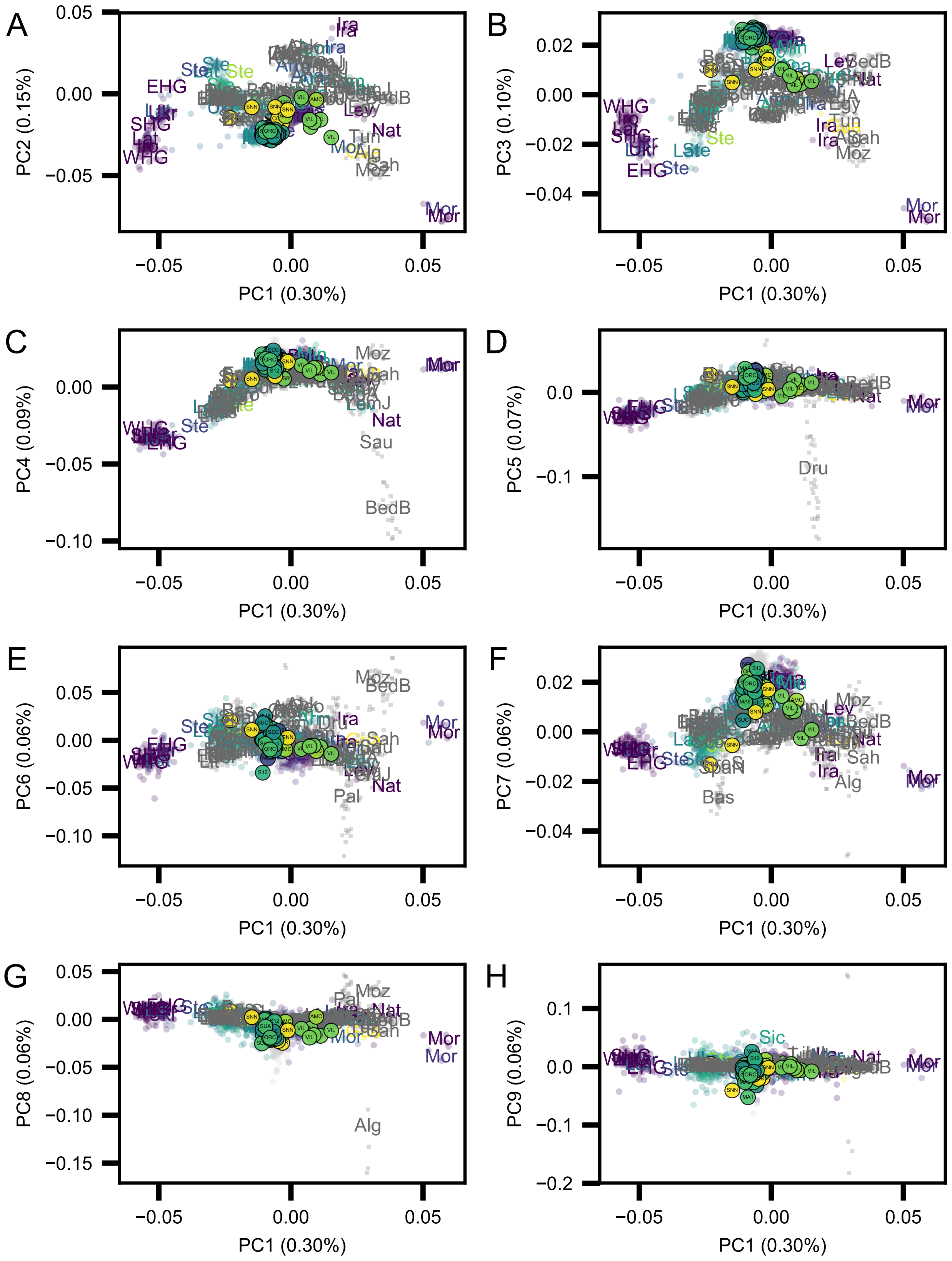

Supplementary Figure 32: Principal components 1-10 for the PCA shown in Main Text Fig. 2. Each subplot has PC1 on its x-Axis. Label and color choices are described in detail in the caption of Main Fig. 2.

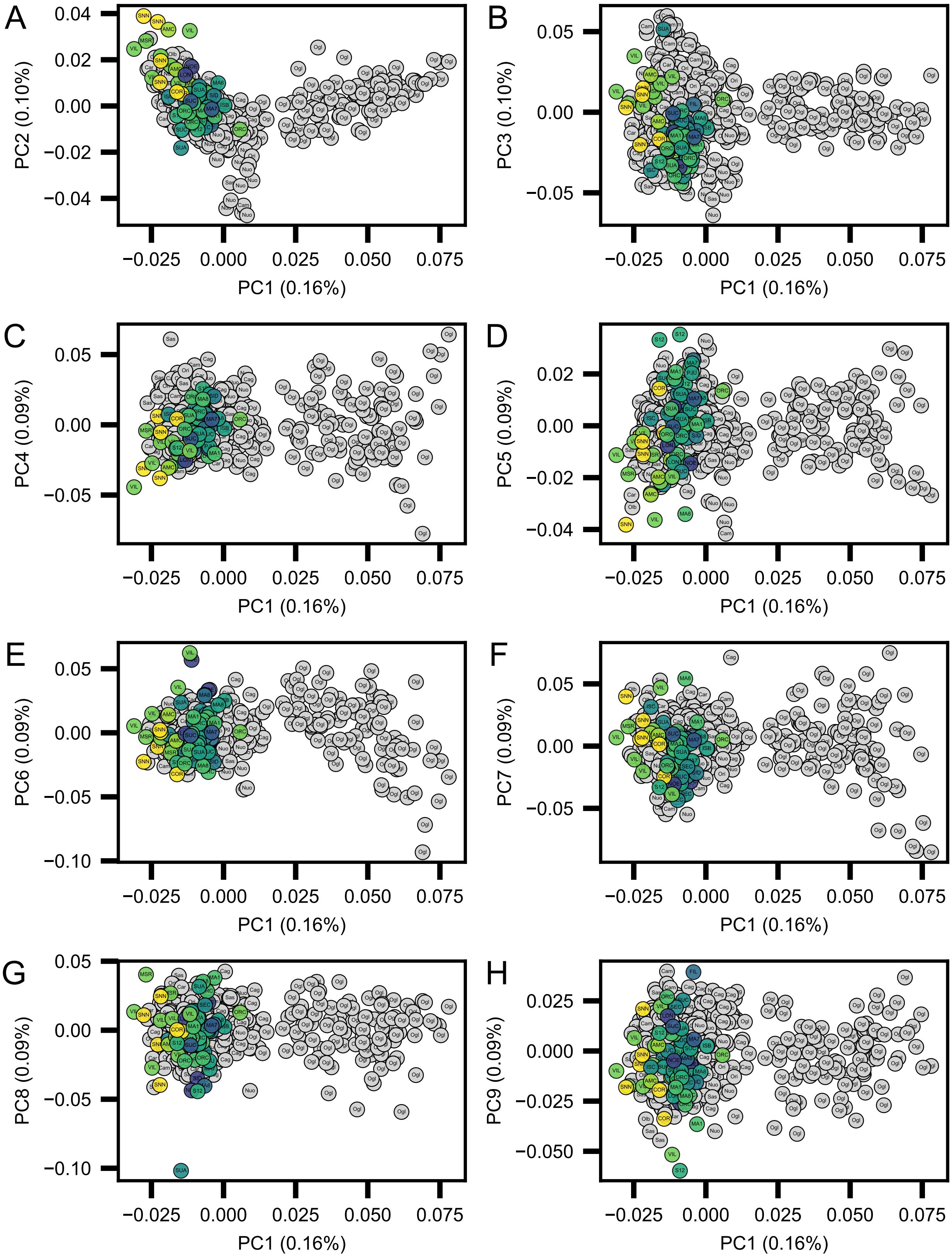

Supplementary Figure 33: Principal components 1-10 for the PCA shown in Main Fig. 4B. Each subplot has PC1 on its x-Axis. Label and color choices are described in detail in the caption of Main Fig. 4B.

References

[1] Germanà, F. I paleosardi di Is Aruttas (Cabras-Oristano). nota I. *Archivio per l'Antropologia e l'Etnologia* **109-110**, 343–391 (1980).

[2] Germanà, F. I paleosardi di Is Aruttas (Cabras-Oristano). nota II. *Archivio per l'Antropologia e l'Etnologia* **120**, 233–280 (1982).

[3] Germanà, F. *L'uomo in Sardegna dal paleolitico all'età nuragica* (Carlo Delfino, Sassari, 1995).

[4] Martella, P., Floris, R. & Usai, E. Primi dati osteologici su resti scheletrici provenienti da due tombe della Sardegna meridionale: Ingurtosu Mannu (Donori) e Sa Serra Masì (Siliqua). *Annali dell'Università di Ferrara, Museologia Scientifica e Naturalistica* **10**, 68–73 (2014).

[5] Canci, A. *et al.* A case of Madelung's Deformity in a skeleton from Nuragic Sardinia. *International Journal of Osteoarchaeology* **12**, 173–177 (2002).

[6] Santoni, V. & Usai, L. Domus de janas in località Cannas di Sotto (Carbonia). In Santoni, V. (ed.) *Carbonia e il Sulcis: archeologia e territorio*, 53–82 (S'alvure, Oristano, 1995).

[7] Lai, L. Il clima nella Sardegna preistorica e protostorica: problemi e nuove prospettive. In *Atti della XLIV Riunione Scientifica dell'Istituto Italiano di Preistoria e Protostoria, Cagliari, Barumini, Sassari, 23-28 November, 2009, vol. I – Relazioni generali*, 313–324 (Istituto Italiano di Preistoria e Protostoria, 2009).

[8] Salis, G., Farci, F., Sarigu, M. & Pusceddu, V. Necropoli di Cannas di Sotto, Carbonia. lo scavo della tomba 12. notizia preliminare. *Quaderni Soprintendenza Cagliari* **26** (2015).

[9] Melis, M. G. *L'età del rame in Sardegna: origine ed evoluzione degli aspetti autoctoni* (Soter, Villanova Monteleone, 2000).

[10] Foschi Nieddu, A. Dalla tomba IV di Filigosa (Macomer, Nuoro) un contributo alla conoscenza delle ceramiche punteggiate dell'età del Bronzo. In *Atti della XXXI Riunione Scientifica dell'Istituto Italiano di Preistoria e Protostoria "La Valle d'Aosta nel quadro della Preistoria e Protostoria dell'arco alpino centro-occidentale" (Courmayeur, 2-5 giugno 1994)*, 535–547 (Istituto Italiano di Preistoria e Protostoria, Florence, 1997).

[11] Foschi Nieddu, A. *La Tomba 1 di Filigosa (Macomer, Nuoro) Alcune considerazioni sulla cultura di Abealzu-Filigosa nel contesto eneolitico della Sardegna* (Cooperativa grafica nuorese, Nuoro, 1986).

[12] Ugas, G. & Jossu, P. Tomba ipogeica ed elementi di cultura materiale delle fasi Campaniforme a e B. In Ugas, G. (ed.) *Ricerche archeologiche nel territorio di Sanluri*, 19–26 (Gruppo archeologico giovanile ex lege 285/77 Comune di Sanluri/ Soprintendenza

Archeologica per le Provincie di Cagliari e Oristano, Ricerche archeologiche nel territorio di Sanluri, 1982).

[13] Ugas, G. Facies campaniformi dell'ipogeo di Padru Jossu. In Nicolis, F. & Mottes, E. (eds.) *Il bicchiere campaniforme e l'Italia nella preistoria europea del III millennio a.C.*, 261–280 (Provincia Autonoma di Trento, Servizio Beni Culturali, Ufficio Beni Archeologici, 1998).

[14] León, J. M. M., Pau, C. & Guilaine, J. The proboscidean ivory adornments from the hypogeum of padru jossu (sanluri, sardinia, italy) and the mediterranean bell beaker. *Zephyrus* **82**, 35–64 (2018).

[15] Germanà, F. II gruppo umano nuragico di S'Iscia 'e sas Piras (Usini-Sassari) (antropologia e paleopatologia). *Studi Sardi* **23**, 53–124 (1973).

[16] Maxia, C. Osservazioni sul materiale scheletrico di una grotta funeraria nuragica a Perdasdefogu. In *Atti della VIII e IX Riunione Scientifica dell'Istituto Italiano di Preistoria e Protostoria, Trieste, 19-20 October 1963 – Calabria, 6-8 April 1964*, 157–163 (Istituto Italiano di Preistoria e Protostoria, Trieste, 1964).

[17] Cosseddu, G. G., Floris, G. & Sanna, E. Verso una revisione dell'inquadramento cronologico e morfometrico delle serie scheletriche paleo-protosarde i: Craniometria, primi dati. *Rivista di Antropologia* **72**, 153–162 (1994).

[18] Foschi Nieddu, A. I risultati degli scavi 1981 nella necropoli prenuragica di serra crabiles, sennori (sassari). In Waldren, W. H., Chapman, R., Lewthwaite, J. & Kennard, R. C. (eds.) *The Deya Conference of Prehistory. Early Settlement in the Western Mediterranean Islands and the Peripheral Areas, British Archaeological Reports 229*, 533–552 (British Archaeological Reports, 1984).

[19] Rovina, D. Necropoli preistorica: Sennori – Sassari, loc. Serra Crabiles. *Bollettino di Archeologia* **43/45**, 105–106 (1994).

[20] Meloni, G. M. Ricerche archeologiche nelle località di Corona Moltana e Zarau. In Conca C. (ed.) *Bonnanaro e il suo patrimonio culturale*, 90–99 (Segnavia, Sassari, 2004).

[21] Demartis, G. M. *Tomba V di Montalè Necropoli di Su Crucifissu Mannu. Sassari* (Betagamma, 1998).

[22] Ceruti, M. L. F. La tomba XVI di Su Crocifissu Mannu e la cultura di Bonnanaro,. *Bullettino di Paletnologia Italiana* **81 (1972-74)**, 113–218 (1976).

[23] Skeates, R., Gradoli, M. G. & Beckett, J. The cultural life of caves in Seulo, central Sardinia. *Journal of Mediterranean Archaeology* **26**, 97–126 (2013).

[24] Olivieri, A. *et al.* Mitogenome diversity in Sardinians: A genetic window onto an island's past. *Mol. Biol. Evol.* **34**, 1230–1239 (2017).

[25] Sanna, E., Liguori, A., Fagioli, M. B. & Floris, G. Verso una revisione dell'inquadramento cronologico e morfometrico delle serie scheletriche paleo-protosarde II: cranometria, ulteriori aggiornamenti. *Archivio per l'Antropologia e l'Etnologia* **79**, 239–250 (1999).

[26] Gradoli, M. G. & Meaden, T. Underworld and Neolithic rituality: the rock art of the Su Longu Fresu cave in central sardinia. In Anati, E. (ed.) *Art and Communication in Pre-Literate Societies*, 220–224 (Centro Camuno di Studi Preistorici, Capo di Ponte, 2011).

[27] Mazzarello, V., Piga, G., Delogu, P. L., Cecchini, A. & P., B. Analisi antropologica e molecolare sui resti scheletrici appartenenti alla tomba III della necropoli di Noeddale (Ossi). In *XVII Congresso degli Antropologi Italiani Cagliari 26-29 settembre*, 26–29 (2007).

[28] Ferrarese Ceruti, M. L. La tomba XVI di Su Crocifissu Mannu e la cultura di Bonnanaro. In *Bullettino di Paletnologia Italiana* (81 (1972-74), Firenze, 1976).

[29] Ferrarese Ceruti, M. L. Sisaia. una deposizione in grotta della Cultura di Bonnannaro. In *Quaderni Soprintendenza Sassari* (6, Sassari, 1978).

[30] Germanà, F. Crani della 1a età del Bronzo di S'Isterridolzu-Ossi nel contesto umano paleosardo recente (Antropologia e Paleopatologia). In *Atti del XX Congresso Internazionale d'Antropologia e Archeologia Preistorica, Cagliari 9-12 ottobre*, 377–384 (Università di Cagliari, Cagliari, 1980).

[31] Paderi, M. C. & A. Siddu, G. U. Ricerche nell'abitato di Mara. Notizia preliminare sull'area della necropoli di San Pietro. In Murgia, G. (ed.) *Villamar. Una comunità, la sua storia*, 121–157 (Grafica del Parteolla, Dolianova, 1993).

[32] Pompianu, E. Nuovi scavi nella necropoli punica di Villamar (2013-2015). *Fasti On Line Documents & Research* **395** (2017).

[33] Pompianu, E. & Murgia, C. Nuovi scavi nella necropoli punica di Villamar. un primo bilancio delle ricerche 2013-2015. In Serreli, G., Melis, R., French, C. & Sulas, F. (eds.) *Sa Massarìa: ecologia storica dei sistemi di lavoro contadino in Sardegna. (Europa e Mediterraneo. Storia e immagini di una comunità internazionale 37)*, 455–504 (CNR, Cagliari, 2017).

[34] Guirguis, M. & Unali, A. La fondazione di Sulky tra IX e VIII sec. a.c.: riflessioni sulla cultura materiale dei più antichi livelli fenici (area del Cronicario - Settore II - scavi 2013-2014). In A. Cazzella, F. N., A. Guidi (ed.) *Ubi Minor... Le isole minori del Mediterraneo centrale dal Neolitico ai primi contatti coloniali. Atti del Convegno di Studi in ricordo di Giorgio Buchner, a 100 anni dalla nascita (1914-2014) Anacapri-Capri, 27-28 ottobre 2014. Scienze dell'Antichità, 22*, 81–96 (Quasar, Roma, 2016).

[35] Bartoloni, P. Monte Sirai. In *Sardegna archeologia Guide e itinerari 10* (Carlo Delfino, Sassari, 2004).

[36] Guirguis, M. Storia degli studi e degli scavi a Sulky e a Monte Sirai. *Rivista di Studi Fenici* **33**, 13–29 (2005).

[37] Guirguis, M. *Monte Sirai. 1963-2013 mezzo secolo di indagini archeologiche*, vol. 53 of *Studi di Storia Antica e di Archeologia* (Carlo Delfino editore, Sassari, 2013).

[38] Guirguis, M. Dinamiche sociali e cultura materiale a Sulky e Monte Sirai. In P. van Dommelen, A. R. (ed.) *Materiali e contesti nell'età del Ferro sarda. Atti della giornata di studi (Museo Civico di San Vero Milis, Oristano, 25 maggio 2012). (Rivista di Studi Fenici 41, 1-2 [2013])* (Fabrizio Serra Editore, Pisa-Roma, 2014).

[39] Bartoloni, P. La necropoli di Monte Sirai - I. In *Collezione di Studi Fenici 41* (CNR, Rome, 2000).

[40] Guirguis, M. *Necropoli fenicia e punica di Monte Sirai. Indagini archeologiche 2005-2007*, vol. 7 of *Studi di Storia Antica e di Archeologia* (Sandhi, Ortacesus, 2010).

[41] Guirguis, M. Gli spazi della morte a Monte Sirai (Carbonia-Sardegna). rituali e ideologie funerarie nella necropoli fenicia e punica (scavi 2005-2010). *Fasti On Line Documents & Research* **230** (2011).

[42] Guirguis, M., Piga, G. & Allué, E. Funerary rituals and ideologies in the Phoenician-Punic necropolis of Monte Sirai (Carbonia, Sardinia, Italy). In Thompson, T. (ed.) *The Archaeology of Cremation: Burned human remains in funerary studies. (Studies in Funerary Archaeology, 8).*, 97–122 (Oxbow Books, Oxford, 2015).

[43] Guirguis, M. R. P. O. & Pompianu, E. Premature deaths in Punic Sardinia. the perception of childhood in funerary contexts at Monte Sirai and Villamar. In Tabolli, J. (ed.) *From Invisible to Visible. New Methods and Data for the Archaeology of Infant and Child Burials in Pre-Roman Italy and Beyond. (Studies in Mediterranean Archaeology 149)*, 207–217 (Astrom Editions, 2018).

[44] Piga, G., Guirguis, M., Bartoloni, P., Malgosa, A. & Enzo, S. A funerary rite study of the Phoenician-Punic necropolis of Mount Sirai (Sardinia, Italy). *International Journal of Osteoarchaeology* **20**, 144–157 (2010).

[45] Guirguis, M., Murgia, C. & Pla Orquín, R. Archeoantropologia e bioarcheologia nella necropoli di Monte Sirai (Carbonia-Italia). risultati delle analisi su alcuni contesti della prima età Punica (fine VI-inizi IV sec. a.c.). In Serra, F. (ed.) *From the Mediterranean to the Atlantic: People, Goods and Ideas between East and West. Proceedings of the 8th International Congress of Phoenician and Punic Studies (Italy, Sardinia-Carbonia, Sant'Antioco, 21-26 October 2013). (Folia Phoenicia, 1).*, 282–299 (Fabrizio Serra Editore, Pisa-Roma, 2017).

[46] Matisoo-Smith, E. *et al.* Ancient mitogenomes of Phoenicians from Sardinia and Lebanon: A story of settlement, integration, and female mobility. *PLoS One* **13**, e0190169 (2018).

[47] Rovina, D. & Fiori M. Olia, P. Il Duomo e il cimitero di San Nicola. In Rovina D., F. M. (ed.) *Sassari : Archeologia Urbana*, 120–129 (Felici Editore, 2013).

[48] La Fragola, A. Instrumenta scriptoria da sepoltura di età romana a cremazione. In *Studi di Antichità 13*, 243–252 (Università degli Studi del Salento, 2016).

[49] La Fragola, A. Essere scolari nella Sardegna romana. *Archeo rivista* **370**, 12–13 (2015).

[50] La Fragola, A. Il dio sfuggente. *Archeo rivista* **361**, 56–65 (2015).

[51] La Fragola, A. Lo scavo della necropoli romana di Monte Carru ad Alghero. *Aidu Entos - Archeologia e Beni Cult* **2**, 4–6 (2008).

[52] Skoglund, P. *et al.* Separating endogenous ancient dna from modern day contamination in a siberian neandertal. *Proceedings of the National Academy of Sciences* **111**, 2229–2234 (2014).

[53] Al-Asadi, H., Dey, K. K., Novembre, J. & Stephens, M. Inference and visualization of DNA damage patterns using a grade of membership model. *Bioinformatics* **35**, 1292–1298 (2018).

[54] Ginolhac, A., Rasmussen, M., Gilbert, M. T. P., Willerslev, E. & Orlando, L. mapDamage: testing for damage patterns in ancient DNA sequences. *Bioinformatics* **27**, 2153–2155 (2011).

[55] Jónsson, H., Ginolhac, A., Schubert, M., Johnson, P. L. & Orlando, L. mapdamage2. 0: fast approximate bayesian estimates of ancient DNA damage parameters. *Bioinformatics* **29**, 1682–1684 (2013).

[56] Francalacci, P. *et al.* Low-pass DNA sequencing of 1200 Sardinians reconstructs European Y-chromosome phylogeny. *Science* **341**, 565–569 (2013).

[57] Olalde, I. *et al.* The Beaker phenomenon and the genomic transformation of Northwest Europe. *Nature* **555**, 190 (2018).

[58] D'Atanasio, E. *et al.* The peopling of the last Green Sahara revealed by high-coverage resequencing of trans-saharan patrilineages. *Genome Biology* **19**, 20 (2018).

[59] Haak, W. *et al.* Massive migration from the steppe was a source for Indo-European languages in Europe. *Nature* **522**, 207–211 (2015).

[60] Mathieson, I. *et al.* The genomic history of southeastern Europe. *Nature* **555**, 197 (2018).

[61] González, M. *et al.* The genetic landscape of Equatorial Guinea and the origin and migration routes of the Y chromosome haplogroup R-V88. *European Journal of Human Genetics* **21**, 324 (2013).

[62] Haber, M. *et al.* Chad genetic diversity reveals an African history marked by multiple Holocene Eurasian migrations. *The American Journal of Human Genetics* **99**, 1316–1324 (2016).

[63] Zilhão, J. Radiocarbon evidence for maritime pioneer colonization at the origins of farming in west Mediterranean Europe. *Proceedings of the National Academy of Sciences* **98**, 14180–14185 (2001).

[64] Fregel, R. *et al.* Ancient genomes from North Africa evidence prehistoric migrations to the Maghreb from both the Levant and Europe. *Proceedings of the National Academy of Sciences* **115**, 6774–6779 (2018).

[65] Harney, É. *et al.* Ancient DNA from Chalcolithic Israel reveals the role of population mixture in cultural transformation. *Nature Communications* **9**, 3336 (2018).

[66] Lazaridis, I. *et al.* Genetic origins of the Minoans and Mycenaeans. *Nature* **548**, 214–218 (2017).

[67] Rodríguez-Varela, R. *et al.* Genomic analyses of Pre-European Conquest human remains from the Canary Islands reveal close affinity to modern North Africans. *Current Biology* **27**, 3396–3402 (2017).

[68] Fernandes, D. M. *et al.* The arrival of Steppe and Iranian related ancestry in the islands of the Western Mediterranean. *bioRxiv* 584714 (2019). https://doi.org/10.1101/584714.

[69] Pritchard, J. K., Stephens, M. & Donnelly, P. Inference of population structure using multilocus genotype data. *Genetics* **155**, 945–959 (2000).

[70] Alexander, D. H., Novembre, J. & Lange, K. Fast model-based estimation of ancestry in unrelated individuals. *Genome Research* **19**, 1655–1664 (2009).

[71] Joseph, T. A. & Pe'er, I. Inference of population structure from time-series genotype data. *The American Journal of Human Genetics* (2019).

[72] Lee, S., Zou, F. & Wright, F. A. Convergence and prediction of principal component scores in high-dimensional settings. *Annals of Statistics* **38**, 3605 (2010).

[73] Wang, C., Zhan, X., Liang, L., Abecasis, G. R. & Lin, X. Improved ancestry estimation for both genotyping and sequencing data using projection procrustes analysis and genotype imputation. *The American Journal of Human Genetics* **96**, 926–937 (2015).

[74] Liu, L. T., Dobriban, E., Singer, A. *et al.* e pca: High dimensional exponential family pca. *The Annals of Applied Statistics* **12**, 2121–2150 (2018).

[75] Patterson, N., Price, A. L. & Reich, D. Population structure and eigenanalysis. *PLoS Genetics* **2**, e190 (2006).
