# Supplementary material for: Genetic history from the Middle Neolithic to present on the Mediterranean island of Sardinia
Source: Nat Commun. 2020 Feb 24;11:939. doi: 10.1038/s41467-020-14523-6 (PMC7039977; doi:10.1038/s41467-020-14523-6)
Supplement: Supplementary file 6 — Reporting Summary [file 41467_2020_14523_MOESM6_ESM.pdf]

nature research

Corresponding author(s): John Novembre, Francesco Cucca, and Johannes Krause

Last updated by author(s): Dec 13, 2019

Reporting Summary

Nature Research wishes to improve the reproducibility of the work that we publish. This form provides structure for consistency and transparency in reporting. For further information on Nature Research policies, see Authors & Referees and the Editorial Policy Checklist .

Statistics

For all statistical analyses, confirm that the following items are present in the figure legend, table legend, main text, or Methods section.

n/a	Confirmed	
☐	☒	The exact sample size (*n*) for each experimental group/condition, given as a discrete number and unit of measurement
☐	☒	A statement on whether measurements were taken from distinct samples or whether the same sample was measured repeatedly
☐	☒	The statistical test(s) used AND whether they are one- or two-sided<br>*Only common tests should be described solely by name; describe more complex techniques in the Methods section.*
☒	☐	A description of all covariates tested
☐	☒	A description of any assumptions or corrections, such as tests of normality and adjustment for multiple comparisons
☐	☒	A full description of the statistical parameters including central tendency (e.g. means) or other basic estimates (e.g. regression coefficient) AND variation (e.g. standard deviation) or associated estimates of uncertainty (e.g. confidence intervals)
☐	☒	For null hypothesis testing, the test statistic (e.g. *F*, *t*, *r*) with confidence intervals, effect sizes, degrees of freedom and *P* value noted<br>*Give P values as exact values whenever suitable.*
☐	☒	For Bayesian analysis, information on the choice of priors and Markov chain Monte Carlo settings
☒	☐	For hierarchical and complex designs, identification of the appropriate level for tests and full reporting of outcomes
☒	☐	Estimates of effect sizes (e.g. Cohen's *d*, Pearson's *r*), indicating how they were calculated

Our web collection on statistics for biologists contains articles on many of the points above.

Software and code

Policy information about availability of computer code

Data collection	No software was used
Data analysis	To analyze the data generated in this study and downloaded from publicly available resources, we used a combination of standard bioinformatic command line tools, software packages, and custom scripts. To the extent possible, we used a self-contained conda (version=4.4.6) environment to install and track the exact software versions and dependencies used in each analysis. We often took advantage of the “bioconda” channel which provided seamless and reproducible installation of a number of standard bioinformatic tools. For the packages and tools not maintained by conda we compiled them manually from source. We also used pip (version=9.0.1) to install python package not managed by conda and for R (version=3.5.1) packages we used the base function “install.packages()” or devtools function “install_github()”.<br><br>We organized the analysis into a python (version=3.6.4) Snakemake (version=4.4.0) pipeline which managed submission of jobs to our high performance computing cluster (https://rcc.uchicago.edu/docs/using-midway/index.html#overview) and tracked file dependencies for multi-step analyses. Here we describe the software tools used in this study in the context of each modular analysis:<br><br>Raw Sequence Analysis:<br>The raw sequence data was processed up to the aligned reads using the “efficient ancient genome reconstruction” (EAGER, version=1.92.55) pipeline (Peltzer et al., 2016). Specifically, we used AdapterRemoval (version=2.2.0), BWA aln/samse programs (version=0.7.12), and DeDup (version=0.12.2). See (Peltzer et al., 2016), (https://github.com/apeltzer/EAGER-GUI), and (https://eager.readthedocs.io/en/latest/) for details on each step in the pipeline.<br><br>Additional Data Quality Control:<br>From the aligned reads generated by the “EAGER” pipeline we then removed sequence data from the last three base pairs on both sides of every read using the “trimBam” sub-tool of “BamUtil” (version=1.0.14). For downstream processing and indexing of filtered bam files

we used “samtools” (version=1.7).

Contamination:
To estimate modern contamination levels for the data generated in this study we used “schmutzi” (https://github.com/grenaud/schmutzi) for mtDNA-based estimates and “ANGSD” (https://github.com/ANGSD/angsd) for X chromosome based estimates.

Damage Estimation:
We used the R package “aRchaic” (https://github.com/kkdey/aRchaic) to estimate and visualize DNA damage patterns typically observed in ancient DNA datasets. We also used “mapdamage” (version=2.0) as a complementary approach to estimate damage profiles in each ancient individual. Finally, we used “pmdtools” (https://github.com/pontussk/PMDtools, release=0.60) to estimate damage scores for each read.

Genotype Calling:
We used custom scripts (https://github.com/mathii/gdc3) to randomly sample reads and generate pseudo-haploid genotype calls as described in materials and methods.

Genotype Merging:
To robustly merge genotypes from heterogeneous data sources and types, including the genotype calls we directly made and contemporary genotyping array data we downloaded, we used the “conform-gt” java program downloaded from the BEAGLE website (https://faculty.washington.edu/browning/conform-gt/conform-gt.24May16.cee.jar). We also used “convertf”, “mergeit” (https://github.com/DReichLab/EIG, release=v7.2.1) and “plink1.9” (https://www.cog-genomics.org/plink2) to convert and merge different, commonly used, file formats that store genotype data. Once the genotype data was merged, we stored a large HDF5 file using utilities from “scikit-allel” (version=1.1.10) for fast access and sub-setting of individuals and SNPs.

PCA:
To compute the truncated SVD of our data matrix we used the python module “scipy” (version=1.0.0). We also developed a modular python package called “pcshrink” to implement the jackknife procedure for correcting the bias of out of sample PC score predictions (https://github.com/jhmarcus/pcshrink). We compared the correction from “pcshrink” to other related approaches implemented in “smartpca” (version=13050).

f-statistics/fst:
To compute estimators of f3/f4 statistics, fst and their corresponding standard errors we used the python module “scikitallel” (version=1.1.10).

ADMIXTURE:
We used the software “ADMIXTURE” (version=1.3.0) to obtain maximum likelihood estimates of individual admixture proportions.

DyStruct:
We used the software “DyStruct” (https://github.com/tyjo/dystruct, release=v1.0.0) to obtain Bayesian estimates of individual admixture proportions.

qpAdm:
To test specific hypothesis about population level admixture and estimate corresponding admixture proportions we used “qpAdm” (version=810) which is part of the “AdmixTools” stack of population genetics tools.

For manuscripts utilizing custom algorithms or software that are central to the research but not yet described in published literature, software must be made available to editors/reviewers. We strongly encourage code deposition in a community repository (e.g. GitHub). See the Nature Research guidelines for submitting code & software for further information.

Data

Policy information about availability of data

All manuscripts must include a data availability statement. This statement should provide the following information, where applicable:

- Accession codes, unique identifiers, or web links for publicly available datasets
- A list of figures that have associated raw data
- A description of any restrictions on data availability

The aligned sequences and called genotypes from the data generated from this study will be available through the European Nucleotide Archive (ENA) before publication under accession number PRJEB35094. The contemporary Sardinia data used to support this study have allele frequency summary data deposited to EGA under accession number EGAS00001002212. The disaggregated individual level sequence data for 1,577 used in this study is a subset of 2,105 samples (adult volunteers of the SardiNIA cohort longitudinal study) from Sidore et al (2015) and are available from dbGAP under project identifier phs000313 (v4.p2). The remaining individual-level sequence data are from a case-control study of autoimmunity from across Sardinia, and per the obtained consent and local IRB, these data are only available for collaboration by request from the project leader (Francesco Cucca, Consiglio Nazionale delle Ricerche, Italy).

Genotypes from contemporary reference individuals from Western Eurasia were downloaded from the Human Origins Array dataset posted to the Reich Lab website (https://reich.hms.harvard.edu/sites/reich.hms.harvard.edu/files/inline-files/EuropeFullyPublic.tar.gz). We also downloaded publicly available ancient DNA capture data from a number of published studies (Mathieson et al., 2015; Lazaridis et al., 2016, 2017; Mathieson et al., 2018, 2017; Lipson et al., 2017; Olalde et al., 2018). Below we post the accession numbers for the aligned reads we used to download each dataset:

Mathieson et al., 2015: PRJEB11450
Mathieson et al., 2018: PRJEB22652

Lazaridis et al., 2016: PRJEB14455
Lazaridis et al., 2017: PRJEB20914
Lipson et al., 2017: PRJEB22629
Olalde et al., 2018: PRJEB23635

We also used ancient genotypes from a public database compiled by the Reich lab (https://reich.hms.harvard.edu/downloadable-genotypes-present-day-and-ancient-dna-data-compiled-published-papers)

Field-specific reporting

Please select the one below that is the best fit for your research. If you are not sure, read the appropriate sections before making your selection.

☐ Life sciences ☐ Behavioural & social sciences ☒ Ecological, evolutionary & environmental sciences

For a reference copy of the document with all sections, see nature.com/documents/nr-reporting-summary-flat.pdf

Ecological, evolutionary & environmental sciences study design

All studies must disclose on these points even when the disclosure is negative.

Study description
The study investigates the population history of Sardinia using ancient DNA data.

Research sample
Our sample consists of 70 ancient DNA samples that were combined with previously published datasets.

Sampling strategy
The sample sizes in ancient DNA studies are limited by the availability and preservation status of skeletal remains - we sought to maximize the sample sizes given this constraint. In ancient DNA studies, insights have been gained from even single influential samples when genome-wide SNP markers are used.

Data collection
The archaeological samples used in this project derive from several collection avenues. The first was a sampling effort led by co-author Luca Lai, leveraging a broad base of samples from different existing collections in Sardinia, a subset of which were previously used in isotopic analyses to understand dietary composition and change in prehistoric Sardinia . The second was from the Seulo Caves project, an on-going project on a series of caves that span the Middle Neolithic to late Bronze Age near the town of Seulo. The project focuses on the diverse forms and uses of caves in the prehistoric culture of Sardinia. The Neolithic individuals from Sassari province as well as the post-Nuragic individuals were collected from several co-authors as indicated in Supplemental Information Section 1. The third was a pair of Neolithic sites Noedalle and S'isterridolzu. The fourth are a collection of post-Nuragic sites spanning from the Phoenician to the Medieval time.

Timing and spatial scale
The estimated ages in our sample range from 4,100 years BCE to 1,500 years CE . Spatially the samples cluster around the Seulo Caves and northwestern Sardinia, with a sporadic set of additional samples.

Data exclusions
Samples were excluded on the basis of: 1) Insufficient endogenous DNA during initial screening; 2) Evidence of modern human contamination; 3) Insufficient number of sequence reads from final sequencing runs.

Reproducibility
Initial DNA isolates are preserved and available for possible re-sequencing experiments via contact with the authors.

Randomization
Samples were processed for sequencing in batches upon receipt. Each batch of samples contained a range of sample ages.

Blinding
We did not use any formal blinding procedures, though the data collection was carried out independently from the population genetic analyses.

Did the study involve field work? ☒ Yes ☐ No

Field work, collection and transport

Field conditions
The field work consisted of archaeological sampling, that took place over several sampling expeditions. Weather conditions varied during the fieldwork; though when rain was present, care was taken to avoid exposing skeletal remains.

Location
The field work took place in a series of archaeological sites. Supplemental Section 1 contains the explicit latitude and longitudes of each site.

Access and import/export
All samples were handled in collaboration with local scientists and with the approval of the local Sardinian authorities for the handling of archaeological samples (Ministero per i Beni e le Attivita Culturali, Direzione Generale per i `beni Archeologici, request dated 11 August 2009; Soprintendenza per i Beni Archeologici per le province di Sassari e Nuoro, prot. 12993 dated 20 Dec. 2012; Soprintendenza per i Beni Archeologici per le province di Sassari e Nuoro, prot. 10831 dated 27 Oct. 2014;Soprintendenza per i Beni Archeologici per le province di Sassari e Nuoro, prot. 12278 dated 05 Dec. 2014; Soprintendenza per i Beni Archeologici per le Provincie di Cagliari e Oristano, prot. 62, dated 08 Jan 2015; Soprintendenza Archeologia, Belle Arti e Paesaggio per le Provincie di Sassari, Olbia-Tempio e Nuoro, prot. 4247 dated 14 March 2017; Soprintendenza per i Beni Archeologici per le Provincie di Sassari e Nuoro, prot. 12930 dated 30 Dec. 2014; Soprintendenza Archeologia, Belle arti e

Paesaggio per le Provincie di Sassari e Nuoro, prot. 7378 dated 9 May, 2017; Soprintendenza Archeologia, Belle Arti e Paesaggio per le Provincie di Sassari e Nuoro, prot. 15796 dated 25 October, 2017; Soprintendenza Archeologia, Belle Arti e Paesaggio per le Provincie di Sassari e Nuoro, prot. 16258 dated 26 Nov. 2017; Soprintendenza per i Beni Archeologici per le province di Sassari e Nuoro, prot. 5833 dated 16 May 2018; Soprintendenza Archeologia, Belle Arti e Paesaggio per la citta metropolitana di Cagliari e le province di Oristano e Sud Sardegna, prot.~30918 dated 10 Dec 2019). For more, detailed description of the sites please see Supplemental Information Section 1.

Disturbance

All sampling practices were carried out in accordance with the protocols approved by local Sardinian authorities (see access statement above) and include return of original samples to local repositories. When possible, the location of skeletal remains was mapped prior to extraction such that taphonomic and positional information was not disturbed in the sampling.

Reporting for specific materials, systems and methods

We require information from authors about some types of materials, experimental systems and methods used in many studies. Here, indicate whether each material, system or method listed is relevant to your study. If you are not sure if a list item applies to your research, read the appropriate section before selecting a response.

Materials & experimental systems

n/a	Involved in the study
☒	☐ Antibodies
☒	☐ Eukaryotic cell lines
☒	☐ Palaeontology
☒	☐ Animals and other organisms
☒	☐ Human research participants
☒	☐ Clinical data

Methods

n/a	Involved in the study
☒	☐ ChIP-seq
☒	☐ Flow cytometry
☒	☐ MRI-based neuroimaging
