# Supplementary material for: Genetic history from the Middle Neolithic to present on the Mediterranean island of Sardinia
Source: Nat Commun. 2020 Feb 24;11:939. doi: 10.1038/s41467-020-14523-6 (PMC7039977; doi:10.1038/s41467-020-14523-6)
Supplement: Supplementary file 7 — Description of Additional Supplementary Files [file 41467_2020_14523_MOESM7_ESM.pdf]

Title: Supplementary Data 1:
Description: Excel File containing 6 sheets describing meta information and results for the 70 genotyped ancient individuals as well as reference individuals included in this study. Importantly, sheet A contains relevant meta information for each newly genotyped individual. The symbol "_" in the ID column indicates that two libraries have been merged - as three pairs of individuals turned out to be genetically identical (each pair from the same site and with overlapping radiocarbon dates).

Title: Supplementary Data 2:
Description: Excel file summarizing f-statistics results.

Title: Supplementary Data 3:
Description: Excel file summarizing individual qpAdm results with 2-5 proximal sources.
